# Comparative efficacy of brain-computer interface training with different feedback modalities for post-stroke upper-limb motor recovery: a systematic review and network meta-analysis of randomized controlled trials

**DOI:** 10.3389/fnins.2026.1925602

**Published:** 2026-10-16

**Authors:** Fuqiang Wang, Xiao Lu, Yunhong Deng, Yaojiang Li, Xiaodi Li, Congping Huang, Mengliang Ma, Bianhong He, Lang Yu, Lixia Deng

**Affiliations:** 1 Department of Rehabilitation Medicine, Guangdong Sanjiu Brain Hospital, Guangzhou, Guangdong, China; 2 Sanjiu Brain Disease and Rehabilitation Hospital Affiliated to South China Normal University, Guangzhou, Guangdong, China; 3 Department of Rehabilitation Therapy, Guangdong Sanjiu Brain Hospital, Guangzhou, Guangdong, China; 4 Clinical Medical College of Acupuncture Moxibustion and Rehabilitation, Guangzhou University of Chinese Medicine, Guangzhou, Guangdong, China; 5 Institute for Brain Research and Rehabilitation, South China Normal University, Guangzhou, Guangdong, China

**Keywords:** brain-computer interface, feedback modality, network meta-analysis, neurorehabilitation, stroke, upper-limb motor recovery

## Abstract

**Background:**

Brain-computer interface (BCI) training may improve post-stroke upper-limb recovery through closed-loop feedback, but the comparative efficacy of different feedback modalities remains uncertain. This study compared BCI training with different feedback modalities for post-stroke upper-limb motor impairment.

**Methods:**

PubMed, Embase, Web of Science, Cochrane Library, and Scopus were searched from inception through April 2026 for randomized controlled trials of BCI-based upper-limb rehabilitation after stroke. BCI interventions were classified by feedback modality and feedback contingency into multimodal feedback (BCI-MM), robotic or exoskeleton-assisted feedback (BCI-Robot), functional electrical stimulation feedback (BCI-FES), and visual feedback (BCI-visual), while comparator interventions were classified separately, with usual rehabilitation (UR) as the reference. Random-effects network meta-analysis estimated WMDs with 95% CIs. The primary outcome was Fugl-Meyer Assessment for Upper Extremity (FMA-UE); secondary outcomes were the Action Research Arm Test (ARAT) and Wolf Motor Function Test (WMFT). Risk of bias was assessed using the Cochrane Risk of Bias 2 tool, and certainty was assessed using CINeMA.

**Results:**

Twenty-two trials involving 956 patients across subacute, chronic, and mixed post-stroke stages were included. The FMA-UE network contained 11 connected nodes, with no global or local inconsistency. Compared with UR, BCI-Robot showed greater FMA-UE improvement (WMD = 6.28, 95% CI 2.70–9.86), as did non-BCI robotic training (WMD = 4.26, 95% CI 0.42–8.10) and BCI-MM (WMD = 4.03, 95% CI 1.00–7.06). BCI-FES did not differ significantly from UR. BCI-visual had the highest SUCRA value (77.6%), but its comparison with UR was non-significant, with very low certainty of evidence; no BCI feedback modality was superior to another. Secondary outcomes showed greater ARAT improvement with BCI-Robot versus UR, whereas WMFT findings were non-significant. Certainty for the main FMA-UE comparisons was low or very low.

**Conclusion:**

BCI-Robot and BCI-MM showed statistically significant FMA-UE benefits versus UR, although certainty was low. As non-BCI robotic training also showed benefit, the BCI-Robot effect cannot be attributed to BCI alone. No BCI feedback modality was statistically superior to another. Further high-quality direct-comparison trials are needed.

**Systematic review registration:**

https://www.crd.york.ac.uk/PROSPERO/view/CRD420261370474, Unique Identifier: CRD420261370474.

## 1 Introduction

Stroke remains a major global cause of death and long-term disability. With the increasing global burden of stroke and continued improvements in acute stroke care and overall stroke management, long-term functional impairments and rehabilitation needs among stroke survivors have become more prominent (GBD 2021 Stroke Risk Factor Collaborators, 2024; Marín-Medina et al., 2024). Upper-limb motor impairment is one of the most common post-stroke sequelae and substantially affects activities of daily living. Upper-limb function requires coordinated multi-joint control of the shoulder, elbow, wrist, and hand, as well as complex functions such as grasping, releasing, object manipulation, and fine motor control. Post-stroke upper-limb motor recovery is closely related to neuroplasticity, reorganization of sensorimotor networks, and stroke stage, and may be influenced by the dose of task-specific training and the timing of intervention (Dromerick et al., 2021; Marín-Medina et al., 2024). Therefore, further improving upper-limb motor function beyond conventional rehabilitation remains an important challenge in neurorehabilitation, particularly through strategies that enhance patients’ active engagement and promote reorganization of sensorimotor networks.

Brain-computer interface (BCI) offers a training strategy for post-stroke upper-limb rehabilitation that targets central motor intention (Daly and Wolpaw, 2008; Young et al., 2021). BCI systems can acquire and decode brain activity related to motor imagery (MI) or attempted movement and translate these signals into external feedback or control signals for assistive devices. This establishes a closed loop between motor intention, feedback output, and sensory reafference (Daly and Wolpaw, 2008; Young et al., 2021; Mansour et al., 2022; Li et al., 2025). Unlike purely passive training or conventional repetitive training, BCI training is characterized by temporal coupling between the patient’s active motor intention and external feedback. This process may promote sensorimotor rhythm modulation, reorganization of motor representations, and activity-dependent neuroplasticity (Daly and Wolpaw, 2008; Cervera et al., 2018; Young et al., 2021; Mansour et al., 2022). For patients with limited voluntary motor capacity or an inability to produce overt limb movement, BCI can still drive feedback by detecting brain activity related to motor intention. Therefore, BCI may have clinical value for the rehabilitation of moderate-to-severe upper-limb impairment (Daly and Wolpaw, 2008; Cervera et al., 2018; Mansour et al., 2022; Zhang et al., 2024; Li et al., 2025).

However, BCI should not be regarded as a single, homogeneous rehabilitation intervention. In studies of post-stroke upper-limb rehabilitation, BCI training is often combined with different feedback modalities or external effectors, including functional electrical stimulation (FES), robotic or exoskeleton-assisted feedback, visual feedback, virtual reality feedback, and multimodal sensory feedback (Baniqued et al., 2021; Mansour et al., 2022; Ren et al., 2024; Kim et al., 2025; Li et al., 2025; Liu et al., 2025; Lu et al., 2025). These modalities differ not only in how feedback is delivered but also in the source of sensory input, degree of motor execution, task immersion, and closed-loop pathway, which may reflect different rehabilitation mechanisms. BCI combined with FES can couple brain activity related to motor intention with target muscle contraction and peripheral proprioceptive input, thereby reinforcing the central-peripheral sensorimotor closed loop (Ren et al., 2024); BCI combined with robotic or exoskeleton assistance can provide motor execution feedback related to motor intention through a controllable robotic device (Baniqued et al., 2021); contingent feedback related to MI may be superior to non-contingent feedback and may influence upper-limb function and neuroplastic changes (Kim et al., 2025); visual or virtual reality feedback may enhance the vividness of MI through action observation, real-time performance feedback, and task-specific contexts, thereby increasing patient engagement (Mansour et al., 2022; Kim et al., 2025). Multimodal feedback may further enhance sensorimotor integration across networks by combining visual, tactile, and proprioceptive inputs and engaging multiple sensorimotor channels (Lu et al., 2025). Therefore, different BCI feedback modalities may produce different clinical effects, and pooling all BCI interventions may obscure these differences.

In recent years, the number of relevant randomized controlled trials (RCTs) and systematic reviews has increased, and available evidence generally suggests that BCI training may improve post-stroke upper-limb motor function (Mansour et al., 2022; Zhang et al., 2024; Li et al., 2025). Some studies further indicate that BCI design features, external feedback type, and training parameters may influence treatment effects (Mansour et al., 2022; Li et al., 2025). Previous meta-analyses have also examined feedback-related heterogeneity, but mainly through subgroup analyses rather than network comparisons. These syntheses were largely based on evidence available up to 2024 and generally treated different feedback modalities as subgroup categories rather than interconnected network nodes. Consequently, direct and indirect evidence was not combined to estimate relative effects across BCI feedback modalities, and their comparative efficacy within a common network framework remains uncertain (Mansour et al., 2022; Li et al., 2025). In clinical practice, rehabilitation teams need to decide not only whether to use BCI but also which feedback modality is most appropriate when resources are limited, device types vary, and patients differ substantially in functional status. Without evidence on the relative efficacy of different feedback modalities, the clinical use of BCI may remain largely focused on technical feasibility and may provide insufficient support for generalizable rehabilitation decision-making.

Network meta-analysis can synthesize direct and indirect evidence within a single statistical framework, allowing simultaneous comparison of multiple interventions and estimation of their relative ranking (Rouse et al., 2017). Because studies of BCI-based upper-limb rehabilitation involve diverse feedback modalities, often share common comparators, and provide limited direct head-to-head comparisons, network meta-analysis is well suited to evaluate comparative efficacy across feedback modalities. However, previous studies have generally evaluated BCI training as an overall intervention or focused on specific feedback modalities, such as BCI with robotic or exoskeleton-assisted feedback (BCI-Robot) or BCI with FES feedback (BCI-FES). Network evidence comparing BCI-based upper-limb rehabilitation interventions according to the primary therapeutic feedback modality and contingent closed-loop characteristics remains limited. Therefore, this study aimed to systematically evaluate the effects of BCI training with different feedback modalities on post-stroke upper-limb motor impairment. Using the Fugl-Meyer Assessment for Upper Extremity (FMA-UE) as the primary outcome and the Action Research Arm Test (ARAT) and Wolf Motor Function Test (WMFT) as secondary outcomes, we compared and ranked the relative efficacy of different BCI feedback modalities. These findings may inform the selection of BCI-based upper-limb rehabilitation strategies after stroke and the design of future RCTs with direct comparisons.

## 2 Methods

This systematic review and network meta-analysis was registered in PROSPERO (CRD420261370474). This study was reported in accordance with the PRISMA 2020 statement and the PRISMA-NMA extension statement (Hutton et al., 2015). The complete PRISMA-NMA checklist is provided in Appendix E1.

### 2.1 Eligibility criteria and intervention node classification

Eligibility criteria were defined according to the PICOS framework. Participants were patients with stroke confirmed clinically or radiologically and upper-limb motor impairment of any severity. No restrictions were applied to age, sex, stroke type, lesion location, or time since stroke. Eligible studies were limited to parallel-group RCTs reported as full-text, peer-reviewed original research articles published in English. The experimental intervention was BCI-based upper-limb rehabilitation training, defined as training that detects patients’ motor intention from electroencephalography (EEG) signals or other brain activity signals and provides closed-loop feedback through an external device or feedback system. Control interventions included usual rehabilitation (UR), usual care, sham BCI, no additional intervention, and other active rehabilitation interventions. The primary outcome was the FMA-UE score. Secondary outcomes included the ARAT and the WMFT.

Studies were excluded if the prespecified primary outcome data were unavailable or not extractable, stroke-specific data could not be extracted separately, the intervention did not meet the definition of BCI-based upper-limb rehabilitation, the BCI feedback modality could not be classified according to the prespecified node definitions, or additional unequal non-BCI co-interventions precluded isolation of the effect of BCI feedback training. Duplicate or overlapping reports from the same study cohort were also excluded.

Intervention nodes were classified according to the primary therapeutic feedback modality rather than device or system names alone. Contingent BCI feedback was defined as external feedback, assistive device movement, or therapeutic stimulation triggered, modulated, or driven by task-related brain activity decoded through a real-time BCI system. Prespecified BCI-related nodes included BCI with multimodal feedback (BCI-MM), BCI-Robot, BCI-FES, and BCI with visual feedback (BCI-visual). Non-BCI control or active intervention nodes were classified according to their clinical characteristics as UR, robotic or exoskeleton-assisted training without contingent BCI feedback (Robot), functional electrical stimulation without contingent BCI feedback (FES), or motor imagery (MI). Feedback that was non-contingent or non-specific was classified into the corresponding sham BCI node. Node classification was performed independently by two reviewers using the intervention descriptions in the original studies. Disagreements were resolved by discussion, with arbitration by a third reviewer when necessary. The complete node definitions, node classification rules, and study-level classification rationales are provided in Appendix E3.

### 2.2 Search strategy and study selection

PubMed, Embase, Web of Science, the Cochrane Library, and Scopus were searched systematically from database inception through April 2026. The search did not extend to clinical trial registries. The search strategy combined subject headings and free-text terms related to stroke, brain-computer interface, upper-limb function, and RCTs. The main search terms included “stroke,” “brain-computer interface,” “BCI,” “upper limb,” “upper extremity,” and “randomized controlled trial.” Search strategies were adapted for each database according to the relevant indexing system and search syntax. The complete search strategy is provided in Appendix E2.

Retrieved records were imported into EndNote 21 for reference management and deduplication. After duplicate records were removed, two reviewers independently screened the titles and abstracts. Full texts were retrieved for records that could not be clearly excluded and were assessed against the prespecified inclusion and exclusion criteria. Specific reasons for exclusion were recorded during full-text screening. Disagreements between the two reviewers were resolved through discussion, with arbitration by a third reviewer when necessary. The study selection process is presented using a PRISMA 2020 flow diagram.

### 2.3 Data extraction

Two reviewers independently extracted data using a predefined data extraction form. Extracted information included the first author, year of publication, country or region, sample size, patient age, sex, time since stroke, baseline upper-limb motor function, intervention details for the experimental and control groups, BCI feedback modality, training frequency, session duration, total treatment period, group sample sizes, and outcome values for the FMA-UE, ARAT, and WMFT.

Change scores from baseline to the end of the intervention were extracted as the primary analytic data. When multiple post-intervention time points were reported, the end-of-intervention assessment was used for the primary synthesis. When change scores were not directly reported but pre- and post-treatment means and standard deviations were available, or when standard errors, 95% confidence intervals, medians, interquartile ranges, or other convertible statistics were provided, data were converted using methods commonly applied in systematic reviews and meta-analyses (Follmann et al., 1992; Wan et al., 2014; Luo et al., 2018; Pearson and Smart, 2018; Cumpston et al., 2019). Details of the data conversion procedures and their study-specific application are provided in Appendix E4. If required outcome data were unavailable and could not be derived using the prespecified methods, no additional imputation was performed, and the study was excluded from the corresponding outcome synthesis. Extracted data were cross-checked by the two reviewers. Disagreements were resolved through discussion, with arbitration by a third reviewer when necessary.

### 2.4 Risk of bias and certainty of evidence assessment

The risk of bias in the included RCTs was assessed using the Cochrane Risk of Bias 2 (RoB 2) tool. The assessment domains included bias arising from the randomization process, deviations from intended interventions, missing outcome data, measurement of the outcome, and selection of the reported result. The overall risk of bias for each study was judged as low risk, some concerns, or high risk (Sterne et al., 2019). Two reviewers independently performed the assessment and cross-checked the results. Disagreements were resolved through discussion, with arbitration by a third reviewer when necessary.

The Confidence in Network Meta-Analysis (CINeMA) approach was used to assess the certainty of evidence for the main network comparisons between each intervention and UR for the FMA-UE outcome. The assessment domains included within-study bias, reporting bias, indirectness, imprecision, heterogeneity, and incoherence. The final certainty of evidence was rated as high, moderate, low, or very low (Nikolakopoulou et al., 2020).

### 2.5 Statistical analysis

Continuous outcomes were summarized as weighted mean differences (WMDs) with 95% confidence intervals (CIs). Positive values indicated greater improvement in upper-limb motor function with the corresponding intervention than with the comparator. For comparisons with direct evidence, pairwise meta-analyses were performed. When intervention nodes formed a connected network, direct and indirect evidence was synthesized using a frequentist random-effects network meta-analysis. Given the clinical differences across the included studies in stroke stage, baseline upper-limb function, training dose, treatment duration, and type of control intervention, a random-effects model was used for the primary analysis. All quantitative analyses were performed using Stata 16.0.

For multi-arm studies, all eligible study arms were retained in the network meta-analysis, and within-study correlations were accounted for in the model. Network plots were used to show direct comparisons between intervention nodes. The transitivity assumption was assessed descriptively by comparing the distribution of potential effect modifiers across direct comparisons, including stroke stage, baseline function, intervention dose, treatment duration, and type of control intervention. Network consistency was assessed using the loop-specific approach, node-splitting approach, and global inconsistency test (Dias et al., 2013; Shim et al., 2017). For networks without closed loops, local inconsistency was not assessed. If no significant inconsistency was detected, the main results were interpreted based on the consistency model.

Treatment ranking was assessed using the surface under the cumulative ranking curve (SUCRA) (Chaimani et al., 2013; Shim et al., 2017). Rankings were interpreted together with effect estimates, 95% CIs, network structure, and certainty of evidence. Exploratory subgroup analyses were performed according to stroke stage. The subacute stage was defined as 7 days to 6 months after stroke onset, and the chronic stage was defined as more than 6 months after onset. Studies that enrolled patients across different stroke stages and did not provide separately extractable data were classified as mixed-stage studies and were excluded from the stage-specific subgroup analyses. Sensitivity analyses of the FMA-UE network meta-analysis were performed by excluding mixed-stage studies and by using alternative pre–post correlation coefficients of *r* = 0.25 and *r* = 0.75. For the primary outcome, FMA-UE, comparison-adjusted funnel plots were used to assess small-study effects and potential publication bias (Chaimani and Salanti, 2012; Chaimani et al., 2013).

## 3 Results

### 3.1 Study selection and study characteristics

The database search identified 674 records: 63 from PubMed, 181 from Embase, 142 from Web of Science, 140 from the Cochrane Library, and 148 from Scopus. After 349 duplicates were removed, 325 records were screened by title and abstract. Of these, 295 records were excluded during title and abstract screening, and 30 full-text articles were assessed for eligibility. Eight articles were excluded at the full-text stage: four did not report the relevant primary outcome, three did not provide extractable outcome data, and one was a duplicate publication, an extended report from the same cohort, or a follow-up report. Finally, 22 RCTs were included in the systematic review and network meta-analysis. The study selection process is shown in Figure 1.

**Figure 1:**
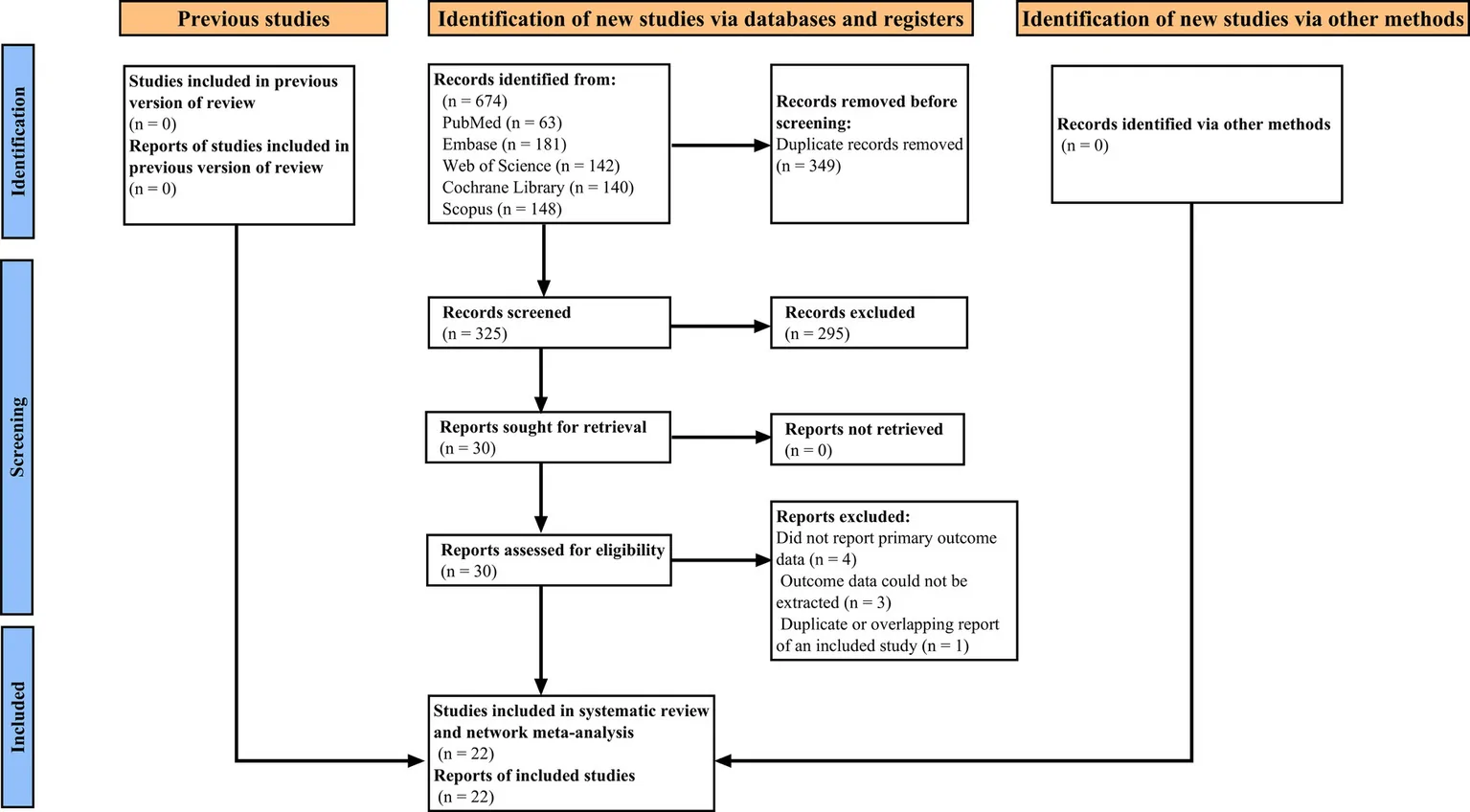
PRISMA flow diagram of study selection.

This review included 22 parallel-group RCTs (Mihara et al., 2013; Ramos-Murguialday et al., 2013; Ang et al., 2014; Ang et al., 2015; Pichiorri et al., 2015; Frolov et al., 2017; Wu et al., 2019; Chen et al., 2020; Cheng et al., 2020; Miao et al., 2020; Hu et al., 2021; Guo et al., 2022; Li et al., 2022; Fu et al., 2023; Liu et al., 2023; Ma et al., 2023; Wang et al., 2024; Cantillo-Negrete et al., 2025; He et al., 2025; Ji et al., 2025; Kim et al., 2025; Lu et al., 2025), involving 956 patients with post-stroke upper-limb motor impairment. The studies were published from 2013 to 2025. The included populations comprised patients in the subacute stage, chronic stage, or mixed post-stroke stages. Studies differed in time since stroke, baseline upper-limb motor function, BCI feedback modality, control intervention, and training dose. BCI training was usually delivered for 2–6 weeks, with 3–5 sessions per week and sessions lasting approximately 20–120 min. All included studies reported the FMA-UE, and some also reported the ARAT or the WMFT. Detailed characteristics of the included studies are provided in Appendix E4.1.

### 3.2 Risk of bias, certainty of evidence, and small-study effects

Risk of bias in the included studies was assessed using the RoB 2 tool. Overall, one study was judged to be at low risk of bias, representing 4.5% of the included studies. The remaining 21 studies were judged to have some concerns, representing 95.5%. No study was judged to be at high overall risk of bias. The main sources of bias were related to the randomization process, deviations from intended interventions, and selection of the reported result. Figure 2 shows the overall distribution of risk of bias. Figure 3 shows the domain-level risk-of-bias judgments for each study. Supporting reasons are provided in Appendix E5.

**Figure 2:**
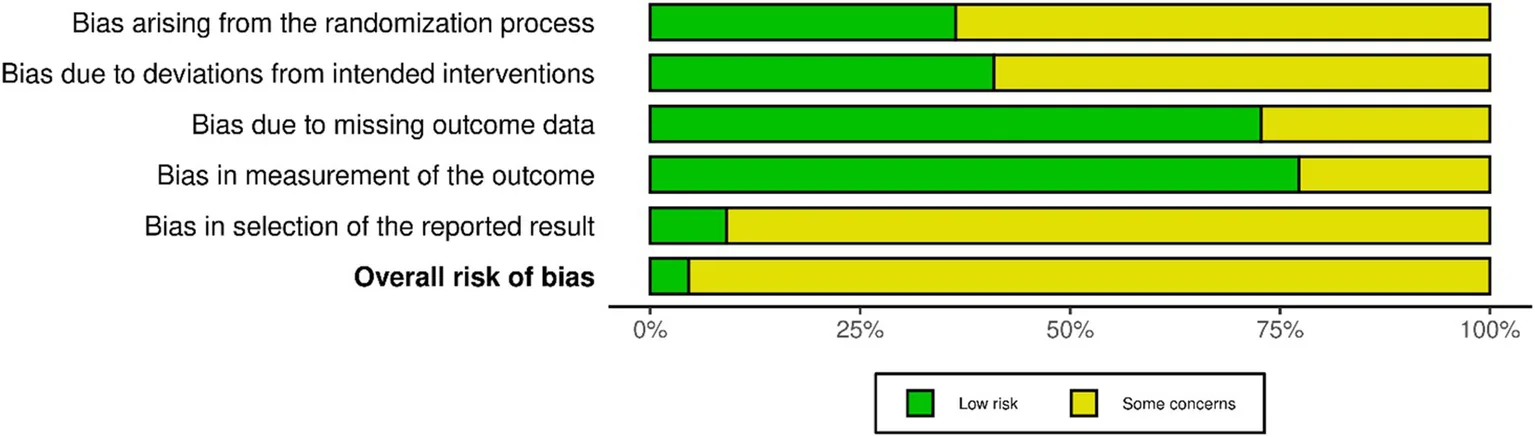
Summary of risk-of-bias judgments for the primary outcome.

**Figure 3:**
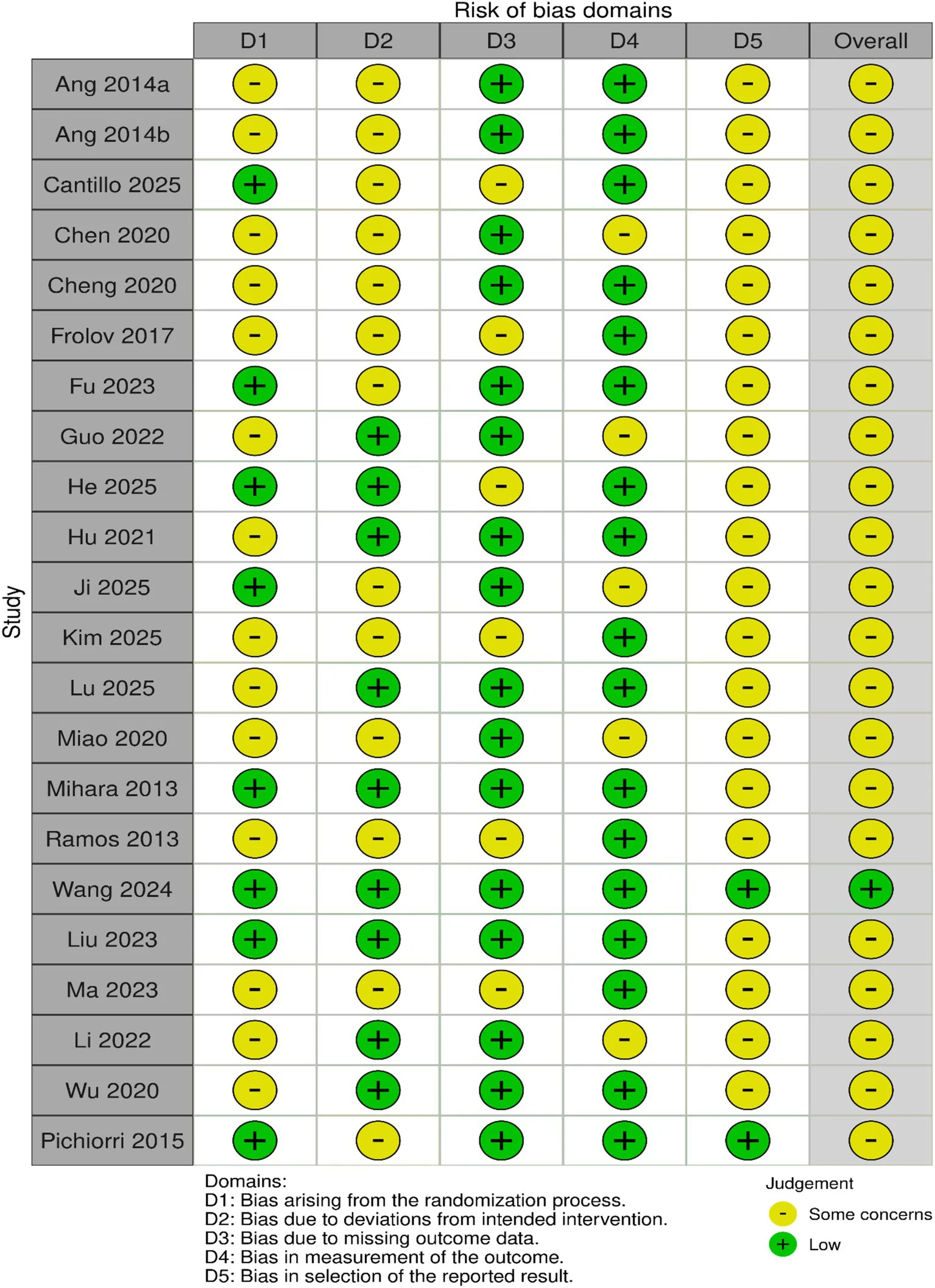
Risk-of-bias judgments for the primary outcome in individual studies.

The CINeMA assessment showed that the certainty of evidence for comparisons between each intervention and UR for the FMA-UE outcome was generally low to very low. The certainty of evidence for BCI-MM, BCI-Robot, and Robot versus UR was low, mainly because of downgrading for within-study bias and heterogeneity. The certainty of evidence for the remaining comparisons was very low, mainly due to within-study bias, imprecision, heterogeneity, or incoherence. The complete CINeMA assessment is provided in Appendix E5.

The comparison-adjusted funnel plot did not show marked asymmetry. No clear evidence of small-study effects or publication bias was observed (Appendix E5).

### 3.3 Network meta-analysis for FMA-UE

Twenty-two studies were included in the primary network meta-analysis for FMA-UE, forming 11 intervention nodes (Mihara et al., 2013; Ramos-Murguialday et al., 2013; Ang et al., 2014; Ang et al., 2015; Pichiorri et al., 2015; Frolov et al., 2017; Wu et al., 2019; Chen et al., 2020; Cheng et al., 2020; Miao et al., 2020; Hu et al., 2021; Guo et al., 2022; Li et al., 2022; Fu et al., 2023; Liu et al., 2023; Ma et al., 2023; Wang et al., 2024; Cantillo-Negrete et al., 2025; He et al., 2025; Ji et al., 2025; Kim et al., 2025; Lu et al., 2025). All interventions were connected within a single network, with BCI-MM, BCI-Robot, and UR as the main evidence nodes (Figure 4). The transitivity assessment showed some differences in participant and intervention characteristics across direct comparisons. These differences mainly involved stroke stage, baseline FMA-UE score, training dose, treatment duration, and type of control intervention. No clear systematic imbalance was observed (Appendices E3 and E4). No significant local or global inconsistency was detected in the FMA-UE network. The results of pairwise meta-analyses were generally consistent in direction with those of the network meta-analysis. Therefore, the primary analysis was interpreted using a random-effects consistency model (Appendix E6).

**Figure 4:**
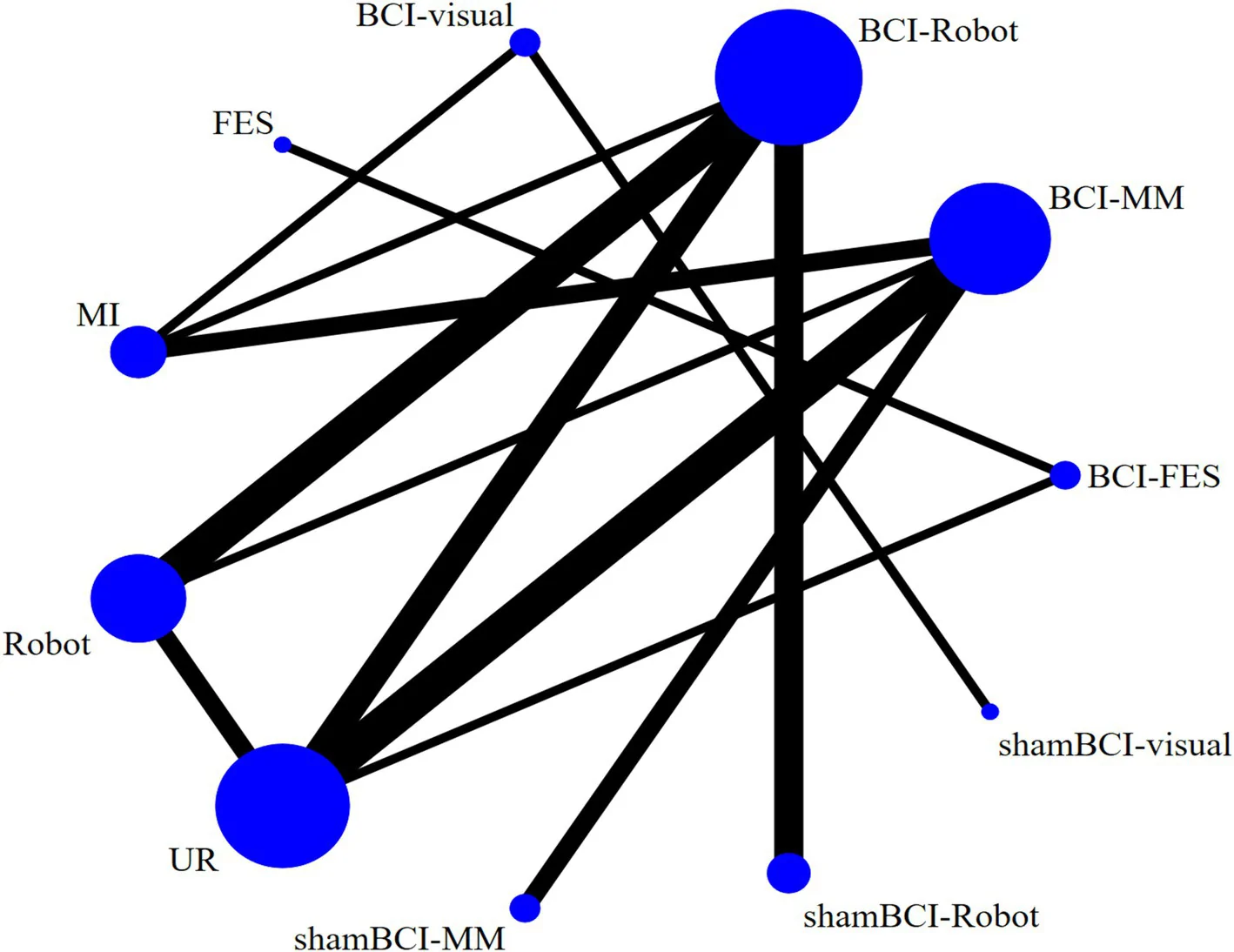
Network plot for FMA-UE.

In the random-effects consistency model, BCI-Robot was associated with significantly greater improvement in FMA-UE scores than UR (WMD = 6.28, 95% CI: 2.70–9.86, *p* = 0.001). Robot and BCI-MM were also associated with significantly greater improvement than UR, with effect estimates of WMD = 4.26 (95% CI: 0.42–8.10, *p* = 0.030) and WMD = 4.03 (95% CI: 1.00–7.06, *p* = 0.009), respectively (Figure 5; Table 1). No statistically significant differences were observed for the remaining interventions compared with UR.

**Figure 5:**
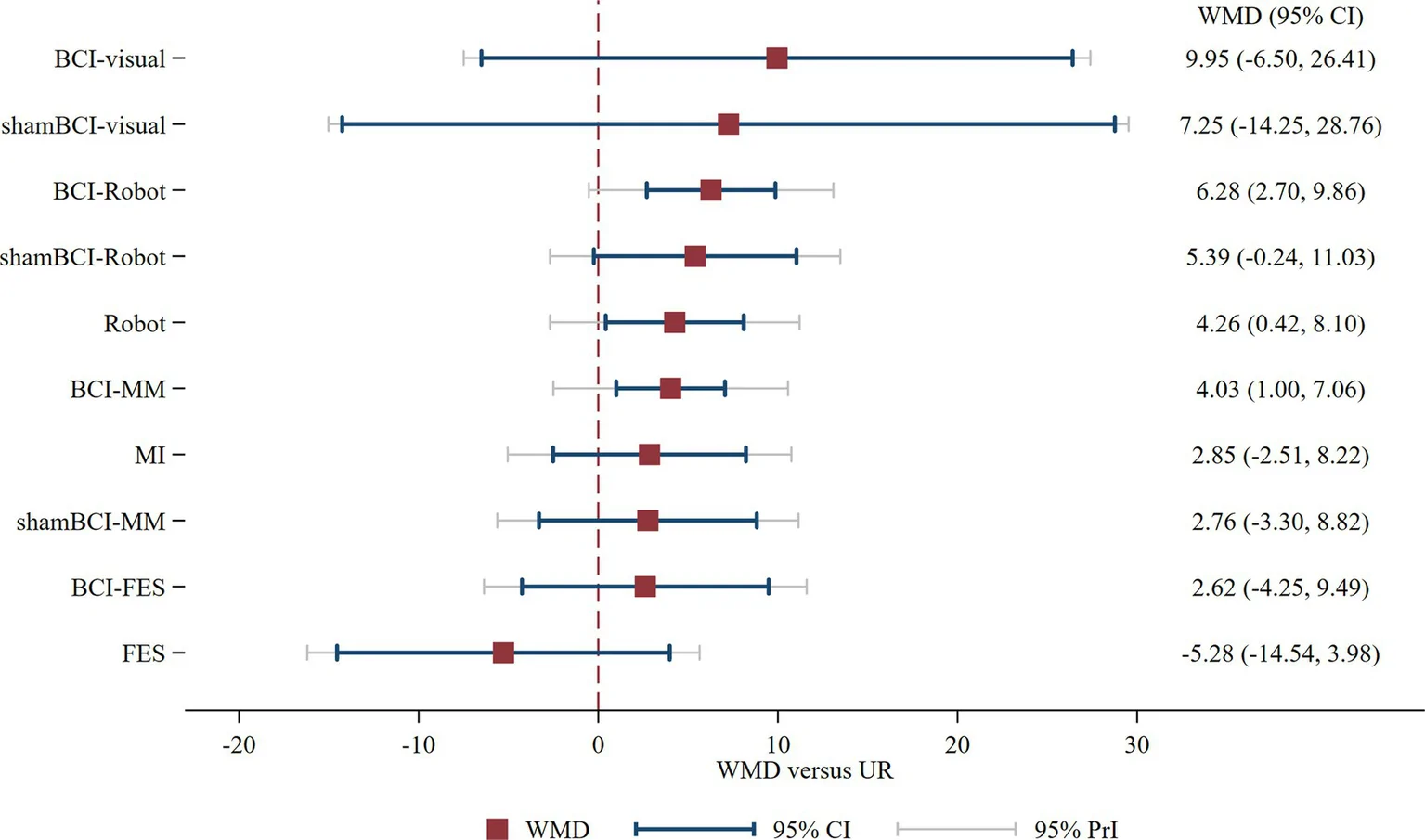
Forest plot of network meta-analysis estimates compared with UR.

**Table 1:** Network estimates and treatment rankings for FMA-UE compared with UR.

Intervention	WMD vs UR (95% CI)	SUCRA (%)	PrBest (%)	Certainty
BCI-visual	9.95 (−6.50–26.41)	77.6	39.4	Very low
BCI-robot	6.28 (2.70–9.86)	76.3	11.9	Low
shamBCI-robot	5.39 (−0.24–11.03)	64.7	9.6	Very low
shamBCI-visual	7.25 (−14.25–28.76)	61.1	27.1	Very low
Robot	4.26 (0.42–8.10)	56.6	2.7	Low
BCI-MM	4.03 (1.00–7.06)	55.5	1.5	Low
BCI-FES	2.62 (−4.25–9.49)	45.3	4.5	Very low
shamBCI-MM	2.76 (−3.30–8.82)	45.0	3.0	Very low
MI	2.85 (−2.51–8.22)	43.0	0.3	Very low
UR	Reference	18.4	0.0	—
FES	−5.28 (−14.54–3.98)	6.4	0.0	Very low

WMD > 0 indicates that the corresponding intervention was more favorable than UR.

Pairwise comparisons between BCI feedback modalities showed no statistically significant differences. The ranking results showed that BCI-visual had the highest SUCRA value, at 77.6%, followed by BCI-Robot at 76.3% (Figure 6; Table 1). However, the effect estimate for BCI-visual versus UR did not reach statistical significance, the confidence interval was wide, and the certainty of evidence was very low. BCI-Robot was associated with a statistically significant improvement versus UR, and the certainty of evidence for this comparison was low. The complete pairwise comparisons between interventions are provided in Appendix E6.

**Figure 6:**
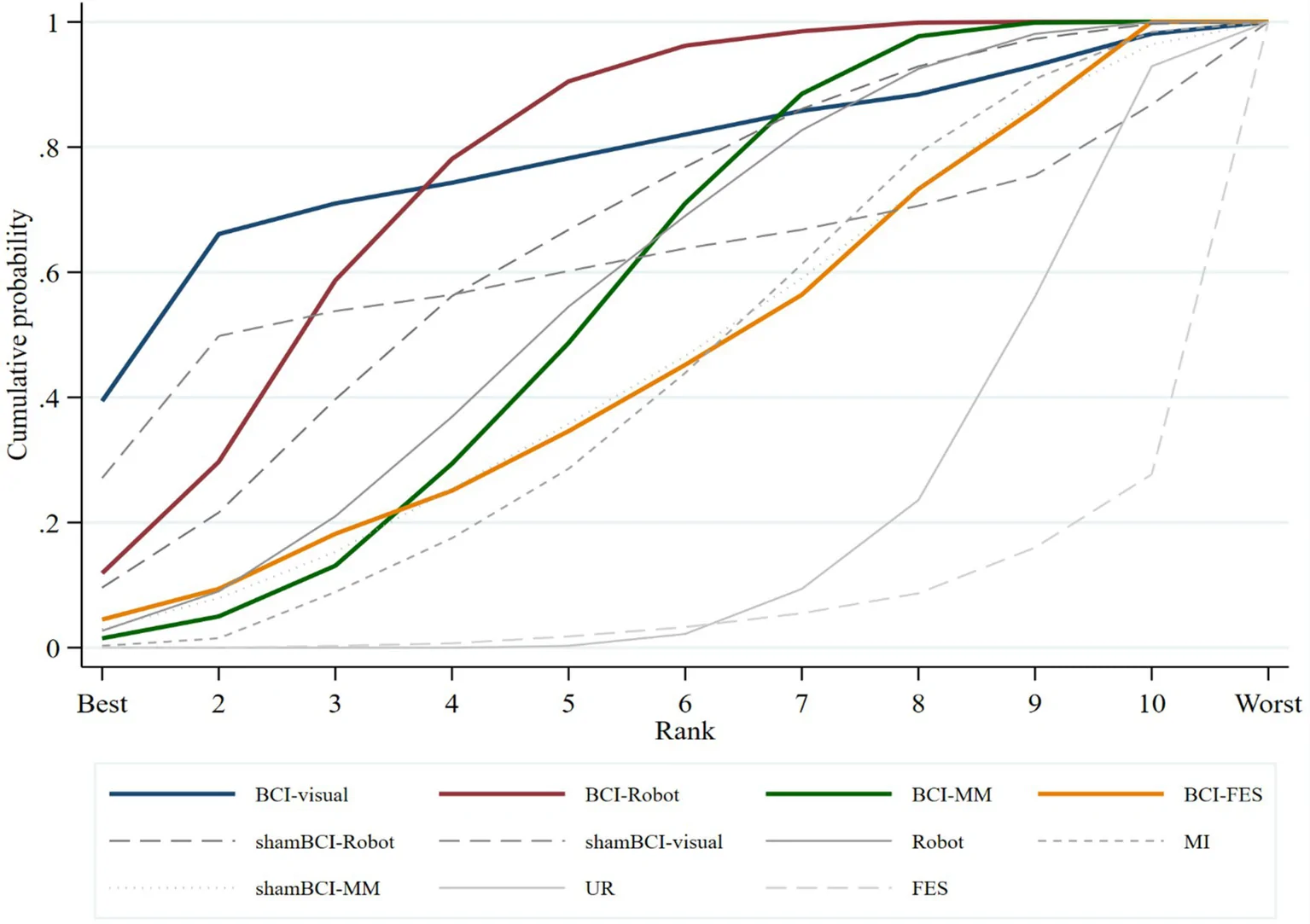
Cumulative ranking probability curves for FMA-UE.

### 3.4 Secondary outcomes

The network meta-analysis for ARAT included eight studies with 514 patients (Frolov et al., 2017; Wu et al., 2019; Cheng et al., 2020; Hu et al., 2021; Wang et al., 2024; Cantillo-Negrete et al., 2025; Ji et al., 2025; Lu et al., 2025). In the random-effects consistency model, BCI-Robot was associated with significantly greater improvement in ARAT scores than UR (WMD = 11.99, 95% CI: 1.34–22.65, *p* = 0.027). BCI-MM showed a trend toward greater improvement compared with UR, but the difference did not reach statistical significance (WMD = 4.69, 95% CI: −0.26–9.65, *p* = 0.063). No statistically significant differences were observed for the remaining interventions compared with UR. Treatment ranking showed that BCI-Robot had the highest SUCRA value (91.3%). Supplementary network analysis results for ARAT are provided in Appendix E7.

The WMFT analysis included five studies with 435 patients (Wu et al., 2019; Guo et al., 2022; Li et al., 2022; Liu et al., 2023; Wang et al., 2024). Given the small number of included studies and the sparse evidence network, pairwise meta-analysis of BCI-MM versus UR was used as the main quantitative analysis. Network meta-analysis of the largest connected network was conducted as an exploratory analysis. Pairwise meta-analysis showed that BCI-MM tended to improve WMFT scores compared with UR, but the difference did not reach statistical significance (WMD = 5.07, 95% CI: −0.39–10.53, *p* = 0.069). The exploratory network meta-analysis showed a consistent direction of effect, with an effect estimate of WMD = 5.07 for BCI-MM versus UR (95% CI: −0.48–10.62, *p* = 0.074). Supplementary analyses for WMFT are provided in Appendix E8.

Findings for the secondary outcomes were generally consistent with the primary analysis. For ARAT, BCI-Robot was associated with significantly greater improvement than UR. For WMFT, BCI-MM showed a trend toward improvement compared with UR. Given the limited number of studies for the secondary outcomes and the sparse evidence for some comparisons, these findings should be interpreted as supportive evidence for the primary analysis.

### 3.5 Subgroup analysis by stroke stage

Exploratory subgroup analyses were performed according to stroke stage. After one study that was not connected to the main network was excluded, the subacute-stage subgroup included eight studies with 531 patients (Mihara et al., 2013; Pichiorri et al., 2015; Wu et al., 2019; Chen et al., 2020; Fu et al., 2023; Wang et al., 2024; He et al., 2025; Ji et al., 2025). With UR as the reference, only BCI-MM was associated with significantly greater improvement in FMA-UE scores (WMD = 6.00, 95% CI: 0.88–11.13, *p* = 0.022). BCI-Robot showed a trend toward greater improvement, but the difference did not reach statistical significance (WMD = 5.25, 95% CI: −2.33–12.83, *p* = 0.174). The chronic-stage subgroup included eight studies with 196 patients (Ramos-Murguialday et al., 2013; Ang et al., 2014; Ang et al., 2015; Cheng et al., 2020; Miao et al., 2020; Guo et al., 2022; Kim et al., 2025; Lu et al., 2025). BCI-Robot (WMD = 5.42, 95% CI: 0.81–10.03, *p* = 0.021) and Robot (WMD = 4.86, 95% CI: 0.41–9.30, *p* = 0.032) were associated with significantly greater improvement than UR. The complete subgroup analysis results are provided in Appendix E9.

### 3.6 Sensitivity analysis

After studies with mixed stroke stages were excluded, 17 studies remained, and the evidence network remained connected. Compared with UR, BCI-Robot (WMD = 6.35, 95% CI: 3.08–9.62, *p* < 0.001), BCI-MM (WMD = 5.28, 95% CI: 1.93–8.62, *p* = 0.002), and Robot (WMD = 4.60, 95% CI: 1.06–8.14, *p* = 0.011) remained associated with significantly greater improvement. The direction of effect was consistent with the primary analysis, suggesting that the primary findings were relatively robust (Appendix E10). Sensitivity analyses using *r* = 0.25 and *r* = 0.75 yielded network estimates and treatment rankings consistent with the primary analysis, without changing the overall interpretation (Appendix E11).

## 4 Discussion

### 4.1 Principal findings

This systematic review and network meta-analysis compared the relative efficacy of BCI training with different feedback modalities for post-stroke upper-limb motor impairment. Based on evidence from 22 RCTs involving 956 patients, BCI-Robot, BCI-MM, and Robot were associated with significantly greater improvement in FMA-UE scores than UR. Among these statistically significant comparisons, BCI-Robot had the largest effect estimate. Findings for the secondary outcomes were generally consistent with the primary analysis. For ARAT, BCI-Robot was associated with significantly greater improvement than UR. For WMFT, BCI-MM showed a trend toward improvement compared with UR, but the difference did not reach statistical significance. Subgroup analysis by stroke stage suggested that the relative performance of different feedback modalities may vary according to time since stroke. In the subacute-stage subgroup, BCI-MM showed a clearer improvement relative to UR, whereas BCI-Robot and Robot showed more consistent improvement in the chronic-stage subgroup. The sensitivity analysis excluding studies with mixed stroke stages showed that BCI-Robot, BCI-MM, and Robot remained associated with significantly greater improvement than UR. This finding suggests that the primary results were relatively robust.

Taken together, BCI-Robot appears to be a reasonable priority for further evaluation among BCI feedback modalities. However, network comparisons between BCI feedback modalities did not show statistically significant differences, and the certainty of evidence for the main FMA-UE comparisons was generally low to very low.

### 4.2 Interpretation of different feedback modalities

The core feature of BCI training is not simply assistance provided by an external device. Rather, it is the establishment of a temporally coupled closed-loop process linking the patient’s motor intention, external feedback, and sensory reafference (Pichiorri et al., 2015; Cervera et al., 2018). Post-stroke motor recovery is closely related to neuroplasticity, reorganization of the sensorimotor system, and task-related training (Dimyan and Cohen, 2011; Takeuchi and Izumi, 2013). By decoding brain activity associated with MI, BCI can allow patients with insufficient voluntary motor capacity to receive feedback contingent on their motor intention. This process may enhance sensorimotor rhythm modulation and experience-dependent plasticity (Pichiorri et al., 2015; Cervera et al., 2018). Therefore, in post-stroke upper-limb rehabilitation, the clinical effects of BCI may depend not only on the BCI technology itself but also on the type of therapeutic feedback triggered by the decoded signals.

The observed effects of BCI-Robot may be related to its integration of motor intention recognition with robot-assisted movement feedback. BCI can decode brain activity associated with MI or attempted movement in real time and drive a robot, exoskeleton, or an orthosis to generate upper-limb assisted movement that is matched to the patient’s motor intention. This process forms a closed-loop training pathway that links motor intention, assisted movement, and sensory reafference. Compared with MI alone or passive assistance unrelated to motor intention, this feedback modality may more effectively strengthen the coupling between motor intention, assisted movement, and sensory input. Previous studies of BCI-controlled robots and hand exoskeletons have also suggested that robotic or exoskeletal feedback controlled by BCI may promote post-stroke upper-limb motor recovery (Ramos-Murguialday et al., 2013; Ang et al., 2015; Frolov et al., 2017). In this study, BCI-Robot was associated with statistically significant improvements versus UR in both FMA-UE and ARAT outcomes, while the certainty of evidence for the FMA-UE comparison was low. For FMA-UE, BCI-Robot showed a 6.28-point greater improvement than UR, a magnitude comparable to clinically important differences reported in patients with chronic stroke and minimal-to-moderate upper-limb impairment (Page et al., 2012), suggesting potential clinical relevance. However, the clinical relevance of this effect may vary across patient populations, given the differences in stroke stage and baseline impairment among the included studies.

The treatment effect of BCI-Robot, however, cannot yet be attributed solely to BCI closed-loop control. In this study, the Robot node was also associated with significantly greater improvement than UR. This suggests that high-repetition, task-oriented motor practice and proprioceptive input provided by robot-assisted training may already constitute important therapeutic components. Current evidence is therefore insufficient to determine whether BCI closed-loop control provides additional benefit over non-BCI robot training. BCI-Robot should be interpreted as a combined rehabilitation approach that integrates motor intention recognition with robot-assisted movement, rather than as evidence for the effect of BCI as a single component.

BCI-MM also showed potential clinical benefit. Multimodal feedback can integrate several feedback components, including visual feedback, FES, robotic or soft external effectors, tactile feedback, and proprioceptive input. This integration may enhance patient engagement with motor tasks, increase feedback salience, and support sensorimotor integration (Cheng et al., 2020; Hu et al., 2021; Wang et al., 2024; He et al., 2025; Kim et al., 2025; Lu et al., 2025). Previous clinical studies have suggested that motor imagery-related contingent feedback, multisensory feedback, and BCI training combined with external effectors may be associated with improvements in upper-limb function (Cheng et al., 2020; Hu et al., 2021; Wang et al., 2024; He et al., 2025; Kim et al., 2025; Lu et al., 2025). Some studies also reported changes in functional connectivity, regional brain activation, or neuroplasticity-related measures (He et al., 2025; Kim et al., 2025; Lu et al., 2025). In this study, BCI-MM was associated with significantly greater improvement than UR in the primary analysis and in the sensitivity analysis excluding mixed-stage studies. It was also associated with greater improvement in the subacute-stage subgroup. These findings suggest that multimodal closed-loop feedback may warrant further evaluation in future research. However, BCI-MM should be understood as a category of combined feedback strategies rather than as a single homogeneous intervention. Its effects may be related to enhanced sensorimotor integration through multi-channel feedback. At present, it remains unclear which component, such as visual feedback, FES, robotics, virtual reality, tactile feedback, or proprioceptive input, contributes most.

Some studies used sham or non-contingent BCI control conditions, and the corresponding sham BCI nodes were retained in the network. The results suggested that BCI-MM showed greater improvement than sham BCI-MM. In contrast, comparisons of BCI-Robot versus sham BCI-Robot and BCI-visual versus sham BCI-visual showed no clear differences. These findings suggest that the added value of contingent closed-loop feedback may depend on the feedback modality. BCI-FES and BCI-visual also did not show consistent benefits in this study. Mechanistically, BCI-FES can couple motor intention with target muscle contraction and peripheral sensory input, thereby forming a central-peripheral closed-loop feedback mechanism (Ren et al., 2024). However, the evidence was limited mainly by the small number of studies, imprecise effect estimates, and low certainty of evidence. Therefore, treatment priorities should not be determined on the basis of ranking results alone.

### 4.3 Clinical implications by stroke stage

Stroke stage may influence the effects of different BCI feedback modalities. Post-stroke motor recovery is time-dependent, with spontaneous recovery and training-induced plasticity contributing more prominently during the subacute stage, whereas the contribution of spontaneous recovery is generally reduced in the chronic stage. Nevertheless, further improvement may still be achieved through high-intensity, task-related, and feedback-enhanced rehabilitation training (Hatem et al., 2016; Ballester et al., 2019; Dromerick et al., 2021). In this study, only BCI-MM was associated with significantly greater improvement in FMA-UE scores than UR in the subacute-stage subgroup. In the chronic-stage subgroup, BCI-Robot and Robot were associated with significantly greater improvement than UR. The sensitivity analysis excluding mixed-stage studies showed that BCI-Robot, BCI-MM, and Robot remained associated with significantly greater improvement than UR, suggesting that the primary findings were unlikely to be driven solely by mixed-stage studies.

These findings suggest possible stage-related differences in the performance of BCI feedback modalities. Multimodal sensory feedback may be relevant during the subacute stage, whereas stable, repetitive, and task-oriented motor execution feedback may be relevant during the chronic stage. However, these subgroup analyses were exploratory. The number of studies in each stage was limited, and differences in baseline function, training dose, and control interventions remained. Therefore, the current evidence is insufficient to support definitive selection of BCI feedback modalities according to stroke stage.

### 4.4 Comparison with previous evidence

Previous systematic reviews and pairwise meta-analyses have generally supported the potential benefit of BCI training for post-stroke upper-limb motor function (Cervera et al., 2018). A meta-analysis including more RCTs showed that BCI-based training was associated with improvements in FMA-UE, WMFT, and ARAT, and suggested that stroke stage, type of external feedback, and training intensity may influence treatment effects (Li et al., 2025). However, previous studies mainly used pairwise meta-analysis or subgroup analysis and primarily evaluated the overall effect of BCI training compared with control interventions. The relative efficacy of different BCI feedback modalities has not been systematically assessed within a common comparative framework.

Evidence has also accumulated for specific BCI feedback modalities. A systematic review of BCI-Robot showed that BCI-Robot systems may improve post-stroke upper-limb motor function, but found no clear statistically significant difference between BCI-Robot and Robot alone (Qu et al., 2024). A meta-analysis of BCI-FES suggested that BCI-controlled FES may promote upper-limb functional recovery, although its scope was limited to a single feedback modality (Ren et al., 2024). In contrast, the present study constructed network nodes according to the primary therapeutic feedback modality and contingent closed-loop characteristics. This approach allowed comparison of BCI-Robot, BCI-MM, BCI-FES, BCI-visual, and the corresponding control interventions, and evaluation of the relative efficacy of different BCI feedback modalities within a single network framework.

### 4.5 Strengths and limitations

A major strength of this study is that it compared BCI-based upper-limb rehabilitation interventions according to feedback modality, rather than pooling BCI training as a homogeneous intervention. Intervention nodes were classified according to the primary therapeutic feedback modality and contingent closed-loop characteristics, allowing BCI-MM, BCI-Robot, BCI-FES, BCI-visual, and the corresponding control nodes to be compared within a single network framework. This approach improved the clinical interpretability of comparisons across complex BCI interventions. For the primary outcome, the FMA-UE network was connected, and no significant global or local inconsistency was detected. Certainty-of-evidence assessment, subgroup analysis, and sensitivity analysis were used to evaluate the credibility and robustness of the main findings.

This study also has several limitations. Although no clear systematic imbalance was identified, differences in stroke stage, baseline FMA-UE score, training dose, treatment duration, and comparator characteristics should be considered when judging the plausibility of transitivity and interpreting indirect estimates. The BCI-Robot node included heterogeneous robotic devices, exoskeletal systems, and BCI configurations, which may challenge the transitivity assumption for comparisons involving this node. The exploratory stage-specific findings suggest that stroke stage may be relevant to relative treatment effects, while the absence of statistically significant local or global inconsistency does not demonstrate that the transitivity assumption holds. In addition, the overall sample size was limited, some nodes were supported by only a small number of studies, and direct comparisons between BCI feedback modalities were insufficient, which may reduce the stability of network estimates and increase reliance on indirect evidence. The certainty of evidence for the main FMA-UE comparisons was generally low to very low. The interpretation of SUCRA rankings is also limited because higher rankings may not indicate clinical superiority when the corresponding effect estimates are statistically non-significant, imprecise, or supported by low or very low certainty evidence. Some BCI interventions included multiple feedback components; therefore, node classification and data conversion for some outcomes may have introduced additional uncertainty. Evidence for ARAT, WMFT, long-term follow-up, safety, quality of life, and activities of daily living also remains insufficient. Finally, the review was restricted to English-language, peer-reviewed full-text articles and did not search clinical trial registries or gray literature, which may increase the risk of retrieval and publication bias. Future studies should conduct large, standardized, direct-comparison RCTs to further clarify the relative efficacy of different BCI feedback modalities and identify the patient populations most likely to benefit.

## 5 Conclusion

This study suggests that BCI training with different feedback modalities may have different effects on post-stroke upper-limb motor recovery. For FMA-UE, BCI-Robot and BCI-MM were associated with statistically significant improvements compared with UR; however, the certainty of evidence for both comparisons was low. BCI-Robot was also associated with a statistically significant improvement in ARAT compared with UR. Direct comparative evidence among BCI feedback modalities remains insufficient, and there is no clear evidence that any single feedback modality is statistically superior to the others. Given the small sample size for some nodes and the generally low certainty of evidence, the current findings are more appropriate for identifying research priorities and informing BCI-based rehabilitation program design.

## Data Availability

The original contributions presented in the study are included in the article/Supplementary material, further inquiries can be directed to the corresponding author/s.
