# Supplementary material for: Comparative efficacy of brain-computer interface training with different feedback modalities for post-stroke upper-limb motor recovery: a systematic review and network meta-analysis of randomized controlled trials
Source: Front Neurosci. 2026 Oct 16;20:1925602. doi: 10.3389/fnins.2026.1925602 (PMC13719565; doi:10.3389/fnins.2026.1925602)
Supplement: Supplementary file 1 [file Data_Sheet_1.PDF]

Supplementary Appendix

Comparative efficacy of brain-computer interface training with different feedback modalities for post-stroke upper-limb motor recovery: a systematic review and network meta-analysis of randomized controlled trials

Table of contents

Appendix E1: PRISMA-NMA checklist 1

Appendix E2: Search strategies 5

Appendix E3: Intervention node definitions and classification 9

Appendix E4: Characteristics of included studies and data conversion methods 15

Appendix E5: Risk of bias, certainty of evidence, and small-study effects 19

Appendix E6: Supplementary analyses for the primary outcome FMA-UE ……..24

Appendix E7: Supplementary analyses for the ARAT outcome ……………...27

Appendix E8: Supplementary analyses for the WMFT outcome………………………………………. 28

Appendix E9: Subgroup analysis by stroke stage..................... 29

Appendix E10: Sensitivity analysis excluding studies with mixed stroke stages………………………..............33

Appendix E11: Sensitivity analyses using alternative pre–post correlation coefficients 35

Appendix E12: References and analysis inclusion status of included studies ……………..............................36

Appendix E1: PRISMA-NMA Checklist

Table E1.1. PRISMA-NMA checklist

Section/Topic	Item #	Checklist Item	Reported on Page #
**TITLE**			
Title	1	Identify the report as a systematic review *incorporating a network meta-analysis (or related form of meta-analysis).*	P1 (Title)
**ABSTRACT**			
Structured summary	2	Provide a structured summary including, as applicable:<br>**Background:** main objectives<br>**Methods:** data sources; study eligibility criteria, participants, and interventions; study appraisal; and *synthesis methods, such as network meta-analysis.*<br>**Results:** number of studies and participants identified; summary estimates with corresponding confidence/credible intervals; *treatment rankings may also be discussed. Authors may choose to summarize pairwise comparisons against a chosen treatment included in their analyses for brevity.*<br>**Discussion/Conclusions:** limitations; conclusions and implications of findings.<br>**Other:** primary source of funding; systematic review registration number with registry name.	P2 (Abstract)
**INTRODUCTION**			
Rationale	3	Describe the rationale for the review in the context of what is already known*, including mention of why a network meta-analysis has been conducted.*_	P3–5 (Introduction)
Objectives	4	Provide an explicit statement of questions being addressed, with reference to participants, interventions, comparisons, outcomes, and study design (PICOS).	P4–5 (Introduction)
**METHODS**			
Protocol and registration	5	Indicate whether a review protocol exists and if and where it can be accessed (e.g., Web address); and, if available, provide registration information, including registration number.	P5 (Methods)
Eligibility criteria	6	Specify study characteristics (e.g., PICOS, length of follow-up) and report characteristics (e.g., years considered, language, publication status) used as criteria for eligibility, giving rationale. *Clearly describe eligible treatments included in the treatment network, and note whether any have been clustered or merged into the same node (with justification).*	P5–P6 (Methods, Eligibility criteria and intervention node classification)
Information sources	7	Describe all information sources (e.g., databases with dates of coverage, contact with study authors to identify additional studies) in the search and date last searched.	P6-P7 (Methods, Search strategy and study selection)
Search	8	Present full electronic search strategy for at least one database, including any limits used, such that it could be repeated.	P6-P7 (Methods, Search strategy and study selection); Appendix E2

Study selection	9	State the process for selecting studies (i.e., screening, eligibility, included in systematic review, and, if applicable, included in the meta-analysis).	P6-7 (Methods, Search strategy and study selection); Figure 1
Data collection process	10	Describe method of data extraction from reports (e.g., piloted forms, independently, in duplicate) and any processes for obtaining and confirming data from investigators.	P7 (Methods, Data extraction)
Data items	11	List and define all variables for which data were sought (e.g., PICOS, funding sources) and any assumptions and simplifications made.	P5–P7 (Methods, Eligibility criteria and intervention node classification; Data extraction); Appendix E4.2
**Geometry of the network**	**S1**	Describe methods used to explore the geometry of the treatment network under study and potential biases related to it. This should include how the evidence base has been graphically summarized for presentation, and what characteristics were compiled and used to describe the evidence base to readers.	P8 (Methods, Statistical analysis)
Risk of bias within individual studies	12	Describe methods used for assessing risk of bias of individual studies (including specification of whether this was done at the study or outcome level), and how this information is to be used in any data synthesis.	P7–P8 (Methods, Risk of bias and certainty of evidence assessment)
Summary measures	13	State the principal summary measures (e.g., risk ratio, difference in means). *Also describe the use of additional summary measures assessed, such as treatment rankings and surface under the cumulative ranking curve (SUCRA) values, as well as modified approaches used to present summary findings from meta-analyses.*	P8 (Methods, Statistical analysis)
Planned methods of analysis	14	Describe the methods of handling data and combining results of studies for each network meta-analysis. This should include, but not be limited to:<br>• *Handling of multi-arm trials;*<br>• *Selection of variance structure;*<br>• *Selection of prior distributions in Bayesian analyses; and*<br>*Assessment of model fit.*	P7–P8 (Methods, Data extraction; Statistical analysis); Appendix E4.2
**Assessment of Inconsistency**	**S2**	Describe the statistical methods used to evaluate the agreement of direct and indirect evidence in the treatment network(s) studied. Describe efforts taken to address its presence when found.	P8 (Methods, Statistical analysis)
Risk of bias across studies	15	Specify any assessment of risk of bias that may affect the cumulative evidence (e.g., publication bias, selective reporting within studies).	P7–P8 (Methods)
Additional analyses	16	Describe methods of additional analyses if done, indicating which were pre-specified. This may include, but not be limited to, the following:<br>• Sensitivity or subgroup analyses;<br>• Meta-regression analyses;<br>• *Alternative formulations of the treatment network; and*<br>• *Use of alternative prior distributions for Bayesian*	P8 (Methods, Statistical analysis)

		*analyses (if applicable).*_	
**RESULTS†**			
Study selection	17	Give numbers of studies screened, assessed for eligibility, and included in the review, with reasons for exclusions at each stage, ideally with a flow diagram.	P9 (Results, Study selection and study characteristics; Figure 1)
**Presentation of network structure**	**S3**	• Provide a network graph of the included studies to enable visualization of the geometry of the treatment network.	P13 (Results, Network meta-analysis for FMA-UE; Figure 4)
**Summary of network geometry**	**S4**	Provide a brief overview of characteristics of the treatment network. This may include commentary on the abundance of trials and randomized patients for the different interventions and pairwise comparisons in the network, gaps of evidence in the treatment network, and potential biases reflected by the network structure.	P13 (Results, Network meta-analysis for FMA-UE)
Study characteristics	18	For each study, present characteristics for which data were extracted (e.g., study size, PICOS, follow-up period) and provide the citations.	P9 (Results, Study selection and study characteristics); Appendix E4.1
Risk of bias within studies	19	Present data on risk of bias of each study and, if available, any outcome level assessment.	P10-P11 (Results, Risk of bias, certainty of evidence, and small-study effects); Appendix E5
Results of individual studies	20	For all outcomes considered (benefits or harms), present, for each study: 1) simple summary data for each intervention group, and 2) effect estimates and confidence intervals. *Modified approaches may be needed to deal with information from larger networks.*	P9–P16 (Results, Study selection and study characteristics; Network meta-analysis for FMA-UE; Secondary outcomes); Appendix E4.1; Appendix E6–E8
Synthesis of results	21	Present results of each meta-analysis done, including confidence/credible intervals. *In larger networks, authors may focus on comparisons versus a particular comparator (e.g. placebo or standard care), with full findings presented in an appendix. League tables and forest plots may be considered to summarize pairwise comparisons.* If additional summary measures were explored (such as treatment rankings), these should also be presented.	P13–P16 (Results, Network meta-analysis for FMA-UE; Secondary outcomes); Appendix E6–E8
**Exploration for inconsistency**	**S5**	Describe results from investigations of inconsistency. This may include such information as measures of model fit to compare consistency and inconsistency models, P values from statistical	P13 (Results, Network meta-analysis for FMA-

		tests, or summary of inconsistency estimates from different parts of the treatment network.	UE); Appendix E6
Risk of bias across studies	22	Present results of any assessment of risk of bias across studies for the evidence base being studied.	P10-P11 (Results, Risk of bias, certainty of evidence, and small-study effects); Appendix E5
Results of additional analyses	23	Give results of additional analyses, if done (e.g., sensitivity or subgroup analyses, meta-regression analyses*, alternative network geometries studied, alternative choice of prior distributions for Bayesian analyses,* and so forth).	P16-P17 (Results, Subgroup analysis by stroke stage; Sensitivity analysis); Appendix E9–E11
**DISCUSSION**			
Summary of evidence	24	Summarize the main findings, including the strength of evidence for each main outcome; consider their relevance to key groups (e.g., healthcare providers, users, and policy-makers).	P17–P18 (Discussion, Principal findings)
Limitations	25	Discuss limitations at study and outcome level (e.g., risk of bias), and at review level (e.g., incomplete retrieval of identified research, reporting bias). *Comment on the validity of the assumptions, such as transitivity and consistency. Comment on any concerns regarding network geometry (e.g., avoidance of certain comparisons).*	P13 (Results, Network meta-analysis for FMA-UE); P21–P22 (Strengths and limitations)
Conclusions	26	Provide a general interpretation of the results in the context of other evidence, and implications for future research.	P17–P22 (Discussion)
**FUNDING**			
Funding	27	Describe sources of funding for the systematic review and other support (e.g., supply of data); role of funders for the systematic review. This should also include information regarding whether funding has been received from manufacturers of treatments in the network and/or whether some of the authors are content experts with professional conflicts of interest that could affect use of treatments in the network.	P23-P24 (Statements and declarations, Funding; Competing interests)

Appendix E2: Search strategies

Table E2.1. Search strategy for PubMed

#	**Searches**
1	"Brain-Computer Interfaces"[Mesh]
2	((((((((((((brain-computer interface[Title/Abstract]) OR (brain-computer interfaces[Title/Abstract])) OR (BCI[Title/Abstract])) OR (brain-machine interface[Title/Abstract])) OR (brain-machine interfaces[Title/Abstract])) OR (brain machine interface[Title/Abstract])) OR (brain machine interfaces[Title/Abstract])) OR (neural interface[Title/Abstract])) OR (neural interfaces[Title/Abstract])) OR (neural interface technology[Title/Abstract])) OR (neural interface technologies[Title/Abstract])) OR (brain signal interface[Title/Abstract])) OR (brain signal interfaces[Title/Abstract])
3	#1 OR #2
4	"Stroke"[Mesh]
5	((((((((((((((((((((((((((((((((((strokes[Title/Abstract]) OR stroke[Title/Abstract])) OR (acute strokes[Title/Abstract])) OR (acute stroke[Title/Abstract])) OR (cerebrovascular accident[Title/Abstract])) OR (cerebrovascular accidents[Title/Abstract])) OR (acute cerebrovascular accident[Title/Abstract])) OR (acute cerebrovascular accidents[Title/Abstract])) OR (CVA[Title/Abstract])) OR (CVAs[Title/Abstract])) OR (apoplexy[Title/Abstract])) OR (brain attack[Title/Abstract])) OR (ischemic stroke[Title/Abstract])) OR (ischaemic stroke[Title/Abstract])) OR (hemorrhagic stroke[Title/Abstract])) OR (haemorrhagic stroke[Title/Abstract])) OR (cerebral infarction[Title/Abstract])) OR (brain infarction[Title/Abstract])) OR (cerebral ischemia[Title/Abstract])) OR (cerebral ischaemia[Title/Abstract])) OR (brain ischemia[Title/Abstract])) OR (brain ischaemia[Title/Abstract])) OR (cerebral hemorrhage[Title/Abstract])) OR (cerebral haemorrhage[Title/Abstract])) OR (brain hemorrhage[Title/Abstract])) OR (brain haemorrhage[Title/Abstract])) OR (intracerebral hemorrhage[Title/Abstract])) OR (intracerebral haemorrhage[Title/Abstract])) OR (intracranial hemorrhage[Title/Abstract])) OR (intracranial haemorrhage[Title/Abstract])) OR (subarachnoid hemorrhage[Title/Abstract])) OR (subarachnoid haemorrhage[Title/Abstract])) OR (lacunar stroke[Title/Abstract])) OR (lacunar infarction[Title/Abstract])
6	#4 OR #5
7	"Upper Extremity"[Mesh]
8	((((((upper extremity[Title/Abstract]) OR (upper limb[Title/Abstract])) OR (arm[Title/Abstract])) OR (hand[Title/Abstract])) OR (shoulder[Title/Abstract])) OR (elbow[Title/Abstract])) OR (wrist[Title/Abstract])
9	#7 OR #8
10	randomized controlled trial[Publication Type] OR controlled clinical trial[Publication Type] OR randomized[Title/Abstract] OR randomised[Title/Abstract] OR randomly[Title/Abstract] OR placebo[Title/Abstract]
11	#3 AND #6 AND #9 AND #10

Table E2.2. Search strategy for Embase

#	Searches
1	'brain computer interface'/exp OR 'brain computer interface'
2	'brain-computer interface':ab,ti OR 'brain-computer interfaces':ab,ti OR 'BCI':ab,ti OR 'brain-machine interface':ab,ti OR 'brain-machine interfaces':ab,ti OR 'brain machine interface':ab,ti OR 'brain machine interfaces':ab,ti OR 'neural interface':ab,ti OR 'neural interfaces':ab,ti OR 'neural interface technology':ab,ti OR 'neural interface technologies':ab,ti OR 'brain signal interface':ab,ti OR 'brain signal interfaces':ab,ti
3	#1 OR #2
4	'cerebrovascular accident'/exp OR 'cerebrovascular accident'
5	'strokes':ab,ti OR 'acute stroke':ab,ti OR 'acute strokes':ab,ti OR 'stroke':ab,ti OR 'cerebrovascular accidents':ab,ti OR 'acute cerebrovascular accident':ab,ti OR 'acute cerebrovascular accidents':ab,ti OR 'CVA':ab,ti OR 'CVAs':ab,ti OR 'apoplexy':ab,ti OR 'brain attack':ab,ti OR 'ischemic stroke':ab,ti
6	#4 OR #5
7	'upper limb'/exp OR 'upper limb'
8	'upper extremity':ab,ti OR 'arm':ab,ti OR 'hand':ab,ti OR 'shoulder':ab,ti OR 'elbow':ab,ti OR 'wrist':ab,ti
9	#7 OR #8
10	'randomized controlled trial'/exp OR 'randomized controlled trial'
11	'controlled clinical trial':ab,ti OR 'randomized':ab,ti OR 'placebo':ab,ti OR 'RCT':ab,ti OR 'randomised':ab,ti
12	#10 OR #11
13	#3 AND #6 AND #9 AND #12

Table E2.3. Search strategy for Web of Science

#	Searches
1	TS=(brain-computer interface OR brain-computer interfaces OR BCI OR brain-machine interface OR brain-machine interfaces OR brain machine interface OR brain machine interfaces OR neural interface OR neural interfaces OR neural interface technology OR neural interface technologies OR brain signal interface OR brain signal interfaces)
2	TS=(strokes OR acute stroke OR acute strokes OR cerebrovascular accident OR cerebrovascular accidents OR acute cerebrovascular accident OR acute cerebrovascular accidents OR CVA OR CVAs OR apoplexy OR brain attack OR ischemic stroke OR ischaemic stroke OR hemorrhagic stroke OR haemorrhagic stroke OR cerebral infarction OR brain infarction OR cerebral ischemia OR cerebral ischaemia OR brain ischemia OR brain ischaemia OR cerebral hemorrhage OR cerebral haemorrhage OR brain hemorrhage OR brain haemorrhage OR intracerebral hemorrhage OR intracerebral haemorrhage OR intracranial hemorrhage OR intracranial haemorrhage OR subarachnoid hemorrhage OR subarachnoid haemorrhage OR lacunar stroke OR lacunar infarction)

3	TS=(upper limb OR arm OR hand OR shoulder OR elbow OR wrist)
4	TS=(randomized controlled trial OR controlled clinical trial OR randomized OR placebo OR RCT OR randomised)
5	#1 AND #2 AND #3 AND #4

Table E2.4. Search strategy for Cochrane Library

#	Searches
1	MeSH descriptor: [Brain-Computer Interfaces] explode all trees
2	(brain-computer interface):ti,ab,kw OR (brain-computer interfaces):ti,ab,kw OR (BCI):ti,ab,kw OR (brain-machine interface):ti,ab,kw OR (brain-machine interfaces):ti,ab,kw OR (brain machine interface):ti,ab,kw OR (brain machine interfaces):ti,ab,kw OR (neural interface):ti,ab,kw OR (neural interfaces):ti,ab,kw OR (neural interface technology):ti,ab,kw OR (neural interface technologies):ti,ab,kw OR (brain signal interface):ti,ab,kw OR (brain signal interfaces):ti,ab,kw
3	#1 OR #2
4	MeSH descriptor: [Stroke] explode all trees
5	(stroke):ti,ab,kw OR (strokes):ti,ab,kw OR (acute stroke):ti,ab,kw OR (acute strokes):ti,ab,kw OR (cerebrovascular accident):ti,ab,kw OR (cerebrovascular accidents):ti,ab,kw OR (acute cerebrovascular accident):ti,ab,kw OR (acute cerebrovascular accidents):ti,ab,kw OR (CVA):ti,ab,kw OR (CVAs):ti,ab,kw OR (apoplexy):ti,ab,kw OR (brain attack):ti,ab,kw OR (ischemic stroke):ti,ab,kw OR (ischaemic stroke):ti,ab,kw OR (hemorrhagic stroke):ti,ab,kw OR (haemorrhagic stroke):ti,ab,kw OR (cerebral infarction):ti,ab,kw OR (brain infarction):ti,ab,kw OR (cerebral ischemia):ti,ab,kw OR (cerebral ischaemia):ti,ab,kw OR (brain ischemia):ti,ab,kw OR (brain ischaemia):ti,ab,kw OR (cerebral hemorrhage):ti,ab,kw OR (cerebral haemorrhage):ti,ab,kw OR (brain hemorrhage):ti,ab,kw OR (brain haemorrhage):ti,ab,kw OR (intracerebral hemorrhage):ti,ab,kw OR (intracerebral haemorrhage):ti,ab,kw OR (intracranial hemorrhage):ti,ab,kw OR (intracranial haemorrhage):ti,ab,kw OR (subarachnoid hemorrhage):ti,ab,kw OR (subarachnoid haemorrhage):ti,ab,kw OR (lacunar stroke):ti,ab,kw OR (lacunar infarction):ti,ab,kw
6	#4 OR #5
7	MeSH descriptor: [upper extremity] explode all trees
8	(upper extremity):ti,ab,kw OR (upper limb):ti,ab,kw OR (arm):ti,ab,kw OR (hand):ti,ab,kw OR (shoulder):ti,ab,kw OR (elbow):ti,ab,kw OR (wrist):ti,ab,kw
9	#7 OR #8
10	MeSH descriptor: [randomized controlled trial] explode all trees
11	(randomized controlled trial):ti,ab,kw OR (controlled clinical trial):ti,ab,kw OR (randomized):ti,ab,kw OR (placebo):ti,ab,kw OR (RCT):ti,ab,kw OR (randomised):ti,ab,kw
12	#10 OR #11
13	#3 AND #6 AND #9 AND #12

Table E2.5. Search strategy for Scopus

#	Searches
1	TITLE-ABS-KEY ("brain-computer interface" OR "brain-computer interfaces" OR "BCI" OR "brain-machine interface" OR "brain-machine interfaces" OR "brain machine interface" OR "brain machine interfaces" OR "neural interface" OR "neural interfaces" OR "neural interface technology" OR "neural interface technologies" OR "brain signal interface" OR "brain signal interfaces")
2	TITLE-ABS-KEY ("strokes" OR "acute stroke" OR "acute strokes" OR "cerebrovascular accident" OR "cerebrovascular accidents" OR "acute cerebrovascular accident" OR "acute cerebrovascular accidents" OR "CVA" OR "CVAs" OR "apoplexy" OR "brain attack" OR "ischemic stroke" OR "ischaemic stroke" OR "hemorrhagic stroke" OR "haemorrhagic stroke" OR "cerebral infarction" OR "brain infarction" OR "cerebral ischemia" OR "cerebral ischaemia" OR "brain ischemia" OR "brain ischaemia" OR "cerebral hemorrhage" OR "cerebral haemorrhage" OR "brain hemorrhage" OR "brain haemorrhage" OR "intracerebral hemorrhage" OR "intracerebral haemorrhage" OR "intracranial hemorrhage" OR "intracranial haemorrhage" OR "subarachnoid hemorrhage" OR "subarachnoid haemorrhage" OR "lacunar stroke" OR "lacunar infarction")
3	TITLE-ABS-KEY ("upper extremity" OR "upper limb" OR "arm" OR "hand" OR "shoulder" OR "elbow" OR "wrist")
4	TITLE-ABS-KEY ("randomized controlled trial" OR "controlled clinical trial" OR "randomized" OR "placebo" OR "RCT" OR "randomised")
5	#1 AND #2 AND #3 AND #4

Appendix E3. Intervention node definitions and classification

Node classification rule

Interventions were classified according to the primary therapeutic feedback modality and whether the feedback was contingent on BCI-decoded task-related brain activity in real time. A closed-loop BCI intervention was defined as one in which external feedback or an assistive device was triggered, modulated, or driven by BCI-decoded task-related brain activity in real time. Screen cues, task displays, user interfaces, or success/failure animations were not considered independent therapeutic feedback modalities unless they were explicitly described as part of the therapeutic closed-loop feedback process. For interventions combining multiple technological components, classification was based on the feedback modality most clearly described in the original report as therapeutic, real-time, and contingent on BCI-decoded activity. Definitions of all nodes are provided in Table E3.1, and the classification rationale for each intervention arm is provided in Table E3.2.

Table E3.1. Definitions of intervention nodes.

Node	Definition
BCI-MM	BCI-contingent feedback explicitly combining two or more therapeutic feedback modalities, such as visual/VR feedback, FES, tactile/proprioceptive feedback, auditory feedback, reward feedback, or robotic movement.
BCI-Robot	BCI-contingent control of a robotic device, exoskeleton, robotic glove, orthosis, Haptic Knob, or mechanical end-effector as the dominant therapeutic feedback.
BCI-FES	BCI-contingent functional electrical stimulation triggered, modulated, or driven by real-time BCI-decoded task-related brain activity as the dominant therapeutic feedback modality.
BCI-visual	BCI or neurofeedback in which task-related brain activity is mainly translated into visual feedback.
Robot	Robot-assisted training not triggered by real-time BCI-decoded brain activity.
FES	Functional electrical stimulation not triggered by real-time BCI-decoded brain activity.
MI	Motor imagery training without BCI-contingent external feedback.
UR	Usual rehabilitation, physical therapy, occupational therapy, standard arm therapy, or conventional rehabilitation without a distinct BCI, robot, FES, or MI component.
shamBCI-MM	Sham or non-contingent multimodal BCI feedback not driven by real-time BCI-decoded task-related brain activity.
shamBCI-Robot	Sham or non-contingent robotic BCI feedback not driven by real-time BCI-decoded task-related brain activity.
shamBCI-visual	Sham or non-contingent visual neurofeedback not driven by genuine task-related brain activity.

Note: BCI, brain-computer interface; FES, functional electrical stimulation; MI, motor imagery; UR, usual rehabilitation;

Table E3.2. Study-level intervention node classification and rationale.

Study	Intervention arm	Closed-loop BCI	Assigned node	Rationale
Ang 2014a	BCI-Manus	Yes	BCI-Robot	Real-time EEG-based motor imagery detection triggered Manus robotic movement of the paretic upper limb; the primary feedback modality was robotic movement.
	Manus	No	Robot	Manus robotic shoulder-elbow reaching training was provided, but the robotic movement was not triggered by real-time BCI-decoded brain activity.
Ang 2014b	BCI-HK	Yes	BCI-Robot	Real-time EEG-based motor imagery detection triggered Haptic Knob robot-assisted hand grasping and knob manipulation; the primary feedback modality was robotic movement.
	HK	No	Robot	Haptic Knob robot-assisted hand grasping and knob manipulation was provided, but robotic movement was not triggered by real-time BCI-decoded brain activity.
	SAT	No	UR	Standard arm therapy was therapist-assisted and did not include BCI, robotic, FES, or real-time MI-feedback components.
Cantillo-Negrete 2025	ReHand-BCI	Yes	BCI-Robot	Motor imagery-related brain activity controlled a robotic hand orthosis/device. The dominant closed-loop feedback was robotic hand movement; therefore, this arm was classified as BCI-Robot.
	Sham-BCI	No	shamBCI-Robot	The sham intervention used a similar robotic setup, but the feedback was not contingent on the participant's actual motor imagery-related brain activity; therefore, this arm was classified as shamBCI-Robot.
Chen 2020	BCI group	Yes	BCI-Robot	BCI detection of motor intention controlled an Omega exoskeleton to assist wrist extension. Visual cues were used mainly for task guidance, whereas the dominant therapeutic feedback was exoskeleton movement; therefore, this arm was classified as BCI-Robot.
	Control group	No	MI	Participants performed comparable motor imagery tasks without exoskeleton closed-loop feedback; therefore, this arm was classified as MI.

Cheng 2020	BCI-SRG	Yes	BCI-MM	BCI detection of motor imagery simultaneously activated the soft robotic glove and the visual feedback system. The intervention explicitly combined mechanical feedback from the soft robotic glove with task-specific visual feedback using a virtual arm and virtual objects during ADL-oriented tasks; therefore, this arm was classified as BCI-MM.
	SRG	No	Robot	Participants received soft robotic glove training without BCI-contingent control; therefore, this arm was classified as Robot.
Frolov 2017	MI-BCI hand exoskeleton	Yes	BCI-Robot	Motor imagery-related EEG controlled a hand exoskeleton to assist hand opening/movement. The dominant therapeutic feedback was hand-exoskeleton movement; therefore, this arm was classified as BCI-Robot.
	Sham exoskeleton	No	shamBCI-Robot	Exoskeleton movement was not contingent on genuine motor imagery-related brain activity; therefore, this arm was classified as shamBCI-Robot.
Fu 2023	BCI group	Yes	BCI-Robot	The functional-oriented portable BCI system decoded hand motor tasks and triggered robotic grasp/open training. The dominant feedback was robotic hand movement; therefore, this arm was classified as BCI-Robot.
	Control group	No	UR	Participants received task-oriented guidance training without BCI-contingent feedback; therefore, this arm was classified as UR.
Guo 2022	BCI-robotic	Yes	BCI-Robot	SSVEP-BCI was used to detect user intention and control a soft robotic glove. The visual flickering stimuli served as the BCI input/control paradigm rather than a co-dominant therapeutic visual feedback channel. The dominant therapeutic output was soft robotic glove movement; therefore, this arm was classified as BCI-Robot.
	Robotic	No	Robot	Participants received robotic glove therapy without BCI control; therefore, this arm was classified as Robot.
	Conventional	No	UR	Participants received conventional rehabilitation without BCI or robotic closed-loop feedback; therefore, this arm was classified as UR.

He 2025	BCI group	Yes	BCI-MM	Real-time EEG-derived motor intention scores regulated pedaling robot assistance and were accompanied by virtual reality interface, visual feedback, and auditory cues, indicating multimodal closed-loop feedback.
	Control group	No	shamBCI-MM	Participants used a similar device and training procedure, but feedback was generated from non-real-time/pretrained data rather than real-time EEG activity; therefore, this arm was classified as shamBCI-MM.
Hu 2021	MI-based BCI with multimodal feedback	Yes	BCI-MM	The original report explicitly described motor imagery-based BCI combined with sensory and visual feedback for upper-limb recovery; therefore, this arm was classified as BCI-MM.
	Classical MI training	No	MI	Participants received classical motor imagery training without BCI-contingent external feedback; therefore, this arm was classified as MI.
Ji 2025	BCI-SRG	Yes	BCI-Robot	BCI controlled the soft robotic glove to assist grasping tasks. Screen images and success/failure animations were mainly used for task cueing and feedback flow, whereas the dominant BCI-contingent therapeutic feedback was robotic hand movement; therefore, this arm was classified as BCI-Robot.
	SRG	No	Robot	Participants received soft robotic glove training without BCI-contingent control; therefore, this arm was classified as Robot.
Kim 2025	MI-contingent feedback BCI group	Yes	BCI-MM	The recoveriX-PRO system combined EEG-based motor imagery detection, FES of the wrist extensors, and visual feedback through virtual avatar hands. During the feedback phase, both FES and the virtual avatar were activated, establishing contingent visual and proprioceptive feedback. Therefore, this arm was classified as BCI-MM rather than BCI-FES.
	MI-independent feedback BCI group	No	shamBCI-MM	Participants used the same multimodal feedback system and performed the same motor imagery task, but feedback was provided independently of motor imagery-related brain activity; therefore, this arm was classified as shamBCI-MM / non-contingent BCI-MM.

Lu 2025	Multi-FDBK-BCI	Yes	BCI-MM	The system explicitly integrated proprioceptive, tactile, and visual feedback. BCI detection of motor intention simultaneously activated exoskeleton movement, tactile stimulation, and VR-based visual feedback; therefore, this arm was classified as BCI-MM.
	Conventional motor imagery therapy	No	MI	Participants received conventional motor imagery therapy without BCI-contingent multimodal feedback; therefore, this arm was classified as MI.
Miao 2020	BCI group	Yes	BCI-FES	MI-BCI detection of wrist dorsiflexion imagery triggered FES, and FES was the primary peripheral therapeutic output; virtual-limb visual feedback was treated as accompanying feedback.
	Control group	No	UR	Participants received routine rehabilitation without BCI-contingent feedback; therefore, this arm was classified as UR.
Mihara 2013	REAL neurofeedback	Yes	BCI-visual	NIRS-based motor-related cortical activity was translated into real visual neurofeedback. The primary feedback modality was visual neurofeedback; therefore, this arm was classified as BCI-visual.
	SHAM neurofeedback	No	shamBCI-visual	Visual feedback was unrelated to genuine brain activity or randomly generated; therefore, this arm was classified as shamBCI-visual.
Ramos-Murguialday 2013	Experimental BMI	Yes	BCI-Robot	Ipsilesional SMR desynchronization triggered online movements of hand and arm orthoses. The dominant therapeutic feedback was orthotic/robotic movement; therefore, this arm was classified as BCI-Robot.
	Sham BMI	No	shamBCI-Robot	Orthosis movements occurred randomly and were unrelated to SMR control; therefore, this arm was classified as shamBCI-Robot.
Wang 2024	BCI group	Yes	BCI-MM	BCI rehabilitation training combined motor imagery with VR and FES in addition to traditional rehabilitation. Because the feedback involved multiple channels, this arm was classified as BCI-MM.
	Control group	No	UR	Participants received traditional rehabilitation training without BCI-contingent feedback; therefore, this arm was classified as UR.

Liu 2023	MI-BCI group	Yes	BCI-FES	Real-time EEG identification of motor imagery triggered FES, and FES was the primary peripheral therapeutic output.
	CR group	No	FES	The control arm received functional electrical stimulation in addition to conventional rehabilitation, but stimulation was not triggered by real-time BCI activity.
Ma 2023	BCI group	Yes	BCI-MM	BCI drove exoskeleton-assisted training and provided auditory/visual reward or performance feedback in addition to exoskeleton movement.
	Control group	No	UR	Participants received conventional physical/occupational therapy without BCI-contingent feedback; therefore, this arm was classified as UR.
Li 2022	SMR-BCI group	Yes	BCI-MM	The intervention was explicitly described as SMR-BCI with audio-cue, motor observation, and multisensory feedback, including robotic, auditory, and visual feedback; therefore, this arm was classified as BCI-MM.
	Control group	No	UR	Participants received conventional therapy without SMR-BCI feedback; therefore, this arm was classified as UR.
Wu 2020	BCI group / BG	Yes	BCI-MM	MI-related mu suppression triggered exoskeleton-hand movement and was accompanied by video guidance and real-time visual score feedback.
	Control group / CG	No	UR	The control arm received routine rehabilitation without BCI, robotic, or real-time closed-loop feedback.
Pichiorri 2015	BCI group	Yes	BCI-visual	EEG-based motor imagery rhythm modulation drove real-time virtual-hand visual feedback; the primary feedback modality was non-immersive visual neurofeedback.
	CTRL group	No	MI	The control arm received the same motor imagery training without BCI support and without real-time brain activity-contingent feedback.

Appendix E4: Characteristics of included studies and data conversion methods

Table E4.1. Baseline and intervention characteristics of included studies.

Study	Node	n	Age, years	Stroke duration, days	Stroke phase	Baseline FMA-UE	FMA-UE change	Training dose	Outcome measures
Ang 2014a	BCI-Robot	11	48.5 ± 13.5	383.0 ± 290.8	Chronic	26.3 ± 10.3	4.5 ± 12.43	4 weeks, 4 sessions/week, 90 min/session	①
	Robot	14	53.6 ± 9.5	234.7 ± 183.8	Chronic	26.6 ± 18.9	6.3 ± 20.27		
Ang 2014b	BCI-Robot	6	54.0 ± 8.9	285.7 ± 64.0	Chronic	33.0 ± 16.2	7.2 ± 2.3	6 weeks, 3 sessions/week, 90 min/session	①
	Robot	8	51.1 ± 6.3	398.2 ± 150.9	Chronic	25.5 ± 11.5	7.3 ± 4.7		
	UR	7	58.0 ± 19.3	455.4 ± 109.6	Chronic	23.4 ± 14.5	4.9 ± 4.1		
Cantillo-Negrete 2025	BCI-Robot	10	53.2 ± 15.1	368.1 ± 183.0	Mixed	21.6 ± 10.8	6.6 ± 5.6	6 weeks, 5 sessions/week	①, ②
	shamBCI-Robot	9	55.8 ± 16.0	316.8 ± 179.4	Mixed	19.9 ± 11.2	2.1 ± 3.0		
Chen 2020	BCI-Robot	7	41.6 ± 12.0	93.0 ± 51.0	Subacute	26.8 ± 11.9	4.5 ± 2.6	4 weeks, 3 sessions/week; 1 h/session	①
	MI	7	52.0 ± 11.1	117.0 ± 45.0	Subacute	26.5 ± 15.9	6.2 ± 4.1		
Cheng 2020	BCI-MM	5	62.4 ± 4.7	476.8 ± 302.0	Chronic	31.3 ± 11.5	8.4 ± 8.0	6 weeks, 3 sessions/week, 120 min/session	①, ②
	Robot	5	61.4 ± 4.5	890.2 ± 257.23	Chronic	32.3 ± 11.8	4.7 ± 5.0		
Frolov 2017	BCI-Robot	55	57.0 ± 12.6	253.3 ± 202.7	Mixed	24.2 ± 7.01	3.80 ± 5.36	2 weeks, 5 sessions/week, 30 min/session	①, ②
	shamBCI-Robot	19	59.0 ± 11.1	222.9 ± 270.2	Mixed	30.0 ± 7.84	5.60 ± 2.61		
Fu 2023	BCI-Robot	30	55.93 ± 11.05	77.50 ± 33.75	Subacute	25.3 ± 20.7	4.67 ± 22.90	4 weeks, 5 sessions/week, 30 min/session	①
	UR	31	59.00 ± 14.49	64.00 ± 37.00	Subacute	24.0 ± 28.1	2.67 ± 28.52		
Guo 2022	UR	10	53.5 ± 8.3	327.0 ± 237.0	Chronic	16.8 ± 14.1	10.92 ± 7.96	2 weeks, 5 sessions/week, 60 min/session	①, ③
	Robot	10	56.9 ± 6.1	351.0 ± 162.0	Chronic	23.3 ± 22.2	5.67 ± 2.96		
	BCI-Robot	10	60.2 ± 9.3	375.0 ± 213.0	Chronic	14.4 ± 8.69	0.8 ± 1.32		
He 2025	BCI-MM	25	58.6 ± 12.8	33.3 ± 18.5	Subacute	14.9 ± 5.23	7.05 ± 4.36	2 weeks, 5 sessions/week, 20 min/session	①
	shamBCI-MM	23	58.3 ± 12.8	41.8 ± 19.1	Subacute	14.9 ± 7.98	10.5 ± 8.03		
Hu 2021	BCI-MM	7	44.9 ± 7.5	237.0 ± 195.0	Mixed	26.7 ± 25.9	4.33 ± 2.22	Not clearly reported; 30 min/session	①, ②
	MI	5	60.4 ± 16.8	219.0 ± 135.0	Chronic	19.3 ± 13.3	2.33 ± 2.22		
Ji 2025	BCI-Robot	20	61.75 ± 10.35	49.50 ± 23.36	Subacute	12.70 ± 8.88	2.70 ± 9.55	4 weeks, 5 sessions/week, 20 – 25 min/session	①, ②
	Robot	19	60.05 ± 14.35	42.42 ± 24.50	Subacute	13.80 ± 6.65	6.80 ± 10.98		

Kim 2025	BCI-MM	12	49.0 ± 16.9	546.0 ± 486.0	Chronic	22.5 ± 6.3	18.0 ± 10.37	4 weeks, 5 sessions/week, 60 min/session	①
	shamBCI-MM	13	46.0 ± 12.8	656.0 ± 702.0	Chronic	21.7 ± 4.4	10.0 ± 9.63		
Lu 2025	BCI-MM	20	52.60 ± 11.48	360.0 ± 207.0	Chronic	29.92 ± 11.95	1.42 ± 13.12	4 weeks, 5 sessions/week, 30 min/session	①, ②
	MI	19	50.42 ± 13.61	276.3 ± 216.0	Chronic	33.54 ± 11.10	2.77 ± 11.25		
Miao 2020	BCI-FES	8	48.75 ± 16.85	549.0 ± 327.0	Chronic	13.90 ± 6.97	4.40 ± 3.30	4 weeks, 3 sessions/week; session length not fully extracted	①
	UR	8	50.25 ± 17.82	333.0 ± 150.0	Chronic	13.68 ± 6.28	2.26 ± 1.63		
Mihara 2013	BCI-visual	10	58 (median)	152.5 ± 37.0	Subacute	19.50 ± 9.86	3.50 ± 5.18	2 weeks, 3 sessions/week, 20 min/session	①, ②
	shamBCI-visual	10	62 (median)	106.5 ± 37.0	Subacute	20.63 ± 9.66	0.88 ± 1.25		
Ramos-Murguialday 2013	BCI-Robot	16	49.3 ± 12.5	2009.0 ± 1369.8	Chronic	20.7 ± 13.8	5.0 ± 14.6	4 weeks, 5 sessions/week; session length not clearly reported	①
	shamBCI-Robot	14	50.3 ± 12.2	2161.2 ± 2191.7	Chronic	24.0 ± 13.8	2.3 ± 14.1		
Wang 2024	BCI-MM	150	60 (52–67), median	14.7 ± 9.6	Subacute	11.16 ± 6.92	3.41 ± 2.25	4 weeks, 5 sessions/week, 30 min/session	①, ②, ③
	UR	146	58 (52–66), median	13.0 ± 7.4	Subacute	13.29 ± 10.70	0.35 ± 4.15		
Liu 2023	BCI-FES	30	52.5 (45.0, 59.3)	18.3 ± 9.1	Subacute	30.33 ± 20.00	13.2 ± 10.09	3 weeks, 5 sessions/week, 20 min/session	①, ③
	FES	30	53.0 (38.5, 59.5)	16.9 ± 9.8	Subacute	33.00 ± 26.67	9.83 ± 10.11		
Ma 2023	BCI-MM	20	50.90 ± 12.64	193.5 ± 101.4	Mixed	31.0 ± 15.2	11.8 ± 6.1	2 weeks, 5 sessions/week, 40 min/session	①
	UR	20	58.30 ± 11.23	177.0 ± 89.7	Mixed	34.4 ± 14.3	3.9 ± 1.6		
Li 2022	BCI-MM	12	43.8 ± 14.7	129.0 ± 78.0	Mixed	20.80 ± 11.21	9.10 ± 11.15	2 weeks, 5 sessions/week, 60 min/session	①, ③
	UR	12	55.0 ± 12.2	173.1 ± 206.7	Mixed	20.75 ± 10.77	1.65 ± 10.68		
Wu 2020	BCI-MM	14	62.93 ± 10.56	63.3 ± 9.0	Subacute	22.58 ± 13.71	3.93 ± 2.44	4 weeks, 5 sessions/week, 60 min/session	①, ②, ③
	UR	11	64.82 ± 7.22	60.0 ± 22.5	Subacute	24.25 ± 17.08	4.70 ± 3.90		
Pichiorri 2015	BCI-visual	14	64.1 ± 8.4	82.2 ± 51.7	Subacute	18.43 ± 2.65	16.93 ± 2.56	4 weeks, 3 sessions/week, 30 min/session	①
	MI	14	59.6 ± 12.7	76.1 ± 36.5	Subacute	14.09 ± 2.51	8.36 ± 2.12		

Notes: Outcome measures: ① FMA-UE, Fugl-Meyer Assessment of the Upper Extremity; ② ARAT, Action Research Arm Test; ③ WMFT, Wolf Motor Function Test. Abbreviations: BCI, brain-computer interface; FES, functional electrical stimulation; MI, motor imagery; SRG, soft robotic glove; UL, upper limb.

Appendix E4.2 Data conversion methods for continuous outcomes

FMA-UE, ARAT, and WMFT were treated as continuous outcomes. The direction of effect was harmonized so that positive values indicated greater improvement in upper-limb motor function after the intervention. When available, change-score means and standard deviations reported in the original articles were used. When change-score data were not directly reported, the following conversion rules were applied. If individual-level data were provided in the article or supplementary materials, the corresponding means and standard deviations were calculated directly.

1. Mean change

When the mean change was not reported but baseline and post-intervention means were available, the mean change was calculated as:

$$Mean_{change} = Mean_{post} - Mean_{pre}$$

where Mean_post and Mean_pre denote the post-intervention and baseline means, respectively.

2. Standard deviation of change scores

When the standard deviation of the change score was not reported, it was estimated from the baseline and post-intervention standard deviations as:

$$SD_{change} = \sqrt{SD_{pre}^2 + SD_{post}^2 - 2 \times r \times SD_{pre} \times SD_{post}}$$

where SD_pre and SD_post denote the baseline and post-intervention standard deviations, and r denotes the within-participant correlation between baseline and post-intervention measurements. Because most studies did not report this correlation, r=0.5 was prespecified and applied consistently during data extraction and analysis.

3. Medians and interquartile ranges

When continuous data were reported as medians and interquartile ranges, means and standard deviations were estimated as:

$$Mean \approx \frac{Q1 + Median + Q3}{3}$$

$$SD \approx \frac{Q3 - Q1}{1.35}$$

where Q1 and Q3 denote the first and third quartiles, respectively. If the original report provided median and interquartile range values for the change score, those change-score data were converted preferentially.

4. Standard errors and 95% confidence intervals

When standard errors were reported, standard deviations were calculated as:

$$SD = SE \times \sqrt{n}$$

where SE denotes the standard error and n denotes the group sample size.

When 95% confidence intervals were reported for means or change scores, standard errors were first estimated as:

$$SE = \frac{Upper\ limit - Lower\ limit}{2 \times 1.96}$$

Standard deviations were then calculated using SD=SE× √n. When other statistics allowed the standard error to be derived, the standard error was first obtained and then converted to a standard deviation.

Table E4.2. Data handling for FMA-UE change scores across included studies

Data handling approach	No.	Studies	Method used for the analysis
Directly reported change-score mean and SD	6	Ang 2014b; Cantillo-Negrete 2025; Cheng 2020; Guo 2022; Lu 2025; Wu 2020	No transformation of the change-score data was required.
Change scores derived from baseline and post-intervention data	7	Ang 2014a; Frolov 2017; Hu 2021; Kim 2025; Mihara 2013; Ma 2023; Pichiorri 2015	Mean change = post − baseline; SD_{change}estimated assuming
Change scores reported as median and IQR	5	Fu 2023; He 2025; Ji 2025; Liu 2023; Li 2022	Median and IQR were converted to mean and SD using the methods described above.
Individual participant-level data available	2	Chen 2020; Miao 2020	Change-score means and SDs were calculated directly from the reported participant-level data.
SD derived from other reported summary statistics	2	Ramos-Murguialday 2013; Wang 2024	SDs were derived from reported SEs, 95% CIs, or other available statistics as described above.

Note: Sixteen of the 22 studies required transformation or derivation of FMA-UE change-score data. In seven studies, $SD_{\text{change}}$was estimated from baseline and post-intervention SDs assuming a within-participant correlation of $r = 0.5$.

Appendix E5. Risk of bias, certainty of evidence, and small-study effects

Table E5.1. Risk-of-bias assessment using the Cochrane RoB 2 tool.

Study	D1	D2	D3	D4	D5	Overall	Note
Ang 2014a	Some concerns	Some concerns	Low risk	Low risk	Some concerns	Some concerns	Randomization was reported, but the allocation method was unclear; participants were not blinded, and the intention-to-treat (ITT) analysis was not explicitly stated; there was only one dropout, and assessor blinding was adequate; the pre-specified analysis plan was not described; therefore, the overall rating is "Some concerns."
Ang 2014b	Some concerns	Some concerns	Low risk	Low risk	Some concerns	Some concerns	The study reported randomization, but the allocation concealment was unclear; participants and implementers were not blinded, and the intention-to-treat (ITT) analysis was not specified; there was only one dropout, and assessor blinding was adequate; although the study was registered, no pre-specified analysis plan was available; therefore, the overall rating is "Some concerns."
Cantillo-Negrete 2025	Low risk	Some concerns	Some concerns	Low risk	Some concerns	Some concerns	The trial was a triple-blind study, and the outcome assessment was well-blinded; however, the allocation concealment was not specified, and some participants were excluded from the final analysis after randomization. The absence of bias in the missing data was not adequately addressed. No pre-specified analysis plan was observed; therefore, the overall rating is "Some concerns."
Chen 2020	Some concerns	Some concerns	Low risk	Some concerns	Some concerns	Some concerns	This study only describes randomization; the random sequence and allocation are not clearly defined; information on intervention allocation and blinding is limited; although no obvious missing data were observed, the assessor blinding is not specified, and there is no information on registration or a standard allocation plan (SAP). Therefore, the overall rating is "Some concerns."
Cheng 2020	Some concerns	Some concerns	Low risk	Low risk	Some concerns	Some concerns	The report describes randomization but the allocation concealment is unclear; the intention-to-treat (ITT) analysis is not specified and there was one dropout; the assessor was clearly blinded; although the study was registered, the pre-specified analysis plan was not described; therefore, the overall rating is "Some concerns."

Frolov 2017	Some concerns	Some concerns	Some concerns	Low risk	Some concerns	Some concerns	For a closed-loop study comparing true versus sham feedback, the use of a double-blind design is preferable; however, the allocation concealment method was not specified, and of the 89 patients randomized, only 74 were included in the analysis, with missing data not adequately addressed. No pre-specified analysis plan was observed; therefore, the overall rating is “Some concerns.”
Fu 2023	Low risk	Some concerns	Low risk	Low risk	Some concerns	Some concerns	The use of opaque envelopes and assessor blinding supports lower risk; however, the intention-to-treat (ITT) analysis was not clearly defined, and five cases were excluded from the analysis after randomization, with insufficient handling of missing data. Although the study was registered, no pre-specified analysis plan was available; therefore, the overall assessment is “Some concerns.”
Guo 2022	Some concerns	Low risk	Low risk	Some concerns	Some concerns	Some concerns	The study used randomization but the allocation concealment was unclear; all participants completed the intervention and there were no significant missing data; however, the assessor blinding was not specified, and no pre-specified analysis plan was available after registration. Therefore, the overall rating is “Some concerns.”
He 2025	Low risk	Low risk	Some concerns	Low risk	Some concerns	Some concerns	The study employed a double-blind design with clear assessor blinding; however, only 48 of the 52 randomized patients were included in the analysis, and no adequate evidence of unbiased handling of missing data was provided; furthermore, there was no clear information regarding registration or the Standardized Analysis Protocol (SAP). Therefore, the overall rating is “Some concerns.”
Hu 2021	Some concerns	Low risk	Low risk	Low risk	Some concerns	Some concerns	Although randomization was mentioned, there was insufficient detail regarding the random sequence and allocation; all procedures were completed and the assessor was well-blinded; although the study was registered, the pre-specified analysis plan was not described; therefore, the overall rating is “Some concerns.”
Ji 2025	Low risk	Some concerns	Low risk	Some concerns	Some concerns	Some concerns	Envelopes were generated and sealed by computer, resulting in good randomization quality; however, the ITT definition was not clearly specified and there was one withdrawal, and the blinding of clinical scale assessors was unclear. The study was registered as retrospective and no pre-specified analysis plan was available; therefore, the overall rating is “Some concerns.”

Kim 2025	Some concerns	Some concerns	Some concerns	Low risk	Some concerns	Some concerns	The study used computer-generated randomization and blinded participants and outcome assessors, which supports internal validity. However, allocation concealment was not described, two randomized participants were excluded from the final analysis without a clearly reported intention-to-treat approach for this outcome, and the article did not describe a pre-specified statistical analysis plan despite trial registration. Therefore, the overall risk of bias for FMA-UE was judged as Some concerns.
Lu 2025	Some concerns	Low risk	Low risk	Low risk	Some concerns	Some concerns	The report describes a random number sequence, but the allocation method is not specified; all interventions and assessments were completed, and the assessor's blinding status is clear; although a prospective registration was conducted, no pre-specified analysis plan was identified; therefore, the overall rating is "Some concerns."
Miao 2020	Some concerns	Some concerns	Low risk	Some concerns	Some concerns	Some concerns	The report only describes randomization; the methods and allocation concealment are unclear; the intention-to-treat (ITT) analysis is not explicitly stated; although no significant loss to follow-up was observed, the assessor blinding is not described, and there is no information on registration or a standard allocation procedure (SAP); therefore, the overall rating is "Some concerns."
Mihara 2013	Low risk	Low risk	Low risk	Low risk	Some concerns	Some concerns	The study reported random allocation, likely concealed assignment, blinded intervention personnel, blinded outcome assessors, and complete outcome data, which all support good internal validity. However, the article did not describe trial registration or a pre-specified statistical analysis plan, and multiple eligible time points were available for reporting. Therefore, the overall risk of bias for the hand/finger FMA result was judged as Some concerns.
Ramos 2013	Some concerns	Some concerns	Some concerns	Low risk	Some concerns	Some concerns	Participants, therapists, and assessment personnel were all adequately blinded; however, the allocation concealment was not specified, two participants were excluded from the control group after randomization, a modified cFMA was used, and two baseline assessments were combined. No pre-specified analysis plan was observed; therefore, the overall rating is "Some concerns."
Wang 2024	Low risk	Low risk	Low risk	Low risk	Low risk	Low risk	Given the use of central randomization and computer-generated sequences, the ITT analysis, adequate assessor blinding, missing data supported by per-protocol (PP) and sensitivity analyses, and the availability of the protocol and

							study protocol, the overall risk is assessed as Low.
Liu 2023	Low risk	Low risk	Low risk	Low risk	Some concerns	Some concerns	The use of computer-generated random sequences, opaque envelopes, ITT analysis and imputation, and assessor blinding is commendable; however, although the study was registered, the article does not describe a pre-specified statistical analysis plan, so the overall rating remains “Some concerns.”
Ma 2023	Some concerns	Some concerns	Some concerns	Low risk	Some concerns	Some concerns	Computer randomization was described, but the allocation concealment was unclear; a per-protocol analysis was used, and there were 6 dropouts after randomization, so bias due to missing data cannot be ruled out; assessor blinding was clearly stated, but no pre-specified analysis plan was provided; therefore, the overall rating is “Some concerns.”
Li 2022	Some concerns	Low risk	Low risk	Some concerns	Some concerns	Some concerns	The study used randomization but the allocation concealment was unclear; all participants were included in the analysis and there were no apparent missing data; however, the blinding of clinical scale assessors was not specified, and although the study was registered, there was no pre-specified analysis plan. Therefore, the overall assessment is “Some concerns.”
Wu 2020	Some concerns	Low risk	Low risk	Low risk	Some concerns	Some concerns	A random number sequence was used, but the allocation method was not specified; all participants completed the intervention and the assessor was clearly blinded; although the study was registered, the pre-specified analysis plan was not described; therefore, the overall rating is “Some concerns.”
Pichiorri 2015	Low risk	Some concerns	Low risk	Low risk	Low risk	Some concerns	This study employed randomization and concealed allocation, baseline characteristics were comparable, and the primary outcome measures were predefined and fully reported. Outcome assessors were blinded, and measurement bias was considered low. However, because neither participants nor therapists were blinded and no intention-to-treat analysis was reported, there were some concerns regarding deviations from intended interventions; therefore, the overall rating was judged as “Some concerns.”

Notes: RoB 2, revised Cochrane risk-of-bias tool for randomized trials; D1, bias arising from the randomization process; D2, bias due to deviations from intended interventions; D3, bias due to missing outcome data; D4, bias in measurement of the outcome; D5, bias in selection of the reported result. Overall judgments were categorized as low risk, some concerns, or high risk.

Table E5.2. CINeMA assessment of certainty of evidence for FMA-UE.

Comparison	Within-study bias	Reporting bias	Indirectness	Imprecision	Heterogeneity	Incoherence	Confidence rating
BCI-FES vs UR	Some concerns	Low risk	No concerns	Some concerns	Some concerns	Some concerns	Very low
BCI-MM vs UR	Some concerns	Low risk	No concerns	No concerns	Some concerns	No concerns	Low
BCI-Robot vs UR	Some concerns	Low risk	No concerns	No concerns	Some concerns	No concerns	Low
Robot vs UR	Some concerns	Low risk	No concerns	No concerns	Some concerns	No concerns	Low
BCI-visual vs UR	Some concerns	Low risk	No concerns	Major concerns	No concerns	Some concerns	Very low
FES vs UR	Some concerns	Low risk	No concerns	Some concerns	Some concerns	Some concerns	Very low
MI vs UR	Some concerns	Low risk	No concerns	Some concerns	Some concerns	Some concerns	Very low
shamBCI-MM vs UR	Some concerns	Low risk	No concerns	Some concerns	Some concerns	Some concerns	Very low
shamBCI-Robot vs UR	Some concerns	Low risk	No concerns	Some concerns	No concerns	Some concerns	Very low
shamBCI-visual vs UR	Some concerns	Low risk	No concerns	Major concerns	No concerns	Some concerns	Very low

Note: The clinically important effect size for FMA-UE was prespecified as 5.25 points and was used to judge imprecision, heterogeneity, and incoherence. "—" indicates that direct evidence was unavailable or not applicable for the comparison.

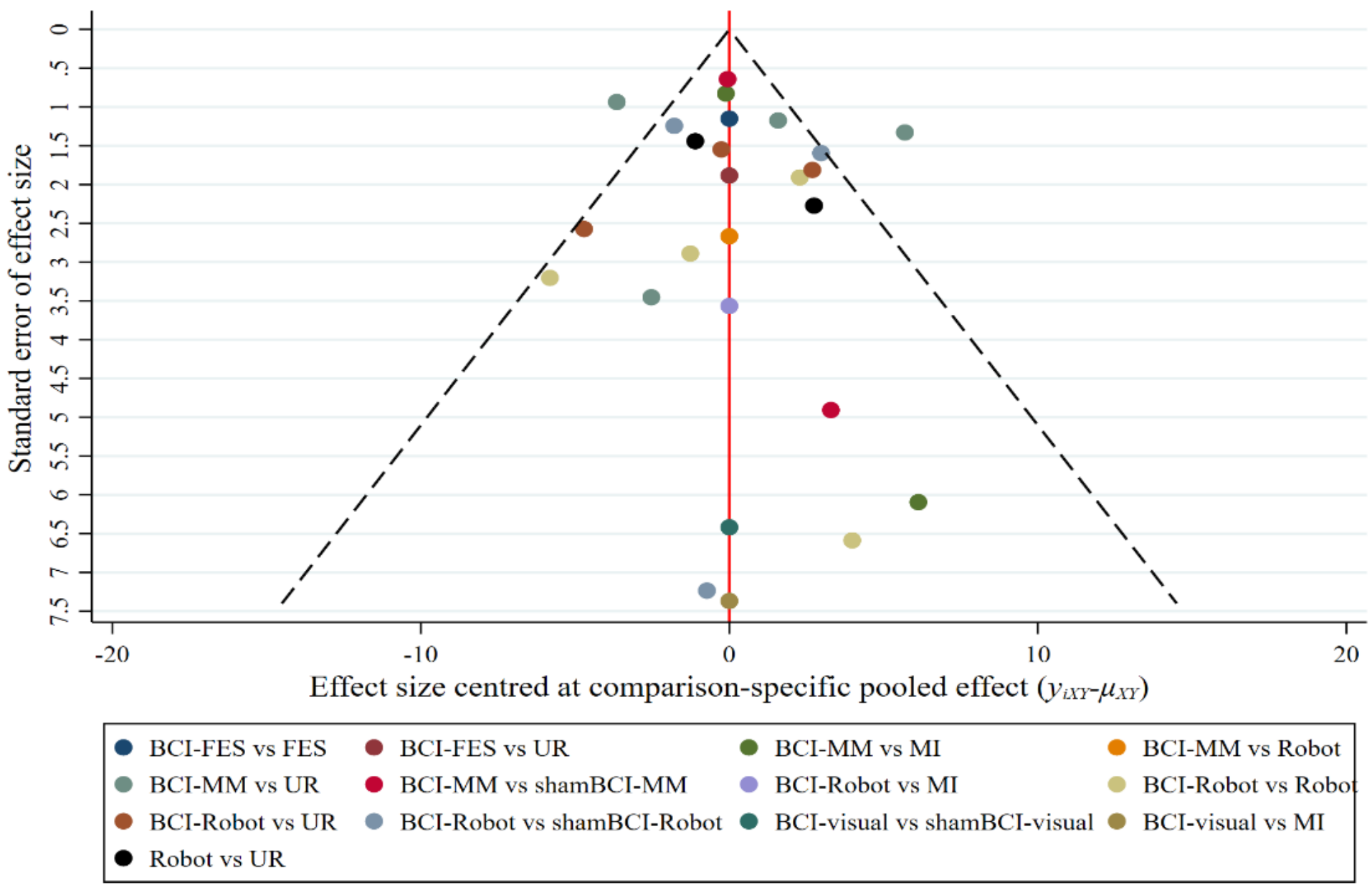

Figure E5.3. Comparison-adjusted funnel plot for FMA-UE

Appendix E6. Supplementary analyses for the primary outcome FMA-UE

Figure E6.1. Contribution matrix for the FMA-UE network

Direct comparisons in the network (rows: network meta-analysis estimates)

Network meta-analysis estimates	AvsE	AvsH	BvsF	BvsG	BvsH	BvsI	CvsF	CvsG	CvsH	CvsJ	DvsF	DvsK	GvsH
**Mixed estimates**													
AvsE	99.1	0.3	0.1		0.1				0.1		0.1		0.1
AvsH	0.1	99.4	0.1		0.1				0.1				0.1
BvsF			82.9	2.5	3.1		5.6	2.5	3.1		0.1	0.1	
BvsG		0.1	9.6	26.0	20.9	0.1	9.4	12.5	3.0	0.1	0.1	0.1	18.0
BvsH	0.1	0.2	10.1	17.8	31.3	0.1	9.8	2.5	12.5	0.1	0.2	0.1	15.3
BvsI						99.9							
CvsF		0.1	28.5	12.7	15.6	0.1	14.1	12.8	15.7	0.1	0.2	0.1	
CvsG			6.4	8.4	2.0		6.3	34.7	22.0	0.1			20.1
CvsH	0.1	0.1	6.6	1.7	8.4		6.6	18.8	40.4	0.1			17.1
CvsJ		0.1		0.1	0.1		0.1	0.1	0.2	99.2	0.1		
DvsF	0.8	1.7	5.4	1.5	2.1	0.9	1.5	0.3		0.9	80.7	3.8	0.4
DvsK	0.3	0.7	2.1	0.6	0.8	0.3	0.6	0.1		0.3	3.1	90.8	0.2
GvsH	0.1	0.1		11.1	11.1			18.5	18.5				40.4
**Indirect estimates**													
AvsB	0.1	37.2	6.4	11.2	19.7	0.1	6.2	1.6	7.8		0.1	0.1	9.6
AvsC	0.1	39.7	4.0	1.1	5.0		4.0	11.4	24.4	0.1			10.3
AvsD	0.3	23.7	17.1	6.2	11.4	0.2	5.2	0.3	5.9	0.3	22.2	1.1	6.1
AvsF	0.1	30.3	23.3	8.3	15.0		7.0	0.4	7.4		0.1	0.1	7.8
AvsG	0.1	41.2		6.6	6.5			10.9	10.9				23.8
AvsI	0.1	27.1	4.6	8.2	14.4	27.2	4.5	1.2	5.7		0.1		7.0
AvsJ	0.1	28.5	2.9	0.7	3.6		2.9	8.1	17.4	28.4			7.4
AvsK	0.3	19.7	13.9	5.0	9.3	0.2	4.2	0.2	5.0	0.3	17.9	18.8	5.1
BvsC		0.1	13.5	16.3	20.0	0.1	13.3	16.3	20.1	0.1	0.1	0.1	0.1
BvsD	0.5	0.9	41.5	2.2	2.8	0.5	2.2	1.5	1.6	0.5	43.5	2.1	0.2
BvsJ			9.0	10.9	13.4	0.1	8.9	10.9	13.4	33.2	0.1	0.1	
BvsE	27.1	27.1	4.7	8.2	14.4	0.1	4.5	1.2	5.7		0.1		7.0
BvsK	0.5	0.9	28.8	1.8	2.3	0.5	1.4	1.1	1.1	0.5	29.9	31.2	0.2
CvsD	0.3	0.7	18.8	8.7	10.6	0.3	9.7	9.2	11.4	0.4	28.4	1.4	0.2
CvsI		0.1	9.0	10.9	13.4	33.3	8.9	10.9	13.4	0.1	0.1		
CvsE	28.5	28.4	2.8	0.8	3.6		2.9	8.1	17.5	0.1			7.4
CvsK	0.4	0.7	14.4	6.8	8.2	0.3	7.6	7.3	9.1	0.4	21.8	22.8	0.2
DvsG	0.3	0.7	21.7	12.7	9.6	0.3	6.5	7.8	0.6	0.3	28.2	1.4	10.0
DvsH	0.4	0.7	22.3	8.0	14.8	0.3	6.7	0.4	7.7	0.3	28.9	1.4	8.0
DvsI	0.3	0.6	28.3	1.5	1.9	32.1	1.5	1.0	1.1	0.3	29.7	1.4	0.1
DvsJ	0.2	0.5	14.4	6.7	8.1	0.2	7.5	7.0	8.8	23.5	21.8	1.1	0.1
DvsE	19.4	19.2	13.9	5.0	9.2	0.2	4.2	0.2	4.8	0.2	18.0	0.9	5.0
FvsG		0.1	32.5	18.2	14.2		9.7	10.6	0.8		0.2	0.1	13.5
FvsH	0.1	0.2	33.5	11.8	21.5	0.1	10.0	0.6	10.7		0.2	0.1	11.3
FvsI			44.0	1.3	1.7	47.0	3.0	1.3	1.6		0.1		
FvsJ			20.0	8.9	11.0	0.1	9.9	9.0	11.0	29.9	0.1	0.1	
FvsE	23.3	23.3	17.9	6.3	11.5		5.4	0.3	5.7		0.1	0.1	6.0
FvsK	0.6	1.3	4.1	1.2	1.6	0.7	1.1	0.2		0.6	43.2	45.0	0.3
GvsI		0.1	6.1	16.6	13.4	36.1	6.0	8.0	1.9		0.1		11.5
GvsJ			3.9	5.2	1.2		3.9	21.3	13.5	38.6			12.3
GvsE	29.2	29.1		4.7	4.6			7.8	7.7				16.8
GvsK	0.4	0.7	16.8	10.0	7.4	0.3	5.1	6.3	0.5	0.4	21.7	22.7	8.0
HvsI	0.1	0.1	6.3	11.2	19.7	37.2	6.2	1.6	7.8		0.1		9.6
HvsJ	0.1	0.1	4.0	1.0	5.1		4.0	11.3	24.4	39.7			10.3
HvsE	49.8	49.7	0.1		0.1				0.1		0.1		0.1
HvsK	0.4	0.8	17.2	6.2	11.6	0.3	5.2	0.2	6.1	0.4	22.2	23.2	6.3
IvsJ			6.8	8.2	10.0	25.0	6.7	8.2	10.0	24.9	0.1		
IvsE	21.3	21.3	3.7	6.4	11.3	21.4	3.6	0.9	4.5		0.1		5.5
IvsK	0.3	0.7	21.7	1.3	1.7	25.1	1.0	0.8	0.9	0.3	22.5	23.5	0.2
JvsE	22.2	22.1	2.2	0.6	2.8		2.2	6.3	13.6	22.2			5.7
JvsK	0.3	0.6	11.6	5.5	6.6	0.2	6.1	5.9	7.3	19.6	17.6	18.4	0.1
EvsK	16.7	16.4	11.6	4.2	7.8	0.2	3.5	0.2	4.1	0.2	15.0	15.7	4.3
**Entire network**	6.4	11.4	13.2	6.6	9.1	6.4	5.0	5.3	8.0	6.4	10.2	6.1	5.9
**Included studies**	1	1	2	1	4	2	1	4	3	3	1	1	2

Table E6.1. Direct pairwise meta-analysis results for FMA-UE.

Direct comparison	No. of studies	WMD (95% CI)	P value	I^2 (%)	τ^2
BCI-Robot vs Robot	4	2.72 (-1.33, 6.76)	0.188	43.3%	7.1238
BCI-Robot vs UR	3	5.38 (1.73, 9.04)	0.004	64.2%	6.6200
Robot vs UR	2	4.73 (1.04, 8.42)	0.012	51.2%	3.7923
BCI-Robot vs shamBCI-Robot	3	0.90 (-3.09, 4.88)	0.659	64.0%	6.8570
BCI-Robot vs MI	1	3.70 (-3.29, 10.69)	0.299	—	—
BCI-MM vs Robot	1	-1.80 (-7.03, 3.43)	0.500	—	—
BCI-MM vs shamBCI-MM	2	1.94 (0.70, 3.19)	0.002	0.0%	0.0000
BCI-MM vs MI	2	1.94 (-0.21, 4.09)	0.076	2.9%	0.5561
BCI-MM vs UR	4	4.45 (-0.26, 9.15)	0.064	91.6%	19.8162
BCI-FES vs FES	1	7.90 (5.64, 10.16)	<0.001	—	—
BCI-FES vs UR	1	2.62 (-1.07, 6.31)	0.164	—	—
BCI-visual vs shamBCI-visual	1	2.70 (-9.88, 15.28)	0.674	—	—
BCI-visual vs MI	1	7.10 (-7.34, 21.54)	0.335	—	—

Note: WMD > 0 favours the former intervention. I^2 and τ^2 were not estimated for single-study comparisons.

Table E6.2. Results of the node-splitting analysis

Comparison	Direct estimate	Indirect estimate	Difference	P value
BCI-MM vs UR	-3.928	-2.751	-1.177	0.778
BCI-MM vs FES	-7.840	-7.564	-0.276	1.000
BCI-MM vs MI	-1.061	-0.791	-0.270	0.964
BCI-MM vs Robot	1.800	0.066	1.734	0.718
BCI-MM vs shamBCI-MM	-1.294	-7.635	6.342	0.996
BCI-Robot vs UR	-5.685	-9.018	3.333	0.489
BCI-Robot vs MI	-3.700	-3.970	0.270	0.964
BCI-Robot vs Robot	-2.454	-0.481	-1.972	0.775
BCI-Robot vs shamBCI-Robot	-2.092	-13.678	11.586	0.998
Robot vs UR	-4.613	-3.854	-0.759	0.864

Note: Local inconsistencies were assessed using the node-splitting method. All p-values from the comparisons were > 0.05, indicating that no statistically significant local inconsistencies were found.

Table E6.3. Global inconsistency test

Test	χ^2	df	P value
Design-by-treatment interaction model	2.46	6	0.8728

Note: Global inconsistency was assessed using the design-by-treatment interaction model. The result showed no statistically significant inconsistency in the overall network.

Table E6.4. Loop-specific inconsistency analysis

Loop	Treatment loop	IF	95% CI	P value	τ^2
B-E-F	BCI-Robot–Robot–UR	4.02	0.00–15.71	0.500	12.371
A-B-D-E	BCI-MM–BCI-Robot–MI–Robot	2.08	0.00–14.80	0.749	6.199
A-B-D-F	BCI-MM–BCI-Robot–MI–UR	1.28	0.00–15.88	0.863	16.065
A-E-F	BCI-MM–Robot–UR	1.24	0.00–12.62	0.830	14.405

Note: IF, inconsistency factor; CI, confidence interval; τ^2, loop-specific heterogeneity. No closed loop showed statistically significant inconsistency. A = BCI-MM; B = BCI-Robot; C = FES; D = MI; E = Robot; F = UR; G = shamBCI-MM; H = shamBCI-Robot

Table E6.5. League table for FMA-UE.

WMD (95% CI)	BCI-FES	BCI-MM	BCI-Robot	BCI-visual	FES	MI	Robot	UR	shamBCI-MM	shamBCI-Robot	shamBCI-visual
**BCI-FES**	0										
**BCI-MM**	-1.4 (-8.9, 6.1)	0									
**BCI-Robot**	-3.7 (-11.4, 4.1)	-2.3 (-6.5, 2.0)	0								
**BCI-visual**	-7.3 (-25.1, 10.5)	-5.9 (-22.2, 10.4)	-3.7 (-20.2, 12.9)	0							
**FES**	7.9 (1.7, 14.1)	9.3 (-0.4, 19.1)	11.6 (1.6, 21.5)	15.2 (-3.7, 34.1)	0						
**MI**	-0.2 (-9.0, 8.5)	1.2 (-3.7, 6.0)	3.4 (-2.2, 9.1)	7.1 (-8.5, 22.7)	-8.1 (-18.9, 2.6)	0					
**Robot**	-1.6 (-9.5, 6.2)	-0.2 (-4.6, 4.1)	2.0 (-1.9, 5.9)	5.7 (-11, 22.4)	-9.5 (-19.6, 0.5)	-1.4 (-7.5, 4.6)	0				
**UR**	2.6 (-4.3, 9.5)	4.0 (1.0, 7.1)	6.3 (2.7, 9.9)	10.0 (-6.5, 26.4)	-5.3 (-14.5, 4.0)	2.9 (-2.5, 8.2)	4.3 (0.4, 8.1)	0			
**shamBCI-MM**	-0.14 (-9.3, 9.0)	1.3 (-4.0, 6.5)	3.5 (-3.2, 10.3)	7.2 (-9.9, 24.3)	-8.0 (-19.1, 3.0)	0.1 (-7.2, 7.0)	1.5 (-5.3, 8.3)	-2.8 (-8.8, 3.3)	0		
**shamBCI-Robot**	-2.8 (-11.7, 6.1)	-1.4 (-7.5, 4.8)	0.9 (-3.5, 5.2)	4.6 (-12.6, 21.7)	-10.7 (-21.5, 0.2)	-2.5 (-9.7, 4.6)	-1.1 (-7.0, 4.7)	-5.4 (-11.0, 0.2)	-2.6 (-10.7, 5.4)	0	
**shamBCI-visual**	-4.6 (-27.2, 17.9)	-3.2 (-24.6, 18.2)	-1.0 (-22.6, 20.6)	2.7 (-11.2, 16.6)	-12.5 (-35.9, 10.9)	-4.4 (-25.2, 16.4)	-3.0 (-24.7, 18.7)	-7.3 (-28.8, 14.3)	-4.5 (-26.5, 17.5)	-1.9 (-23.9, 20.2)	0

Notes: Values are WMDs with 95% CIs for the treatment in the column compared with the treatment in the row. WMD > 0 favours the column-defining treatment. Values are rounded to one decimal place. Statistically significant comparisons are shaded.

Appendix E7. Supplementary analyses for the ARAT outcome

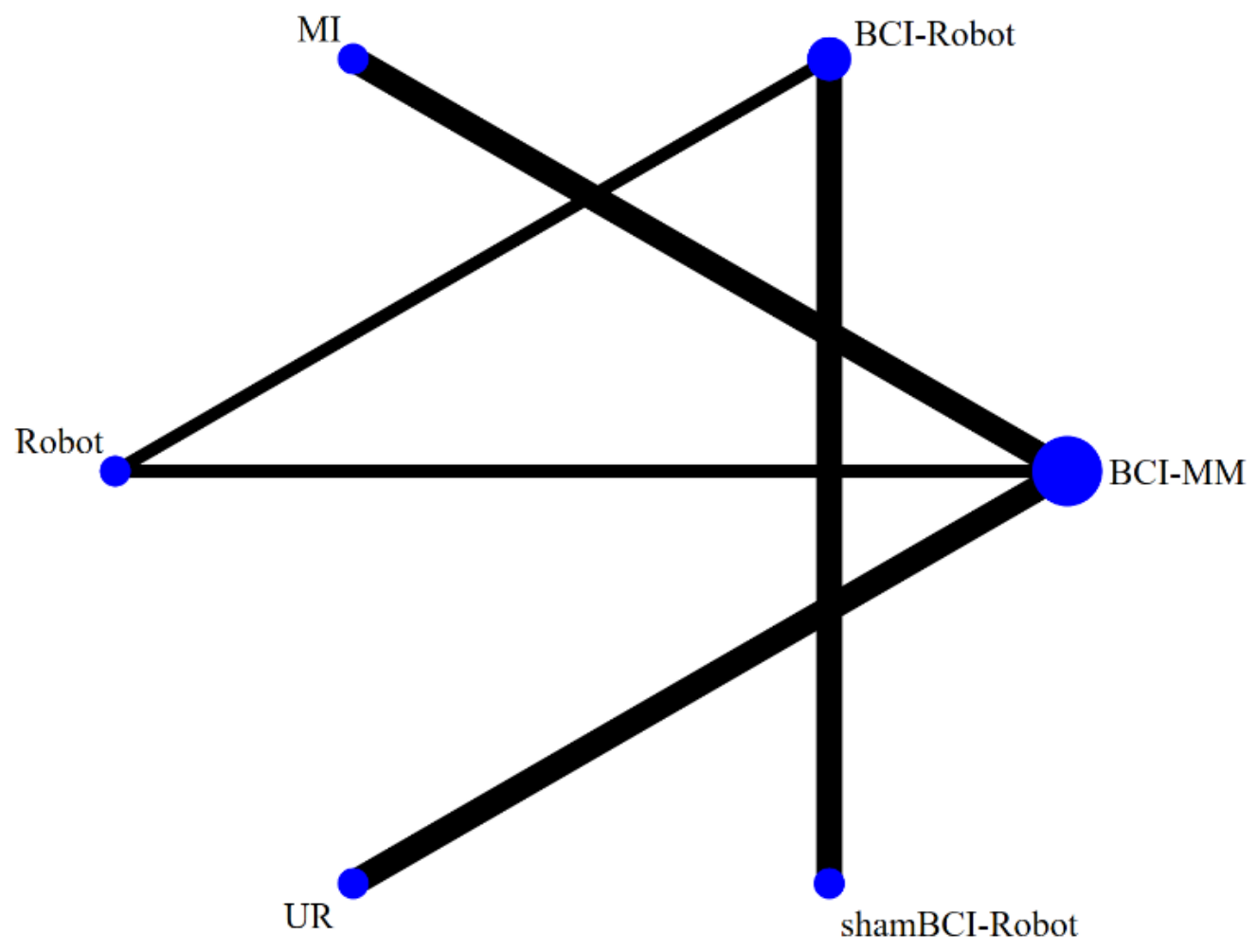

Figure E7.1. Network plot for the ARAT outcome

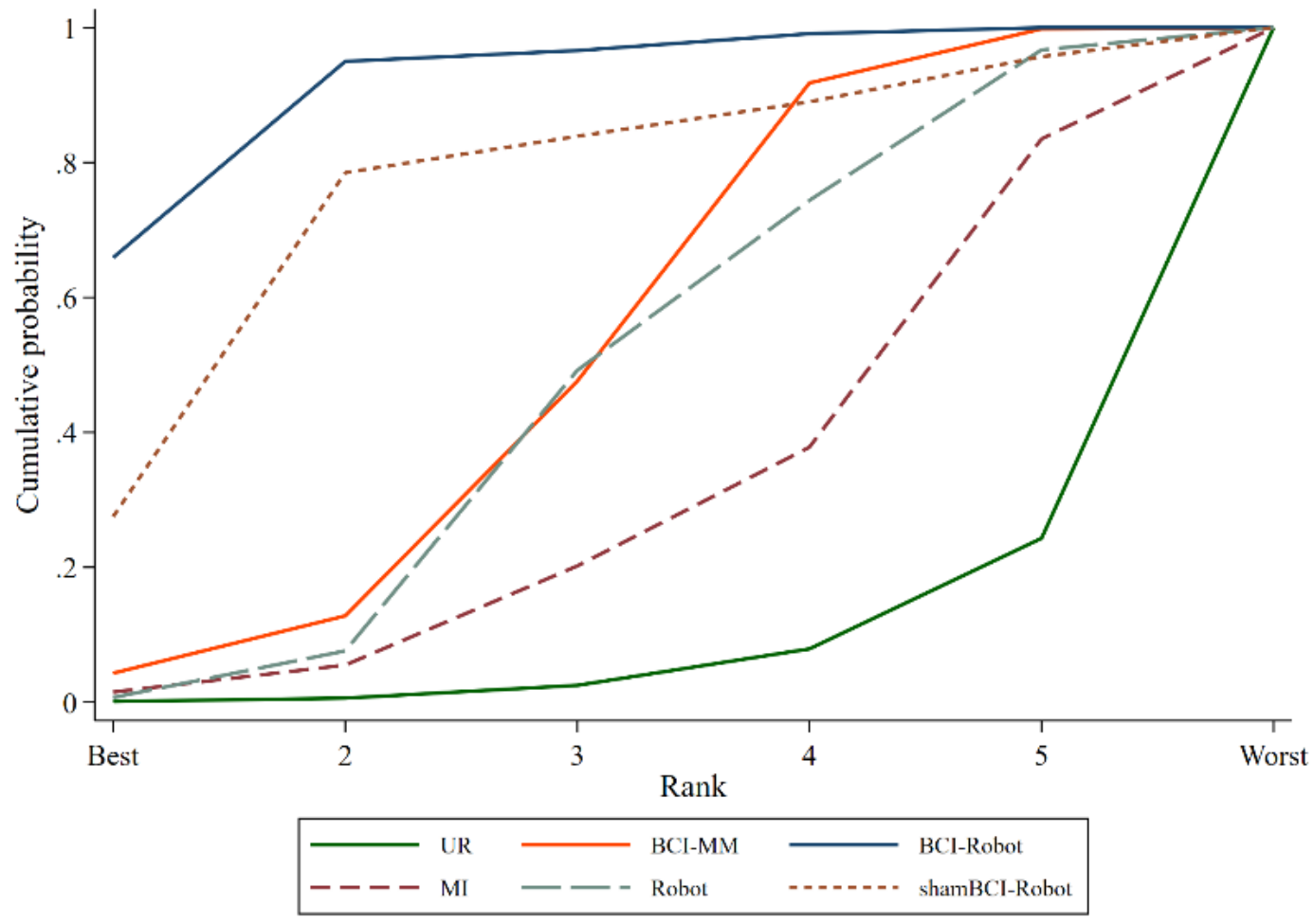

Figure E7.2. Cumulative ranking probability curves for ARAT

Table E7.1. Network estimates and treatment ranking compared with usual rehabilitation for ARAT.

Intervention	WMD vs UR (95% CI)	P value	SUCRA (%)	PrBest (%)	Mean rank
BCI-Robot	11.99 (1.34–22.65)	0.027	91.3	65.9	1.4
shamBCI-Robot	9.96 (−2.53–22.45)	0.118	74.9	27.5	2.3
BCI-MM	4.69 (−0.26–9.65)	0.063	51.3	4.3	3.4
Robot	4.49 (−1.61–10.60)	0.149	45.7	0.7	3.7
MI	3.17 (−3.17–9.51)	0.327	29.7	1.5	4.5
UR	Reference	—	7.1	0.1	5.6

Note: WMD > 0 favors the intervention over UR.

Table E7.2. League table for ARAT.

WMD (95% CI)	BCI-MM	BCI-Robot	MI	Robot	UR	shamBCI-Robot
BCI-MM	0					
BCI-Robot	−7.3 (−16.7–2.1)	0				
MI	1.5 (−1.9–4.9)	8.8 (−1.2–18.8)	0			
Robot	0.2 (−3.4–3.8)	7.5 (−1.2–16.2)	−1.3 (−6.2–3.6)	0		
UR	4.7 (−0.3–9.7)	**12.0 (1.3–22.7)**	3.2 (−3.2–9.5)	4.5 (−1.6–10.6)	0	
shamBCI-Robot	−5.3 (−16.8–6.3)	2.0 (−4.7–8.7)	−6.8 (−18.9–5.3)	−5.5 (−16.5–5.5)	−10.0 (−22.5–2.5)	0

Note: WMD > 0 favours the column-defining treatment. Values are rounded to one decimal place. Statistically significant comparisons are shown in bold.

Appendix E8. Supplementary analyses for the WMFT outcome

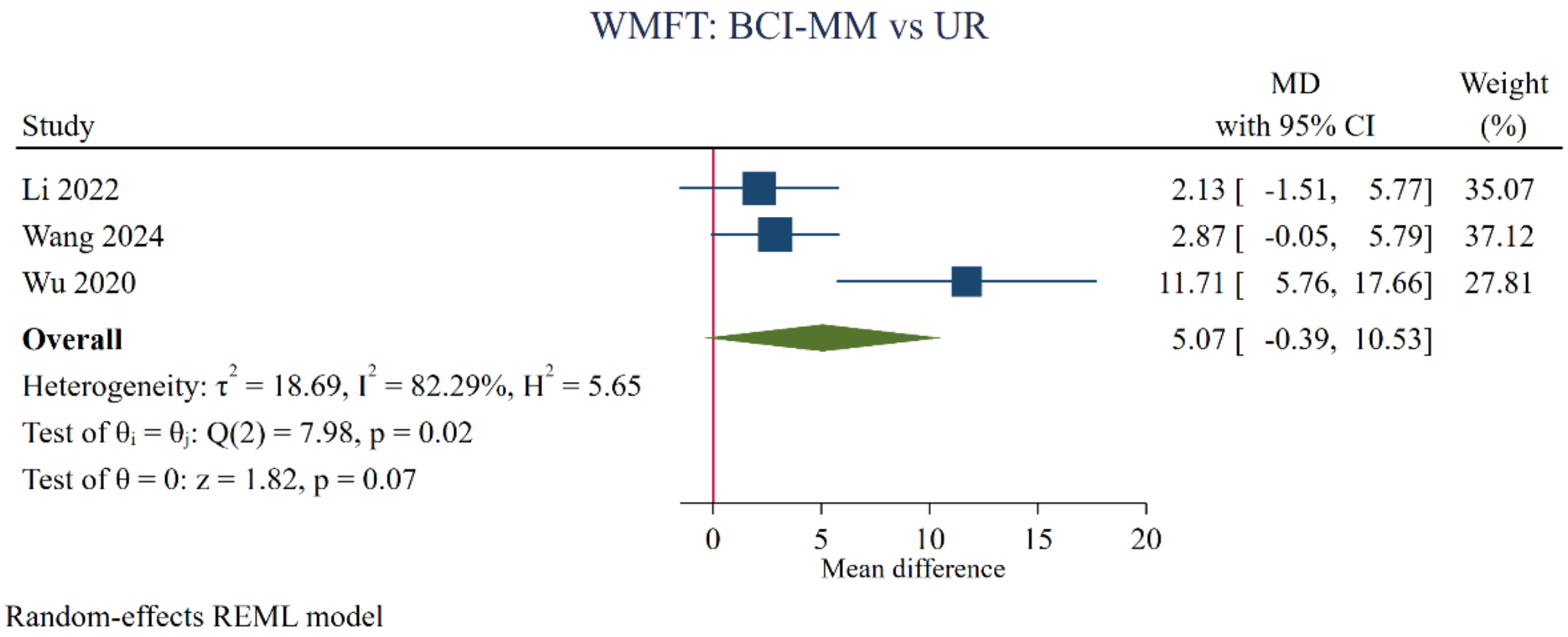

Figure E8.1. Forest plot of pairwise meta-analysis for BCI-MM versus usual rehabilitation on WMFT

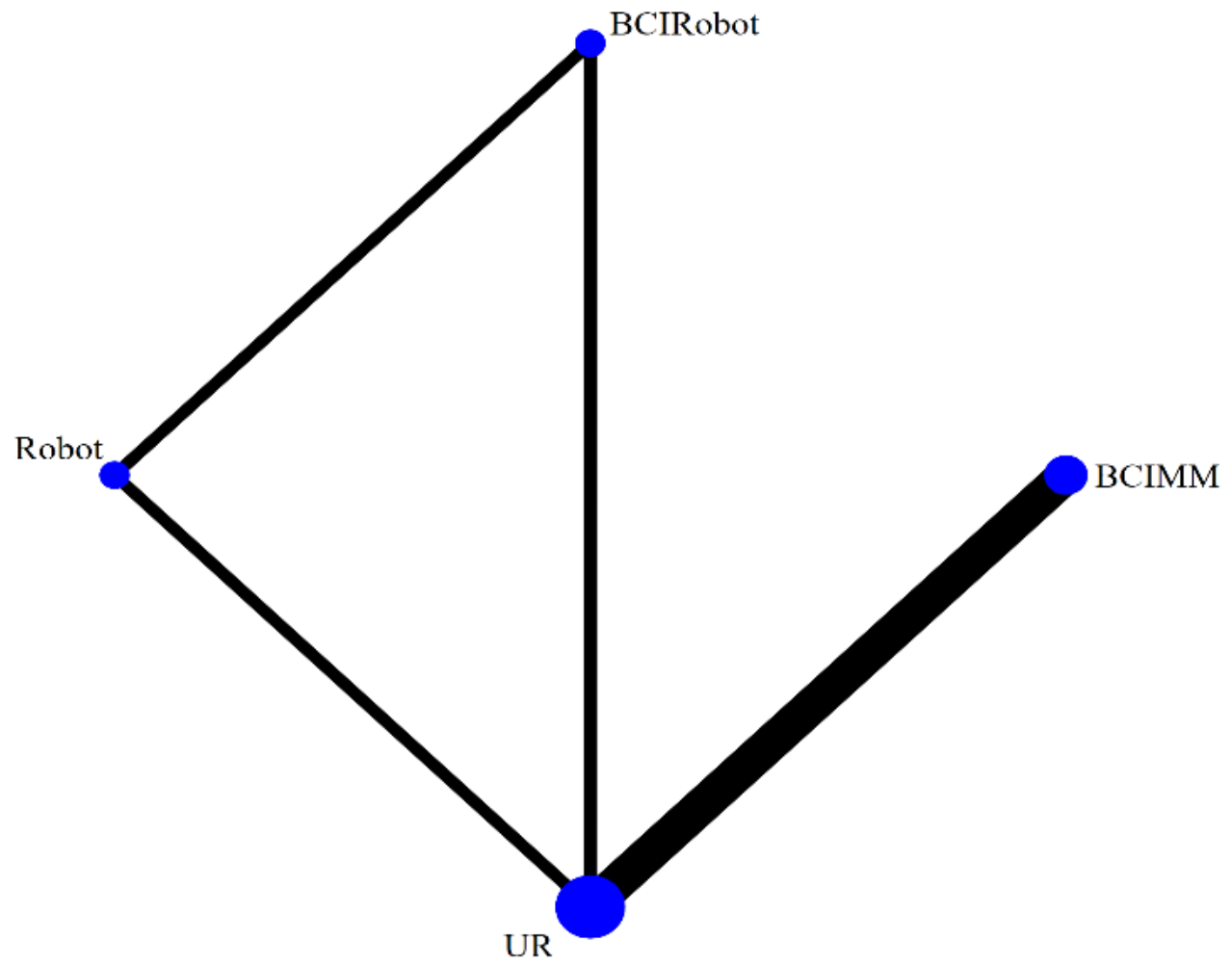

Figure E8.2. Exploratory network plot for the WMFT outcome

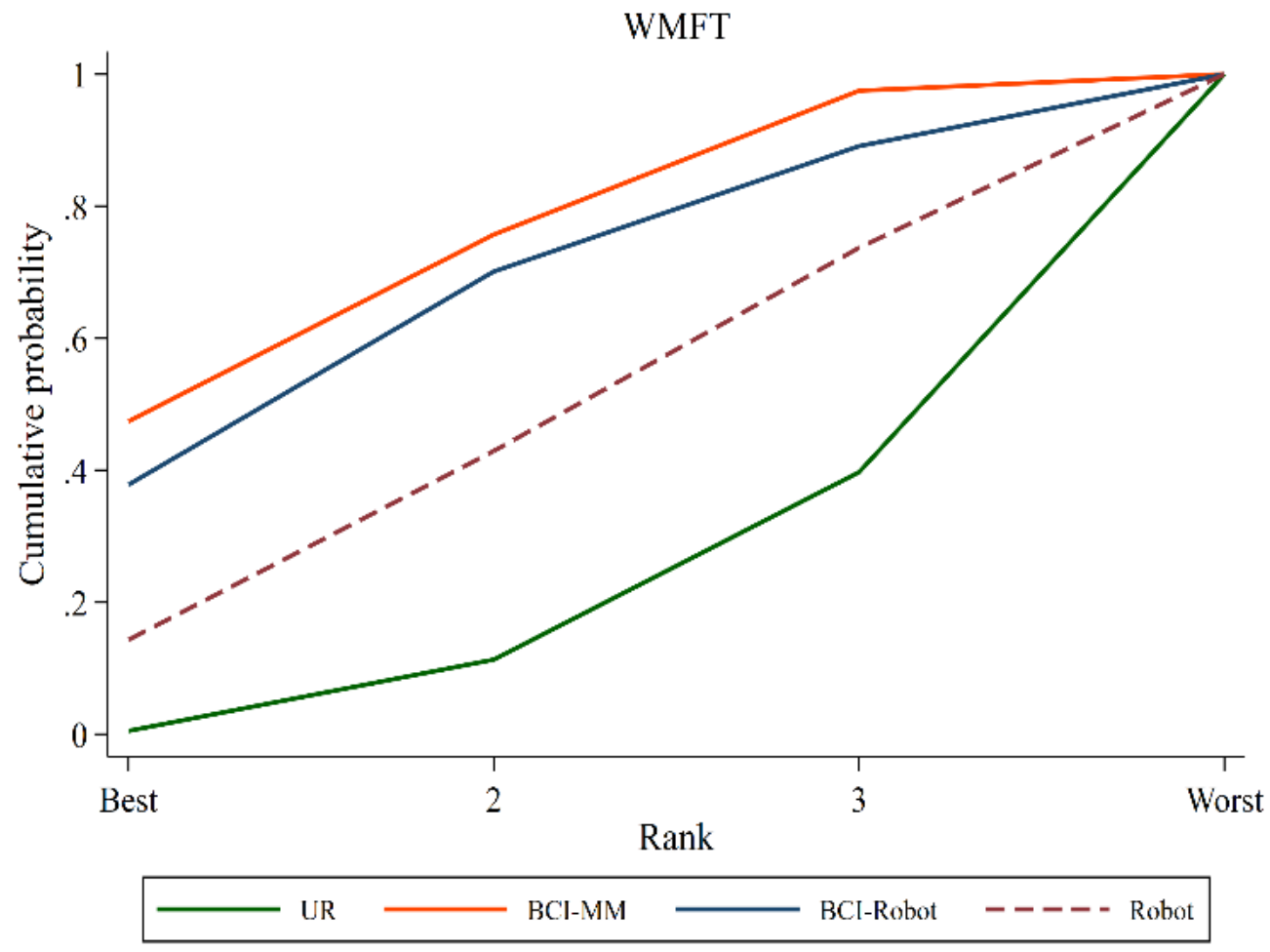

Figure E8.3. Cumulative ranking probability curves for WMFT

Table E8.1. Pairwise and exploratory network meta-analysis estimates for WMFT.

Analysis	Comparison	WMD (95% CI)	P value
Pairwise meta-analysis	BCI-MM vs UR	5.07 (−0.39–10.53)	0.069
Exploratory NMA	BCI-MM vs UR	5.07 (−0.48–10.62)	0.074
Exploratory NMA	BCI-Robot vs UR	4.40 (−4.76–13.56)	0.347
Exploratory NMA	Robot vs UR	2.20 (−6.46–10.86)	0.619

Note: WMFT was analyzed as the change from baseline, with higher values indicating greater improvement in upper-limb motor function. The pairwise meta-analysis of BCI-MM versus UR was considered the primary quantitative analysis, whereas the network meta-analysis was exploratory because of the sparse evidence network.

Table E8.2. Exploratory treatment ranking for WMFT.

Intervention	SUCRA (%)	PrBest (%)	Mean rank
BCI-MM	73.5	47.4	1.8
BCI-Robot	65.7	37.8	2.0
Robot	43.6	14.3	2.7
UR	17.2	0.5	3.5

Appendix E9. Subgroup analysis by stroke stage

Figure E9.1. Network plots stratified by stroke phase

A: Subacute phase; B: Chronic phase.

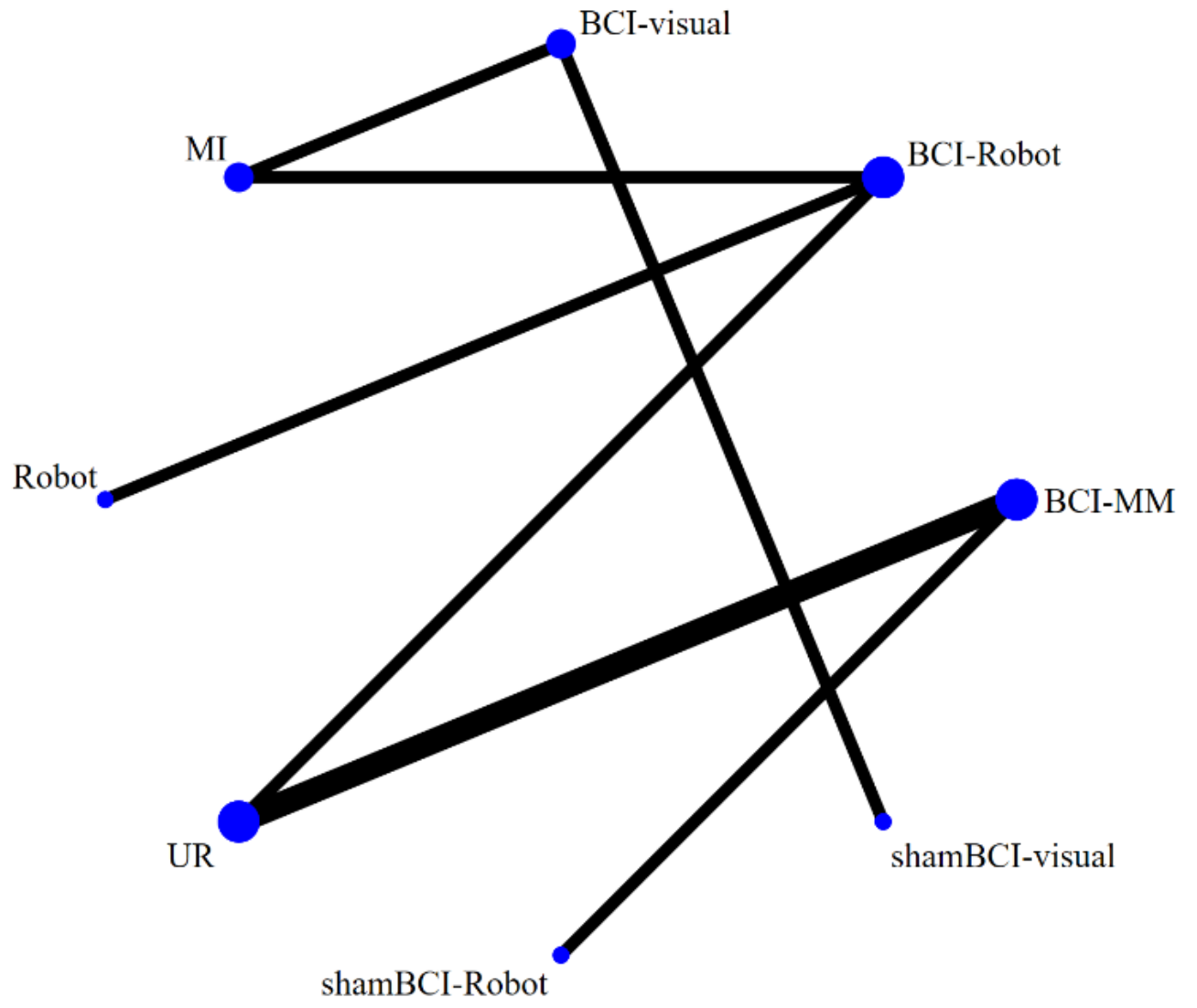

A: subacute subgroup

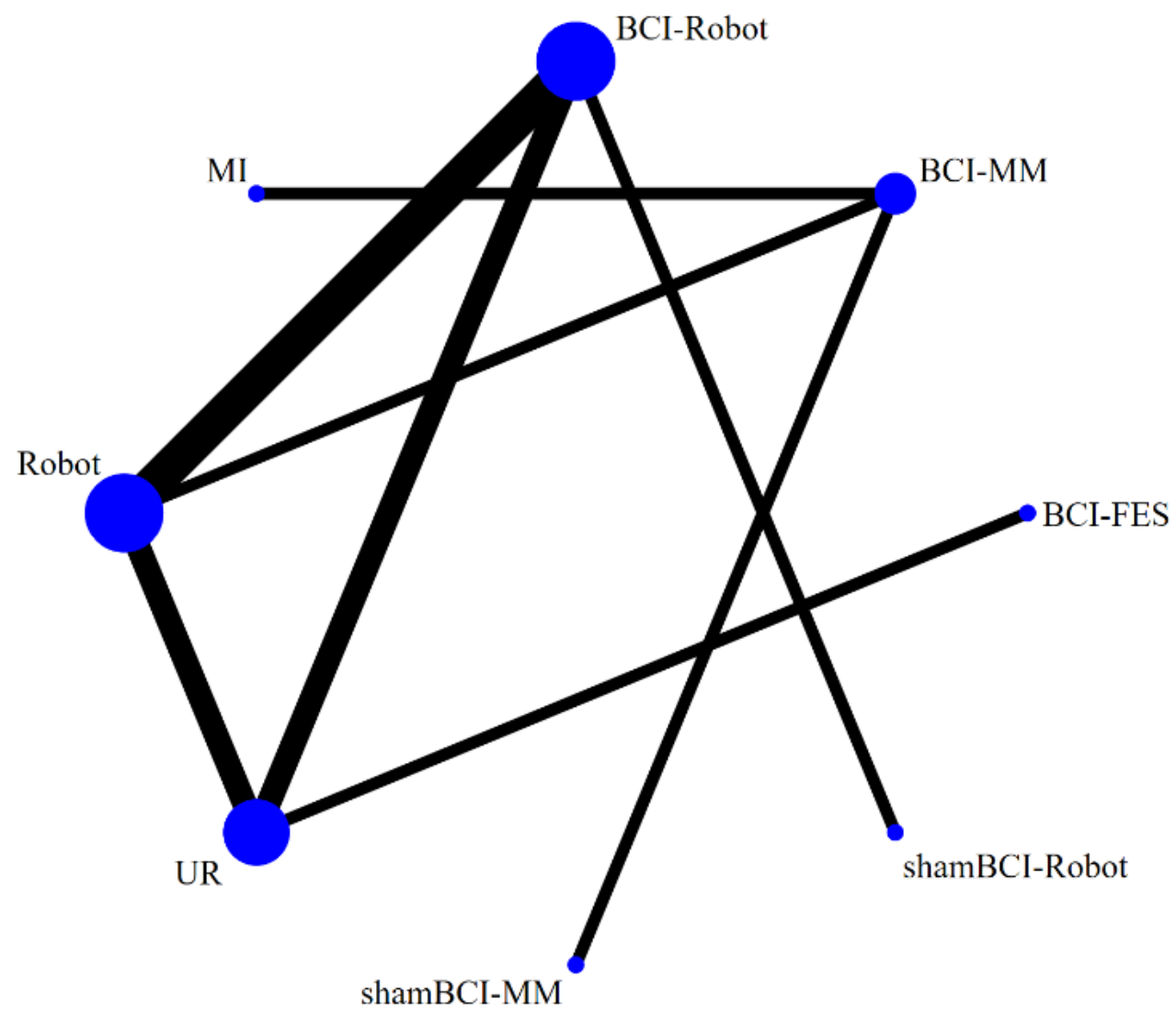

B: chronic subgroup

Figure E9.2. Forest plot of network estimates versus UR

A: Subacute phase; B: Chronic phase.

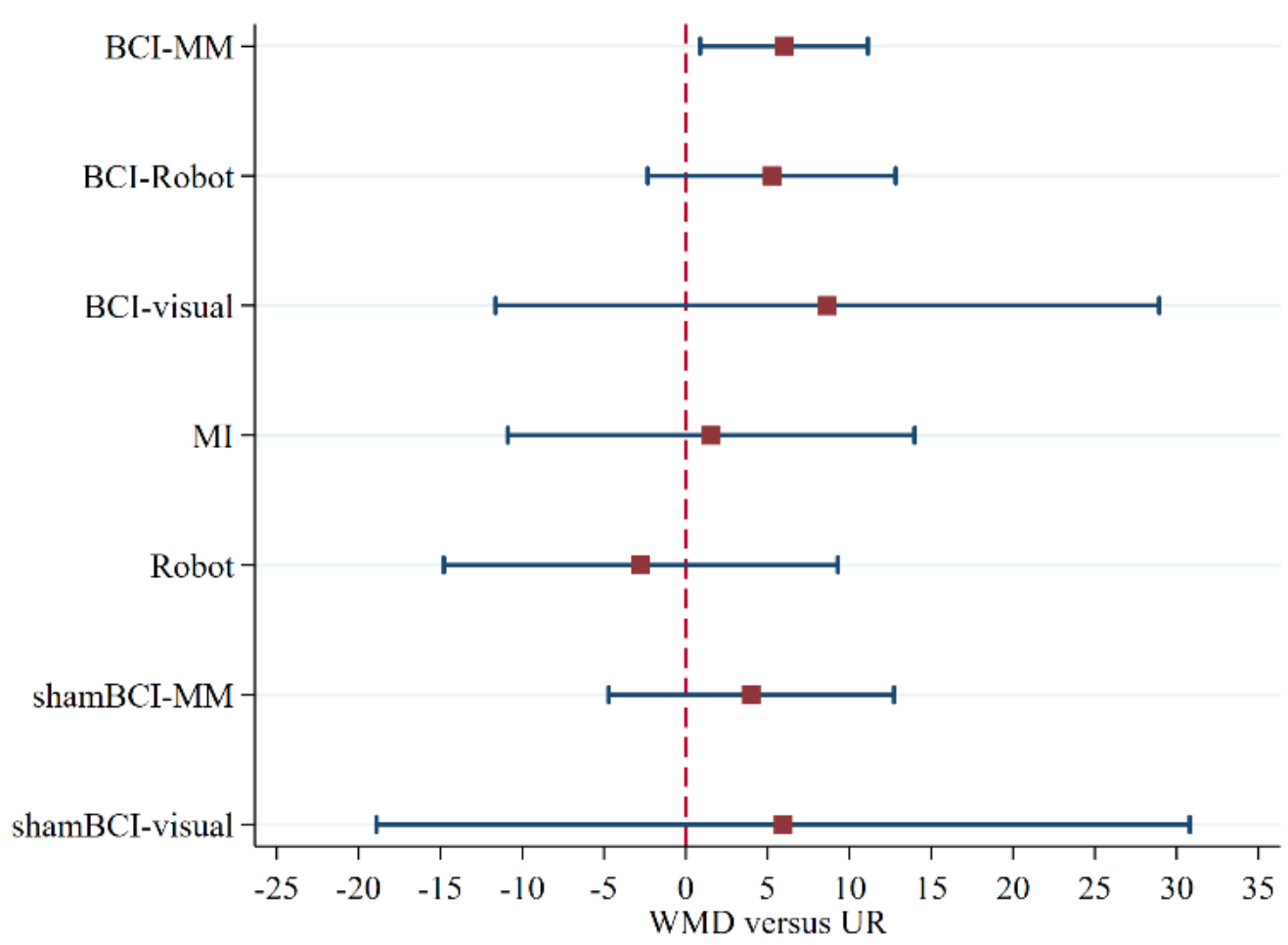

A: subacute subgroup

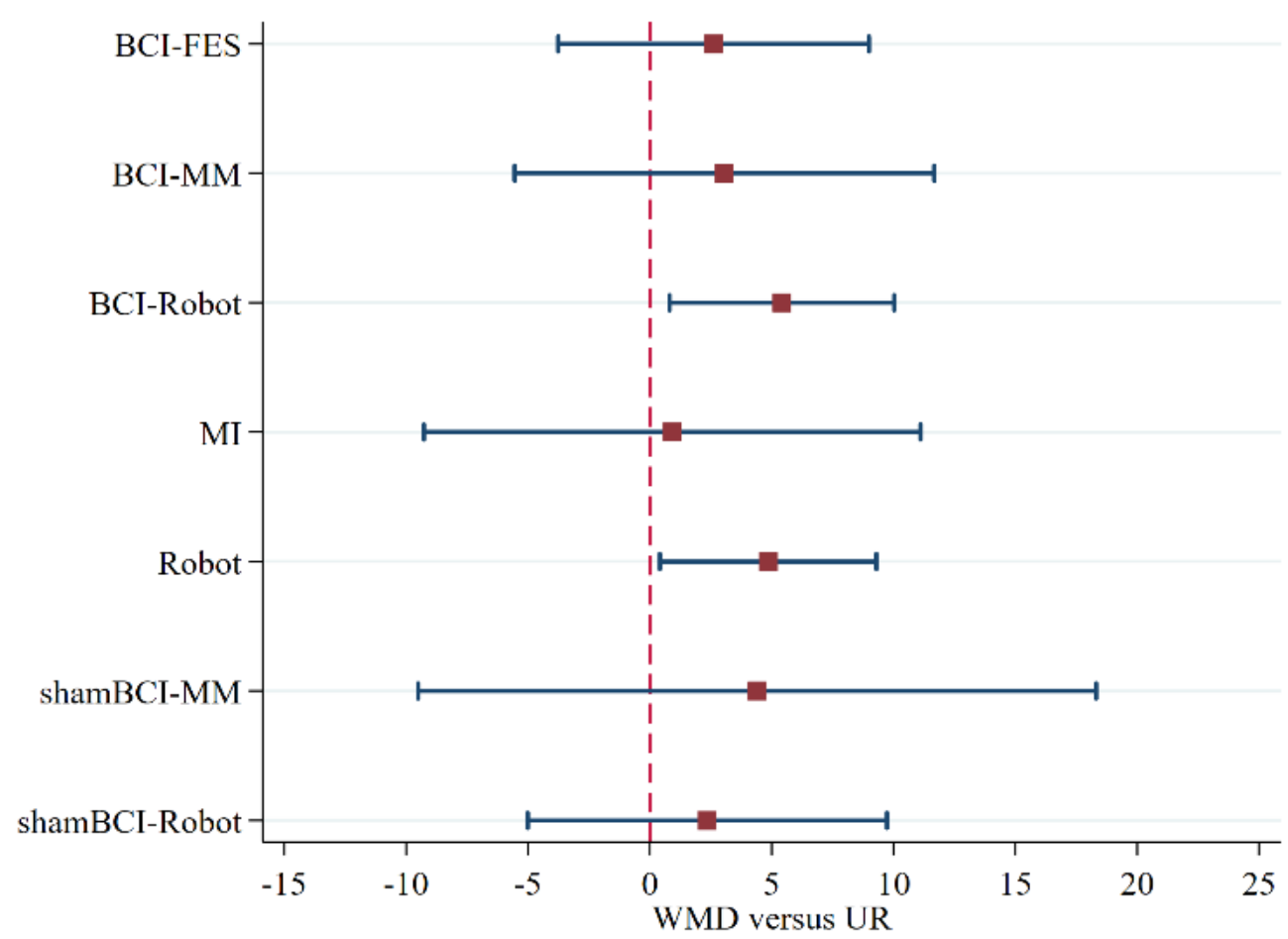

B: chronic subgroup

Figure E9.3. Cumulative ranking probability curves for FMA-UE stratified by stroke phase

A: Subacute phase; B: Chronic phase.

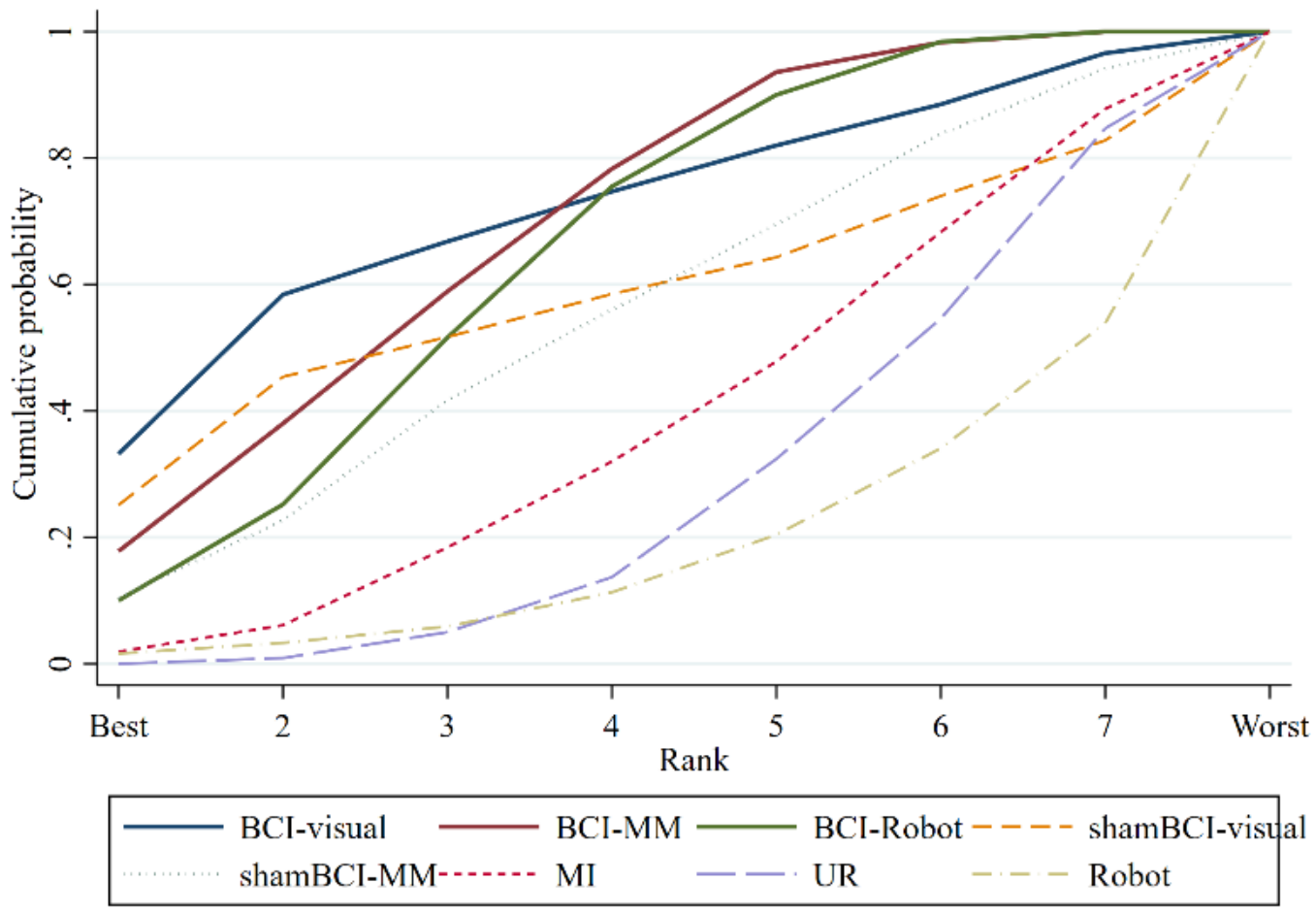

A: subacute subgroup

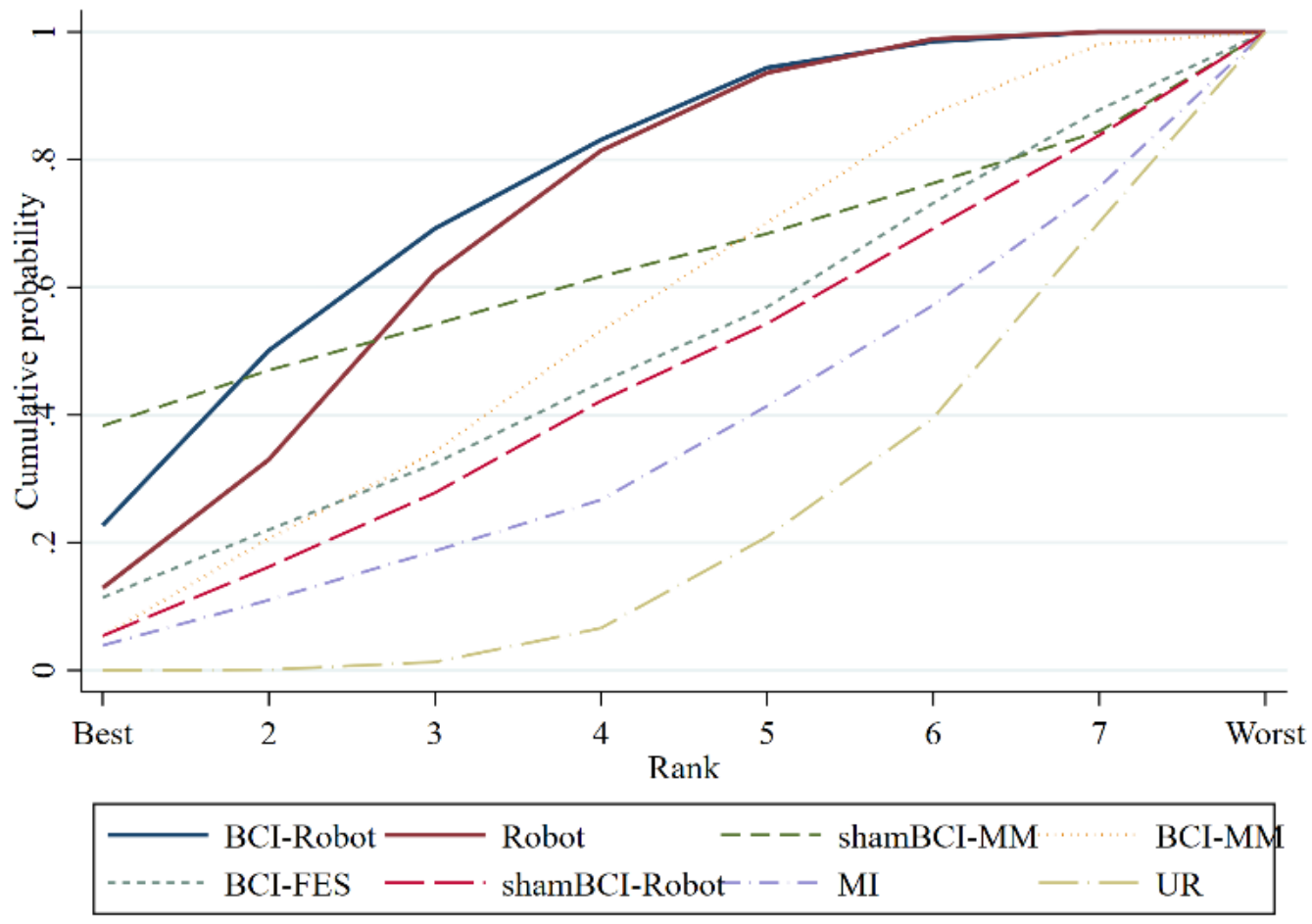

B: chronic subgroup

Table E9.1. Main network estimates and treatment rankings stratified by stroke phase

Stroke phase	Intervention	WMD vs UR (95% CI)	SUCRA	PrBest (%)	Mean rank
Subacute	BCI-visual	8.65 (-11.63, 28.93)	71.5	33.2	3.0
	BCI-MM	6.00 (0.88, 11.13)	69.3	17.8	3.2
	BCI-Robot	5.25 (-2.33, 12.83)	64.4	10.0	3.5
	shamBCI-visual	5.95 (-18.90, 30.80)	57.4	25.1	4.0
	shamBCI-MM	4.00 (-4.72, 12.72)	54.0	10.4	4.2
	MI	1.55 (-10.88, 13.98)	37.5	1.9	5.4
	UR	Reference	27.3	0.0	6.1
	Robot	-2.75 (-14.79, 9.29)	18.6	1.6	6.7
Chronic	BCI-Robot	5.42 (0.81, 10.03)	74.0	22.7	2.8
	Robot	4.86 (0.41, 9.30)	68.9	12.9	3.2
	shamBCI-MM	4.41 (-9.51, 18.32)	61.5	38.3	3.7
	BCI-MM	3.06 (-5.55, 11.67)	52.7	5.4	4.3
	BCI-FES	2.62 (-3.76, 9.00)	47.0	11.4	4.7
	shamBCI-Robot	2.36 (-5.01, 9.73)	42.7	5.4	5.0
	MI	0.92 (-9.27, 11.11)	33.5	3.9	5.7
	UR	Reference	19.8	0.0	6.6

Notes: WMD > 0 indicates that the intervention is superior to UR. Higher FMA-UE scores indicate better upper-limb motor function. Treatments were ordered by SUCRA within each subgroup. SUCRA, surface under the cumulative ranking curve; PrBest, probability of being ranked best. Subacute stroke was defined as 7 days to 6 months after stroke onset, and chronic stroke was defined as >6 months after stroke onset.

Table E9.2. League table of network meta-analysis in the subacute subgroup

WMD(95 % CI)	BCI-MM	BCI-Robot	BCI-visual	MI	Robot	UR	shamBCI-MM	shamBCI-visual
**BCI-MM**	0							
**BCI-Robot**	0.75 (-8.39,9.90)	0						
**BCI-visual**	-2.65 (-23.56,18.27)	-3.40 (-22.21,15.41)	0					
**MI**	4.45 (-8.99,17.90)	3.70 (-6.15,13.55)	7.10 (-8.92,23.12)	0				
**Robot**	8.75 (-4.33,21.84)	8.00 (-1.36,17.36)	11.40 (-9.61,32.41)	4.30 (-9.29,17.89)	0			
**UR**	6.00 (0.88,11.13)	5.25 (-2.33,12.83)	8.65 (-11.63,28.93)	1.55 (-10.88,13.98)	-2.75 (-14.79,9.29)	0		
**shamBCI-MM**	2.00 (-5.06,9.06)	1.25 (-10.31,12.80)	4.65 (-17.43,26.72)	-2.45 (-17.64,12.73)	-6.75 (-21.62,8.12)	-4.00 (-12.72,4.72)	0	
**shamBCI-visual**	0.05 (-25.32,25.43)	-0.70 (-24.37,22.97)	2.70 (-11.67,17.07)	-4.40 (-25.92,17.12)	-8.70 (-34.15,16.75)	-5.95 (-30.80,18.90)	-1.95 (-28.29,24.39)	0

Note: Values are WMDs with 95% CIs. Each cell represents the column-defining treatment compared with the row-defining treatment. Positive values favor the column-defining treatment.

Table E9.3. League table of network meta-analysis in the chronic subgroup

WMD(95 % CI)	BCI-FES	BCI-MM	BCI-Robot	MI	Robot	UR	shamBCI-MM	shamBCI-Robot
BCI-FES	0							
BCI-MM	-0.44 (-11.15,10.28)	0						
BCI-Robot	-2.80 (-10.67,5.07)	-2.36 (-11.10,6.37)	0					
MI	1.70 (-10.32,13.72)	2.14 (-3.31,7.59)	4.50 (-5.79,14.80)	0				
Robot	-2.24 (-10.01,5.54)	-1.80 (-9.17,5.57)	0.56 (-4.12,5.24)	-3.94 (-13.11,5.23)	0			
UR	2.62 (-3.76,9.00)	3.06 (-5.55,11.67)	5.42 (0.81,10.03)	0.92 (-9.27,11.11)	4.86 (0.41,9.30)	0		
shamBCI-MM	-1.79 (-17.10,13.52)	-1.35 (-12.28,9.58)	1.01 (-12.98,15.01)	-3.49 (-15.71,8.73)	0.45 (-12.74,13.64)	-4.41 (-18.32,9.51)	0	
shamBCI-Robot	0.26 (-9.48,10.01)	0.70 (-9.76,11.15)	3.06 (-2.68,8.80)	-1.44 (-13.23,10.35)	2.50 (-4.91,9.91)	-2.36 (-9.73,5.01)	2.05 (-13.08,17.17)	0

Note: Values are WMDs with 95% CIs. Each cell represents the column-defining treatment compared with the row-defining treatment. Positive values favor the column-defining treatment.

Appendix E10. Sensitivity analysis excluding studies with mixed stroke stages

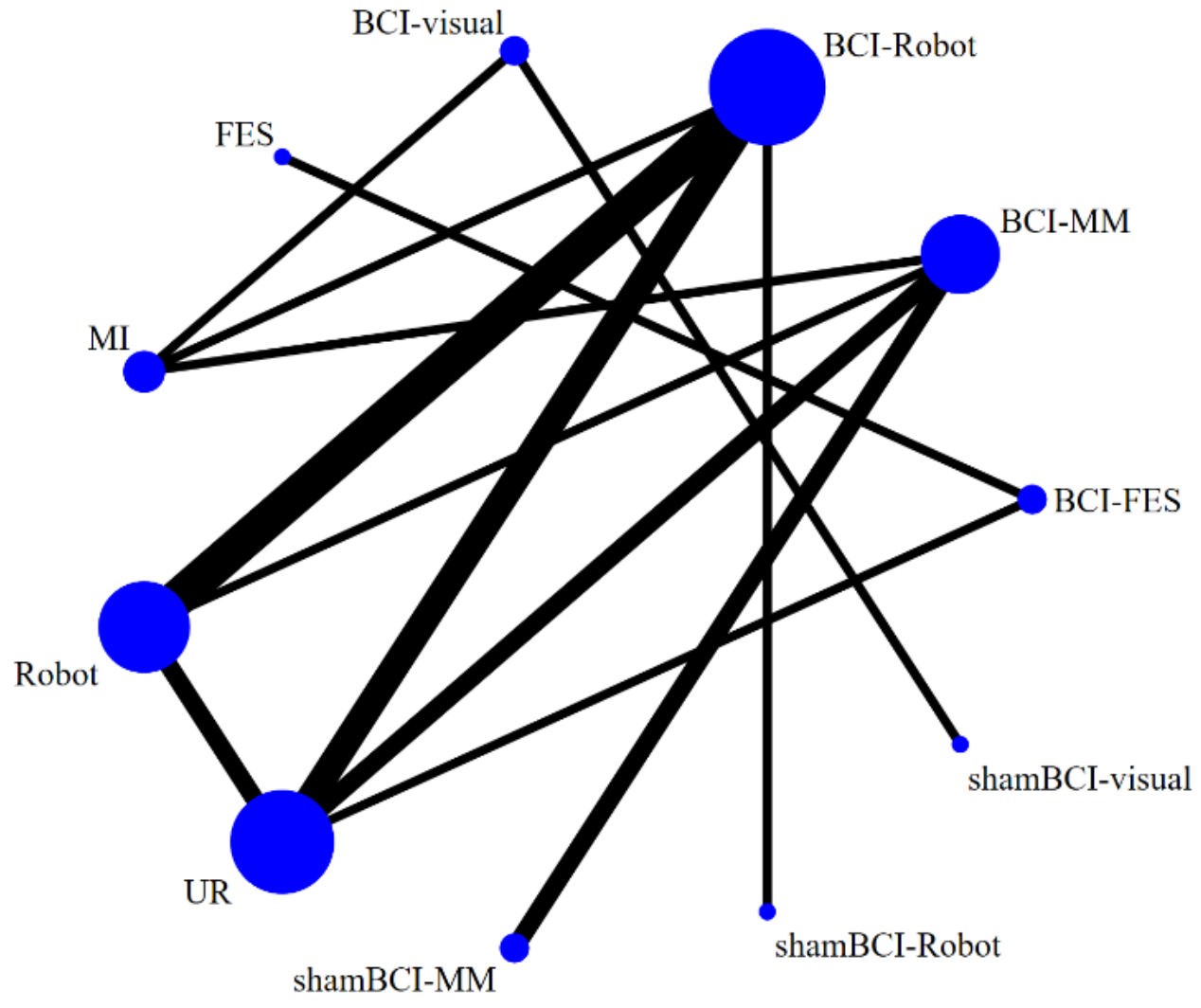

Figure E10.1. Network plot after excluding studies with mixed stroke stages

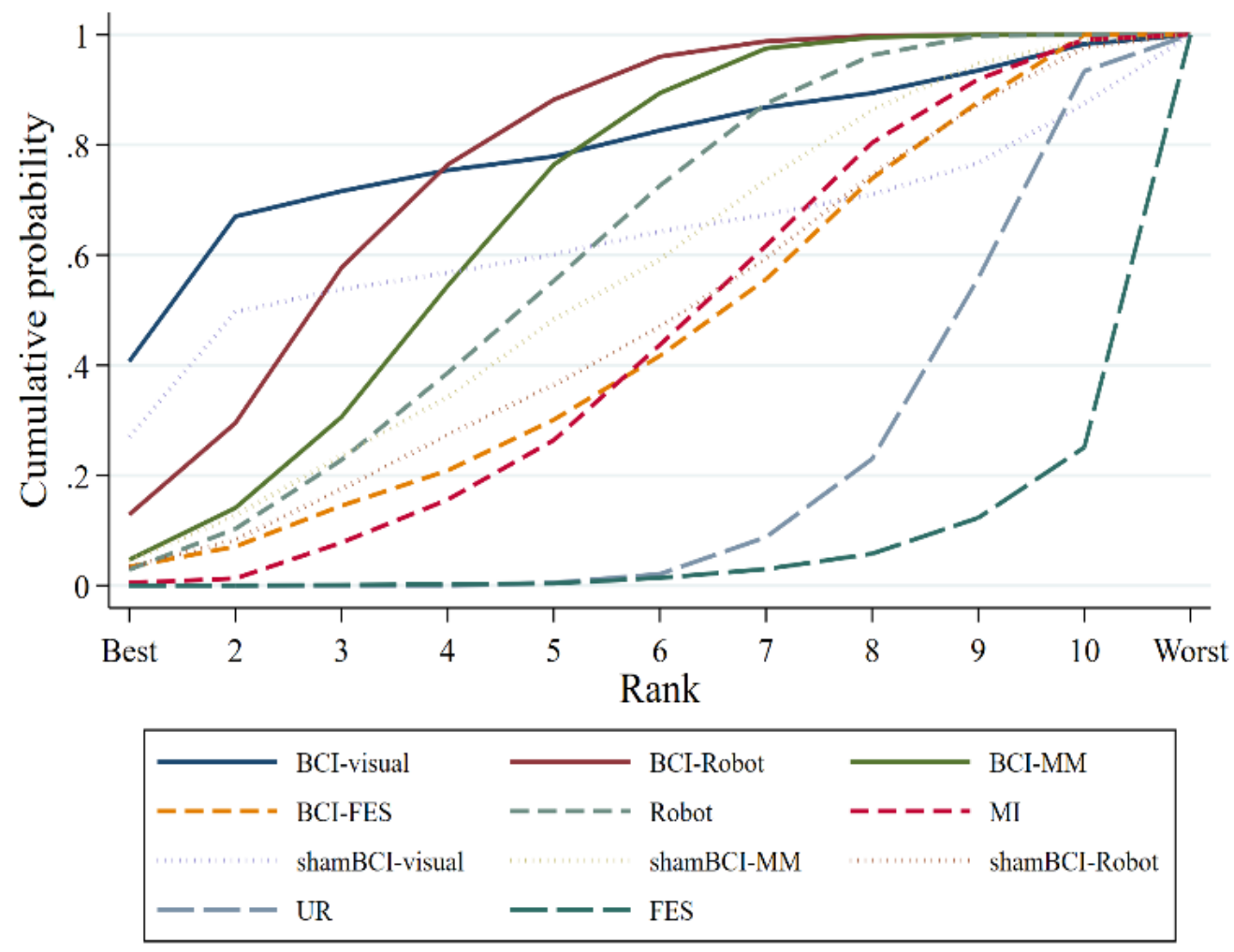

Figure E10.2. Cumulative ranking probability curves after excluding studies with mixed stroke stages

Table E10.1. Network estimates and treatment ranking after excluding studies with mixed stroke stages.

Intervention	WMD vs UR (95% CI)	P value	SUCRA (%)	PrBest (%)	Mean rank
BCI-FES	2.62 (−3.58, 8.82)	0.407	43.5	3.4	6.7
BCI-MM	5.28 (1.93, 8.62)	0.002	66.7	4.7	4.3
BCI-Robot	6.35 (3.08, 9.62)	<0.001	75.9	12.9	3.4
BCI-visual	10.11 (−6.04, 26.26)	0.220	78.3	40.7	3.2
FES	−5.28 (−13.54, 2.98)	0.210	4.8	0.0	10.5
MI	3.01 (−2.24, 8.25)	0.261	42.8	0.5	6.7
Robot	4.60 (1.06, 8.14)	0.011	58.6	2.9	5.1
shamBCI-MM	3.89 (−1.81, 9.60)	0.181	53.6	4.5	5.6
shamBCI-Robot	3.29 (−3.14, 9.72)	0.316	45.9	3.4	6.4
shamBCI-visual	7.41 (−13.66, 28.47)	0.491	61.4	27.0	4.9
UR	Reference	—	18.4	0.0	9.2

Note: WMD > 0 favours greater improvement in FMA-UE scores compared with UR. UR was used as the reference intervention. Higher SUCRA values indicate a higher probability of better treatment ranking; PrBest indicates the probability of being the best treatment.

Table E10.2 League table after excluding studies with mixed stroke stages

WMD (95%CI)	BCI-FES	BCI-MM	BCI-Robot	BCI-visual	FES	MI	Robot	UR	shamBCI-MM	shamBCI-Robot	shamBCI-visual
**BCI-FES**	0										
**BCI-MM**	-2.66 (-9.70,4.38)	0									
**BCI-Robot**	-3.73 (-10.74,3.28)	-1.07 (-5.31,3.16)	0								
**BCI-visual**	-7.49 (-24.78,9.81)	-4.83 (-20.78,11.13)	-3.76 (-19.97,12.46)	0							
**FES**	7.90 (2.44,13.36)	10.56 (1.64,19.47)	11.63 (2.75,20.52)	15.39 (-2.75,33.53)	0						
**MI**	-0.39 (-8.51,7.73)	2.27 (-2.34,6.88)	3.34 (-2.09,8.78)	7.10 (-8.17,22.37)	-8.29 (-18.07,1.50)	0					
**Robot**	-1.98 (-9.11,5.16)	0.68 (-3.50,4.86)	1.75 (-1.86,5.36)	5.51 (-10.80,21.82)	-9.88 (-18.87,-0.89)	-1.59 (-7.31,4.13)	0				
**UR**	2.62 (-3.58,8.82)	5.28 (1.93,8.62)	6.35 (3.08,9.62)	10.11 (-6.04,26.26)	-5.28 (-13.54,2.98)	3.01 (-2.24,8.25)	4.60 (1.06,8.14)	0			
**shamBCI-MM**	-1.27 (-9.70,7.15)	1.39 (-3.29,6.06)	2.46 (-3.79,8.70)	6.21 (-10.41,22.83)	-9.17 (-19.21,0.87)	-0.89 (-7.44,5.67)	0.70 (-5.56,6.97)	-3.89 (-9.60,1.81)	0		
**shamBCI-Robot**	-0.67 (-9.60,8.26)	1.99 (-4.99,8.96)	3.06 (-2.48,8.60)	6.82 (-10.32,23.95)	-8.57 (-19.04,1.90)	-0.28 (-8.05,7.48)	1.31 (-5.31,7.92)	-3.29 (-9.72,3.14)	0.60 (-7.75,8.95)	0	
**shamBCI-visual**	-4.79 (-26.75,17.17)	-2.13 (-23.05,18.79)	-1.06 (-22.17,20.06)	2.70 (-10.83,16.23)	-12.69 (-35.32,9.94)	-4.40 (-24.80,16.00)	-2.81 (-24.00,18.38)	-7.41 (-28.47,13.66)	-3.51 (-24.94,17.92)	-4.12 (-25.95,17.72)	0

Note: Each cell represents the effect of the column-defining intervention compared with the row-defining intervention. WMD > 0 favours the column intervention. Results were considered statistically significant when the 95% CI did not include 0.

Appendix E11. Sensitivity analyses using alternative pre–post correlation coefficients

Sensitivity analyses were performed using $r = 0.25$ and $r = 0.75$, compared with $r = 0.50$ in the primary analysis. Change-score standard deviations requiring derivation were recalculated accordingly, with all other data and analytical procedures unchanged.

Table E11.1. Network estimates and treatment rankings across alternative pre–post correlation coefficients

Intervention	WMD vs UR (95% CI)			SUCRA, (%)		
	r = 0.25	r = 0.5	r = 0.75	r = 0.25	r = 0.5	r = 0.75
BCI-FES	2.62 (−4.30, 9.54)	2.62 (−4.25, 9.49)	2.62 (−4.20, 9.44)	46.5	45.3	43.0
BCI-MM	3.95 (0.86, 7.04)	4.03 (1.00, 7.06)	4.17 (1.23, 7.11)	55.9	55.5	54.3
BCI-Robot	6.28 (2.67, 9.90)	6.28 (2.70, 9.86)	6.27 (2.74, 9.81)	77.0	76.3	74.9
BCI-visual	9.74 (−9.60, 29.07)	9.95 (−6.50, 26.41)	10.34 (−2.59, 23.28)	74.3	77.6	83.0
FES	−5.28 (−14.61, 4.05)	−5.28 (−14.54, 3.98)	−5.28 (−14.48, 3.92)	7.4	6.4	5.0
MI	2.64 (−2.84, 8.12)	2.85 (−2.51, 8.22)	3.24 (−1.96, 8.44)	42.8	43.0	44.1
Robot	4.17 (0.29, 8.06)	4.26 (0.42, 8.10)	4.42 (0.64, 8.20)	57.1	56.6	55.8
shamBCI-MM	2.53 (−3.74, 8.80)	2.76 (−3.30, 8.82)	3.18 (−2.55, 8.91)	44.3	45.0	46.4
shamBCI-Robot	5.42 (−0.31, 11.15)	5.39 (−0.24, 11.03)	5.32 (−0.17, 10.81)	65.8	64.7	62.3
shamBCI-visual	7.04 (−18.36, 32.44)	7.25 (−14.25, 28.76)	7.64 (−9.09, 24.37)	59.0	61.1	65.0
UR	Reference	Reference	Reference	20.0	18.4	16.1

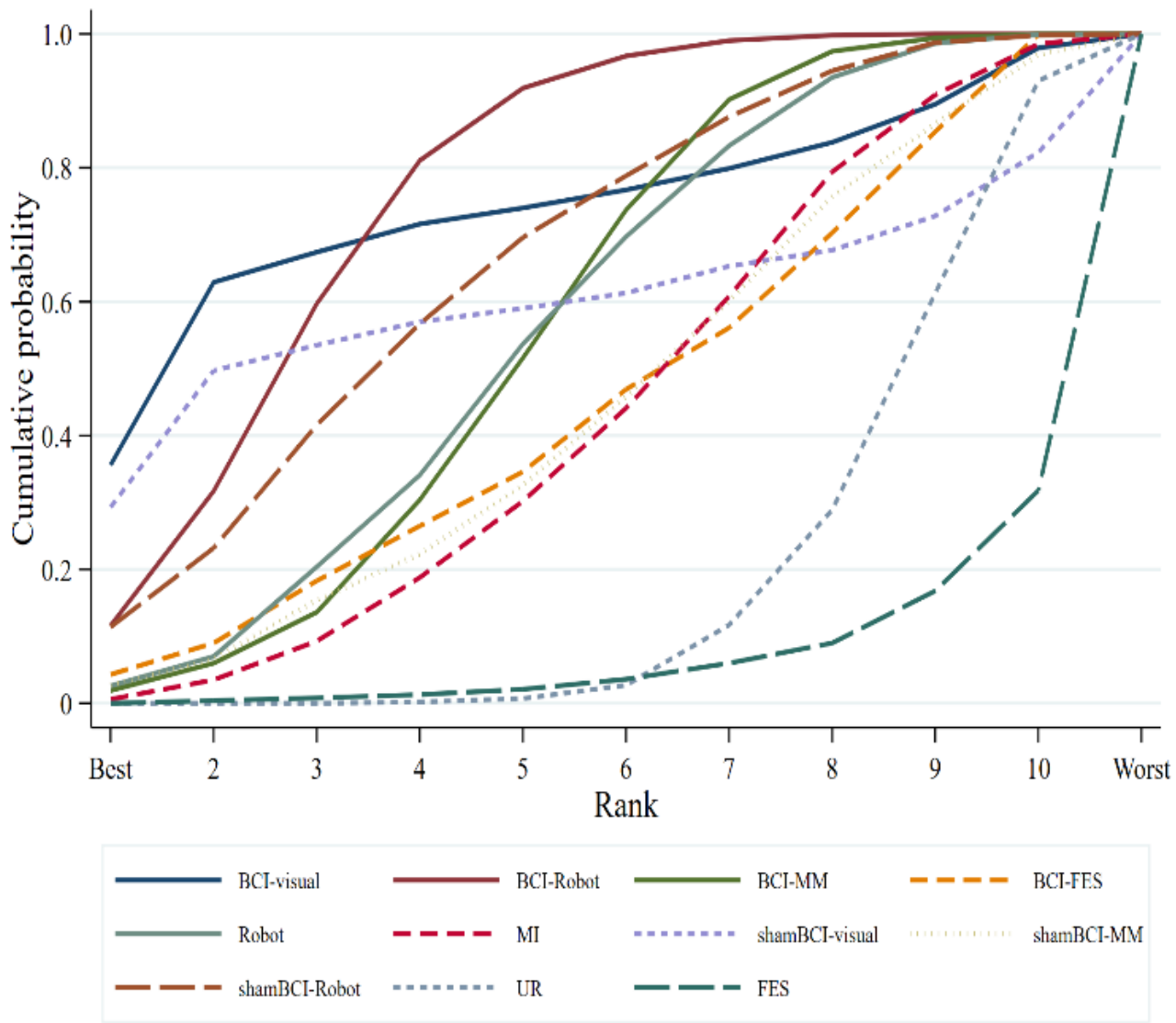

Figure E11.1. Cumulative ranking probability curves assuming r=0.25

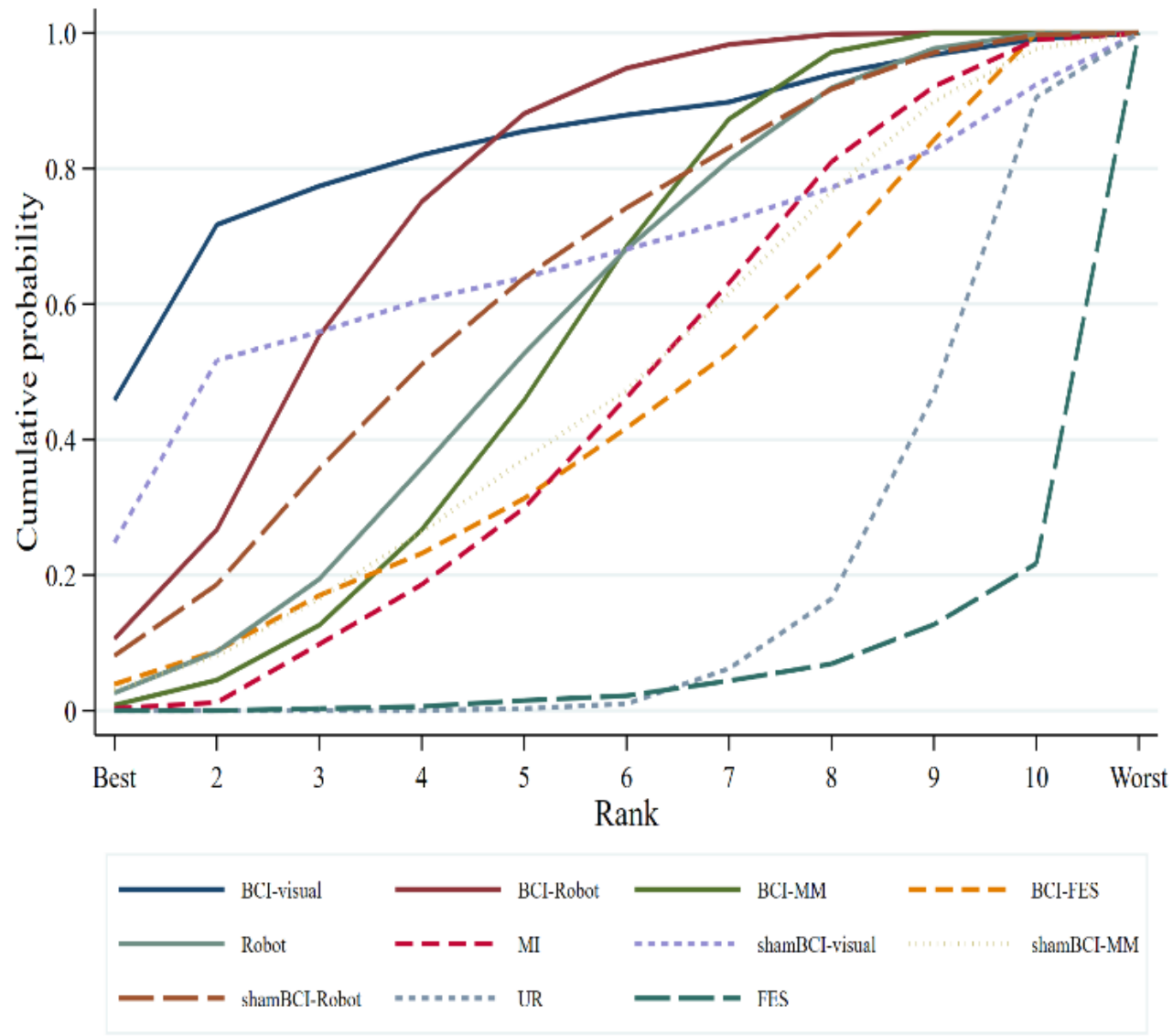

Figure E11.1. Cumulative ranking probability curves assuming r=0.75

Network estimates were similar across r = 0.25, r = 0.50, and r = 0.75. BCI-Robot, BCI-MM, and Robot remained significantly superior to UR under all three assumptions, whereas BCI-FES and BCI-visual remained non-significant. Treatment rankings were broadly consistent, although the SUCRA value for BCI-visual increased with higher r values. Overall, the main findings were unchanged across alternative correlation assumptions.

Appendix E12. References and analysis inclusion status of included studies

E12.1 References of included randomized controlled trials

(1) Ang KK, Chua KS, Phua KS, Wang C, Chin ZY, Kuah CW, Low W, Guan C. A Randomized Controlled Trial of EEG-Based Motor Imagery Brain-Computer Interface Robotic Rehabilitation for Stroke. Clin EEG Neurosci. 2015 Oct;46(4):310-20. doi: 10.1177/1550059414522229. Epub 2014 Apr 21. PMID: 24756025.

(2) Ang KK, Guan C, Phua KS, Wang C, Zhou L, Tang KY, Ephraim Joseph GJ, Kuah CW, Chua KS. Brain-computer interface-based robotic end effector system for wrist and hand rehabilitation: results of a three-armed randomized controlled trial for chronic stroke. Front Neuroeng. 2014 Jul 29;7:30. doi: 10.3389/fneng.2014.00030. PMID: 25120465; PMCID: PMC4114185.

(3) Cantillo-Negrete J, Rodríguez-García ME, Carrillo-Mora P, Arias-Carrión O, Ortega-Robles E, Galicia-Alvarado MA, Valdés-Cristerna R, Ramirez-Nava AG, Hernandez-Arenas C, Quinzaños-Fresnedo J, Pacheco-Gallegos MDR, Marín-Arriaga N, Carino-Escobar RI. The ReHand-BCI trial: a randomized controlled trial of a brain-computer interface for upper extremity stroke neurorehabilitation. Front Neurosci. 2025 Jun 18;19:1579988. doi: 10.3389/fnins.2025.1579988. PMID: 40606836; PMCID: PMC12216668.

(4) Chen S, Cao L, Shu X, Wang H, Ding L, Wang SH, Jia J. Longitudinal Electroencephalography Analysis in Subacute Stroke Patients During Intervention of Brain-Computer Interface With Exoskeleton Feedback. Front Neurosci. 2020 Aug 14;14:809. doi: 10.3389/fnins.2020.00809. PMID: 32922254; PMCID: PMC7457033.

(5) Cheng N, Phua KS, Lai HS, Tam PK, Tang KY, Cheng KK, Yeow RC, Ang KK, Guan C, Lim JH. Brain-Computer Interface-Based Soft Robotic Glove Rehabilitation for Stroke. IEEE Trans Biomed Eng. 2020 Dec;67(12):3339-3351. doi: 10.1109/TBME.2020.2984003. Epub 2020 Nov 19. PMID: 32248089.

(6) Frolov AA, Mokienko O, Lyukmanov R, Biryukova E, Kotov S, Turbina L, Nadareyshvily G, Bushkova Y. Post-stroke Rehabilitation Training with a Motor-Imagery-Based Brain-Computer Interface (BCI)-Controlled Hand Exoskeleton: A Randomized Controlled Multicenter Trial. Front Neurosci. 2017 Jul 20;11:400. doi: 10.3389/fnins.2017.00400. PMID: 28775677; PMCID: PMC5517482.

(7) Fu J, Chen S, Shu X, Lin Y, Jiang Z, Wei D, Gao J, Jia J. Functional-oriented, portable brain-computer interface training for hand motor recovery after stroke: a randomized controlled study. Front Neurosci. 2023 May 11;17:1146146. doi: 10.3389/fnins.2023.1146146. PMID: 37250399; PMCID: PMC10213744.

(8) Guo N, Wang X, Duanmu D, Huang X, Li X, Fan Y, Li H, Liu Y, Yeung EHK, To MKT, Gu J, Wan F, Hu Y. SSVEP-Based Brain Computer Interface Controlled Soft Robotic Glove for Post-Stroke Hand Function Rehabilitation. IEEE Trans Neural Syst Rehabil Eng. 2022;30:1737-1744. doi: 10.1109/TNSRE.2022.3185262. Epub 2022 Jul 4. PMID: 35731756.

(9) He J, Yuan Z, Quan L, Xi H, Guo J, Zhu D, Chen M, Yang B, Cui Z, Zhu S, Qiao J. Multimodal assessment of a BCI system for stroke rehabilitation integrating motor imagery and motor attempts: a randomized controlled trial. J Neuroeng Rehabil. 2025 Aug 26;22(1):185. doi: 10.1186/s12984-025-01723-8. PMID: 40859358; PMCID: PMC12379318.

(10) Hu YQ, Gao TH, Li J, Tao JC, Bai YL, Lu RR. Motor Imagery-Based Brain-Computer Interface Combined with Multimodal Feedback to Promote Upper Limb Motor Function after Stroke: A Preliminary Study. Evid Based Complement Alternat Med. 2021 Nov 3;2021:1116126. doi: 10.1155/2021/1116126. PMID: 34777531; PMCID: PMC8580676.

(11) Ji X, Lu X, Xu Y, Zhang W, Yang H, Yin C, Wang H, Ren C, Ji Y, Li Y, Huang G, Shen Y. Effects and neural mechanisms of a brain-computer interface-controlled soft robotic glove on upper limb function in patients with subacute stroke: a randomized controlled fNIRS study. J Neuroeng Rehabil. 2025 Jul 24;22(1):171. doi: 10.1186/s12984-025-01704-x. PMID:

40707971; PMCID: PMC12288246.
(12) Kim MS, Park H, Kwon I, An KO, Kim H, Park G, Hyung W, Im CH, Shin JH. Efficacy of brain-computer interface training with motor imagery-contingent feedback in improving upper limb function and neuroplasticity among persons with chronic stroke: a double-blinded, parallel-group, randomized controlled trial. J Neuroeng Rehabil. 2025 Jan 6;22(1):1. doi: 10.1186/s12984-024-01535-2. PMID: 39757218; PMCID: PMC11702034.
(13) Lu R, Pang Z, Gao T, He Z, Hu Y, Zhuang J, Zhang Q, Gao Z. Multisensory BCI promotes motor recovery via high-order network-mediated interhemispheric integration in chronic stroke. BMC Med. 2025 Jul 1;23(1):380. doi: 10.1186/s12916-025-04214-8. PMID: 40598460; PMCID: PMC12220625.
(14) Miao Y, Chen S, Zhang X, Jin J, Xu R, Daly I, Jia J, Wang X, Cichocki A, Jung TP. BCI-Based Rehabilitation on the Stroke in Sequela Stage. Neural Plast. 2020 Dec 13;2020:8882764. doi: 10.1155/2020/8882764. PMID: 33414824; PMCID: PMC7752268.
(15) Mihara M, Hattori N, Hatakenaka M, Yagura H, Kawano T, Hino T, Miyai I. Near-infrared spectroscopy-mediated neurofeedback enhances efficacy of motor imagery-based training in poststroke victims: a pilot study. Stroke. 2013 Apr;44(4):1091-8. doi: 10.1161/STROKEAHA.111.674507. Epub 2013 Feb 12. PMID: 23404723.
(16) Ramos-Murguialday A, Broetz D, Rea M, Läer L, Yilmaz O, Brasil FL, Liberati G, Curado MR, Garcia-Cossio E, Vyziotis A, Cho W, Agostini M, Soares E, Soekadar S, Caria A, Cohen LG, Birbaumer N. Brain-machine interface in chronic stroke rehabilitation: a controlled study. Ann Neurol. 2013 Jul;74(1):100-8. doi: 10.1002/ana.23879. Epub 2013 Aug 7. PMID: 23494615; PMCID: PMC3700597.
(17) Wang A, Tian X, Jiang D, Yang C, Xu Q, Zhang Y, Zhao S, Zhang X, Jing J, Wei N, Wu Y, Lv W, Yang B, Zang D, Wang Y, Zhang Y, Wang Y, Meng X. Rehabilitation with brain-computer interface and upper limb motor function in ischemic stroke: A randomized controlled trial. Med. 2024 Jun 14;5(6):559-569.e4. doi: 10.1016/j.medj.2024.02.014. Epub 2024 Apr 19. PMID: 38642555.
(18) Liu X, Zhang W, Li W, Zhang S, Lv P, Yin Y. Effects of motor imagery based brain-computer interface on upper limb function and attention in stroke patients with hemiplegia: a randomized controlled trial. BMC Neurol. 2023 Mar 31;23(1):136. doi: 10.1186/s12883-023-03150-5. PMID: 37003976; PMCID: PMC10064693.
(19) Ma ZZ, Wu JJ, Hua XY, Zheng MX, Xing XX, Ma J, Shan CL, Xu JG. Evidence of neuroplasticity with brain-computer interface in a randomized trial for post-stroke rehabilitation: a graph-theoretic study of subnetwork analysis. Front Neurol. 2023 Jun 6;14:1135466. doi: 10.3389/fneur.2023.1135466. PMID: 37346164; PMCID: PMC10281191.
(20) Li X, Wang L, Miao S, Yue Z, Tang Z, Su L, Zheng Y, Wu X, Wang S, Wang J, Dou Z. Sensorimotor Rhythm-Brain Computer Interface With Audio-Cue, Motor Observation and Multisensory Feedback for Upper-Limb Stroke Rehabilitation: A Controlled Study. Front Neurosci. 2022 Mar 11;16:808830. doi: 10.3389/fnins.2022.808830. PMID: 35360158; PMCID: PMC8962957.
(21) Wu Q, Yue Z, Ge Y, Ma D, Yin H, Zhao H, Liu G, Wang J, Dou W, Pan Y. Brain Functional Networks Study of Subacute Stroke Patients With Upper Limb Dysfunction After Comprehensive Rehabilitation Including BCI Training. Front Neurol. 2020 Jan 27;10:1419. doi: 10.3389/fneur.2019.01419. PMID: 32082238; PMCID: PMC7000923.
(22) Pichiorri F, Morone G, Petti M, Toppi J, Pisotta I, Molinari M, Paolucci S, Inghilleri M, Astolfi L, Cincotti F, Mattia D. Brain-computer interface boosts motor imagery practice during stroke recovery. Ann Neurol. 2015 May;77(5):851-65. doi: 10.1002/ana.24390. Epub 2015 Mar 27. PMID: 25712802.

Table E12.1. Inclusion status of included studies across outcome and supplementary analyses

No.	Study	FMA-UE main NMA	ARAT analysis	WMFT analysis	Stroke-stage subgroup	Sensitivity analysis	Remarks
1	Ang 2014a	Yes	No	No	Chronic	Yes	BCI-Robot vs Robot
2	Ang 2014b	Yes	No	No	Chronic	Yes	Three-arm trial including BCI-Robot, Robot, and UR
3	Cantillo-Negrete 2025	Yes	Yes	No	Not included	No	Mixed-stage study; BCI-Robot vs shamBCI-Robot
4	Chen 2020	Yes	No	No	Subacute	Yes	BCI-Robot vs MI
5	Cheng 2020	Yes	Yes	No	Chronic	Yes	BCI-MM vs Robot
6	Frolov 2017	Yes	Yes	No	Not included	No	Mixed-stage study; BCI-Robot vs shamBCI-Robot
7	Fu 2023	Yes	No	No	Subacute	Yes	BCI-Robot vs UR
8	Guo 2022	Yes	No	Yes	Chronic	Yes	Three-arm trial including UR, Robot, and BCI-Robot
9	He 2025	Yes	No	No	Subacute	Yes	BCI-MM vs shamBCI-MM
10	Hu 2021	Yes	Yes	No	Not included	No	Mixed-stage study
11	Ji 2025	Yes	Yes	No	Subacute	Yes	BCI-Robot vs Robot
12	Kim 2025	Yes	No	No	Chronic	Yes	BCI-MM vs shamBCI-MM
13	Lu 2025	Yes	Yes	No	Chronic	Yes	BCI-MM vs MI
14	Miao 2020	Yes	No	No	Chronic	Yes	BCI-FES vs UR
15	Mihara 2013	Yes	Disconnected	No	Subacute	Yes	ARAT comparison was disconnected from the main ARAT network
16	Ramos-Murguialday 2013	Yes	No	No	Chronic	Yes	BCI-Robot vs shamBCI-Robot
17	Wang 2024	Yes	Yes	Yes	Subacute	Yes	BCI-MM vs UR
18	Liu 2023	Yes	No	Single-study comparison	Not included	Yes	Disconnected in the subacute subgroup network; BCI-FES vs FES.
19	Ma 2023	Yes	No	No	Not included	No	Mixed-stage study
20	Li 2022	Yes	No	Yes	Not included	No	Mixed-stage study; included in WMFT pairwise meta-analysis
21	Wu 2020	Yes	Yes	Yes	Subacute	Yes	BCI-MM vs UR
22	Pichiorri 2015	Yes	No	No	Subacute	Yes	BCI-visual vs shamBCI-visual

Note: FMA-UE main NMA indicates inclusion in the primary network meta-analysis for the FMA-UE outcome. ARAT analysis and WMFT analysis indicate inclusion in the corresponding secondary outcome analyses. "Disconnected" indicates that the study reported the outcome but was not connected to the main evidence network for that outcome. Stroke-stage subgroup indicates inclusion in the subacute or chronic subgroup analysis; studies with mixed or unclear stroke stages were not included in the stroke-stage subgroup analysis unless data could be assigned to a specific phase. Sensitivity analysis indicates inclusion after excluding mixed-stage studies.
